# Impact of multicomponent integrated care on mortality and hospitalization after acute coronary syndrome: a systematic review and meta-analysis

**DOI:** 10.1093/ehjqcco/qcac032

**Published:** 2022-06-10

**Authors:** Jia-Xin Hoo, Ya-Feng Yang, Jia-Yin Tan, Jingli Yang, Aimin Yang, Lee-Ling Lim

**Affiliations:** Department of Medicine, Faculty of Medicine, University of Malaya, 50603 Kuala Lumpur, Malaysia; Department of Medicine, Faculty of Medicine, University of Malaya, 50603 Kuala Lumpur, Malaysia; Department of Medicine, Faculty of Medicine, University of Malaya, 50603 Kuala Lumpur, Malaysia; College of Earth and Environmental Sciences, Lanzhou University, 73000 Lanzhou, China; School of Public Health and Social Work, Queensland University of Technology, 4000 Brisbane, QLD Australia; Department of Medicine and Therapeutics, The Chinese University of Hong Kong, Hong Kong SAR, China; Department of Medicine, Faculty of Medicine, University of Malaya, 50603 Kuala Lumpur, Malaysia; Department of Medicine and Therapeutics, The Chinese University of Hong Kong, Hong Kong SAR, China; Asia Diabetes Foundation, Hong Kong SAR, China

**Keywords:** Myocardial ischaemia, Hospitalization, Death, Patient care team, Integrated delivery system, Care model

## Abstract

**Aims:**

Multicomponent integrated care is associated with sustained control of multiple cardiometabolic risk factors among patients with type 2 diabetes. There is a lack of data in patients with acute coronary syndrome (ACS). We aimed to examine its efficacy on mortality and hospitalization outcomes among patients with ACS in outpatient settings.

**Methods and results:**

A literature search was conducted on PubMed, EMBASE, Ovid, and Cochrane library databases for randomized controlled trials, published in English language between January 1980 and November 2020. Multicomponent integrated care defined as two or more quality improvement strategies targeting different domains (the healthcare system, healthcare providers, and patients) for one month or more. The study outcomes were all-cause and cardiovascular-related mortality, hospitalization, and emergency department visits. We pooled the risk ratio (RR) with 95% confidence interval (CI) for the association between multicomponent integrated care and study outcomes using the Mantel–Haenszel test. 74 trials (*n* = 93 278 patients with ACS) were eligible. The most common quality improvement strategies were team change (83.8%), patient education (62.2%), and facilitated patient-provider relay (54.1%). Compared with usual care, multicomponent integrated care was associated with reduced risks for all-cause mortality (RR 0.83, 95% CI 0.77–0.90; *P* < 0.001; *I*^2^ = 0%), cardiovascular mortality (RR 0.81, 95% CI 0.73–0.89; *P* < 0.001; *I*^2^ = 24%) and all-cause hospitalization (RR 0.88, 95 % CI, 0.78–0.99; *P* = 0.040; *I*^2^ = 58%). The associations of multicomponent integrated care with cardiovascular-related hospitalization, emergency department visits and unplanned outpatient visits were not statistically significant.

**Conclusion:**

In outpatient settings, multicomponent integrated care can reduce risks for mortality and hospitalization in patients with ACS.

## Graphical Abstract

**Graphical Abstract:**
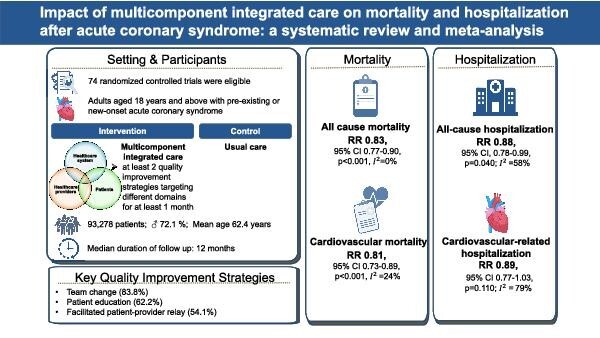
Impact of multicomponent integrated care on mortality and hospitalization after acute coronary syndrome: a systematic review and meta-analysis.

## Introduction

Acute coronary syndrome (ACS) is typically presented with three main subtypes namely non-ST-elevation myocardial infarction (NSTEMI), ST-elevation myocardial infarction (STEMI), and unstable angina.^1^ According to the World Health Organization, the leading cause of death was coronary heart disease which accounted for 16% of total mortality in 2019.^2^ Its disease burden rises with ageing, resulting in increasing disability, functional decline, and healthcare costs.^3^

Despite the availability of organ-protective medications and percutaneous coronary intervention (PCI) in past few decades,^1,4^ the clinical outcomes of patients with coronary heart disease in low- and middle-income countries have been suboptimal.^5–7^ In the Malaysian general population, the leading cause of death was coronary heart disease (17%), followed by pneumonia (11.4%), and cerebrovascular disease (8.3%) based on the 2020 Statistics on Causes of Death.^8^

The attained levels of modifiable cardiometabolic risk factors can influence the onset and progression of ACS.^9–12^ Apart from the overburdened health care system, fragmented care may reduce the service quality among patients after the onset of ACS with increased health care expenditure.^13,14^ In the 2014–15 Malaysian Acute Coronary Syndrome Registry, the proportions of patients with ACS who reported having diabetes, hypertension, and dyslipidaemia were 43.2%, 60.8%, and 35.9%, respectively.^15^ This highlights the challenges in translating evidence into real-world practice with significant gaps in diagnosis, treatment, and monitoring.

Multicomponent integrated care can be the key to relieve the overburdened healthcare systems. It is defined as the implementation of two or more quality improvement (QI) strategies from different domains targeting the healthcare system, healthcare providers and patients.^16,17^ Our previous meta-analysis involving 181 randomized clinical trials (RCTs) confirmed the sustained improvements in multiple cardiometabolic risk factors in patients with type 2 diabetes with the implementation of multicomponent integrated care.^16^ Herein, we performed a meta-analysis to examine the efficacy of multicomponent integrated care on mortality and hospitalization outcomes among patients with ACS in outpatient settings.

## Research design and methods

### Data sources and searches

We performed literature search on PubMed, EMBASE, Ovid, and The Cochrane Library databases for RCTs on multicomponent integrated care and ACS, which were published in English language between January 1980 (inception) and November 2020. The Medical Subject Headings (MeSH) search terms are listed in the supplementary material online, *Table S1*. We reported the results in accordance with the Preferred Reporting Items for Systematic reviews and Meta-Analyses (PRISMA) guideline. The protocol is prospectively registered on International Prospective Register of Systematic Reviews (CRD42020220107).

### Study selection

Based on the PICO framework, we included RCTs which recruited at least 100 adults aged 18 years or above with either pre-existing or new-onset ACS managed in outpatient settings, with the implementation of multicomponent integrated care for a minimum of 1 month. Similar to that in our previous work,^16^ we defined multicomponent integrated care as the implementation of two or more QI strategies from two out of three domains namely the healthcare system, healthcare providers, and patients (Supplementary material online, *Table S2*).

The primary outcome was mortality (all-cause and cardiovascular-related). The secondary outcomes were hospitalization (all-cause and cardiovascular-related), health care utilisation (emergency department visits and unscheduled outpatient visits), medication prescription, and the control of cardiometabolic risk factors.

### Data extraction and quality assessment

Two authors (J.X.H and Y.F.Y) independently reviewed all publication titles and abstracts to exclude articles not meeting the eligibility criteria. Using a standardised case record form, two authors extracted data including sociodemographic, healthcare settings, sample size, characteristic of study population (age, gender, ethnicity, and cardiometabolic risk factors), duration of intervention, type of QI strategies, outcomes of interest, and medication prescription. Using the Cochrane Effective Practice and Organisation of Care (EPOC) risk of bias tool, the two authors independently appraised the quality of studies and risk of bias. Any disagreement was resolved by a senior investigator (L.L.L).

### Data synthesis and analysis

To explore the effect of multicomponent integrated care, we determined the net intervention with consideration of the number and type of QI strategies, as well as the outcome measures, between the intervention and control arms. In the present meta-analysis, there were seven RCTs with >2-arm study design, in which the intervention arm with the highest number of QI strategies was selected.

We calculated the risk ratios (RR) and mean post-interventional differences with 95% confidence intervals (CI) for binary and continuous outcomes, respectively. We pooled the available trial-level data to estimate measurements of central tendency and dispersion. Continuous variables that were reported as median [interquartile range (IQR)] were converted to mean ± standard deviation (SD) following the established method.^18,19^ We pooled the risk ratio (RR) with 95% confidence interval (CI) for the association between multicomponent integrated care and clinical outcomes using the Mantel–Haenszel test. We performed *I^2^* statistic and funnel plots to assess study heterogeneity and publication bias, respectively.^18^ An *I^2^*statistic of >50% was considered significant study heterogeneity and therefore, random-effect model was used. On the other hand, fixed-effect model was performed for *I^2^*statistic of <50%.

We performed two sensitivity analyses: (1) stratified by median duration of follow-up (≤12 months vs. >12 months) and (2) excluded RCTs that were conducted before 2010 as usual care might have transformed over time. To explore the significance of the differences in RR and the possible influence of confounding factors, we performed meta-regression and subgroup analyses for possible sources of heterogeneity including age, gender, and use of organ-protective drugs (antiplatelet/antithrombotic therapy, RAAS inhibitors, and lipid-lowering therapy).^20^ All statistical analyses were performed using RevMan software version 5.4.1 and R version 4.1.3 (‘metareg’ package). A two-sided *P* <0.05 denoted statistical significance.

## Results

### Study characteristics

**Figure 1:**
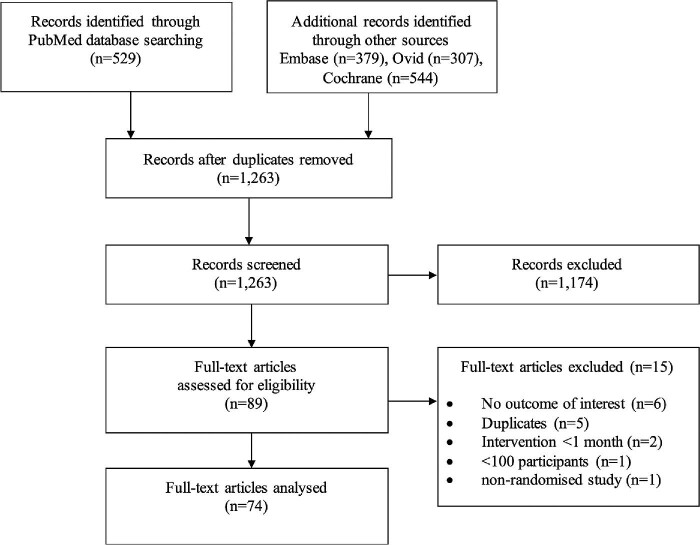
Preferred Reporting Items for Systematic reviews and Meta-Analyses flow diagram.

*Figure 1* shows the PRISMA flow diagram. The database search generated 1759 citations. After removal of duplicates, 1263 articles underwent abstract and title screening. A total of 74 RCTs (*n* = 93 278 patients) fulfilled the eligibility criteria and were analysed.

Baseline characteristics of the RCTs included in the meta-analysis are listed in the supplementary material online, *Table S3*. The mean ± SD age of the study population was 62.4 ± 11.9 years and 67 253 (72.1%) were males. A total of 15 (20.3%) RCTs were conducted in Asia, 29 (39.2%) in Europe, 17 (22.9%) in North America, and 13 (17.6%) in other countries/regions. The median duration of intervention was 6 months (IQR 3–10). The median number of QI strategies implemented was 4 (IQR 2–7). The most common QI strategies were team change (83.8%), patient education (62.2%), and facilitated relay (54.1%) (Supplementary material online, *Table S3*).

The assessment of study quality is presented in the supplementary material online, *Figure S1*. The majority of included RCTs were of low risk of bias. There were 31 (41.9%) RCTs with performance bias due to a lack of blinding of participants and study personnel, followed by attrition bias in 18 (24.3%) RCTs.

### Effects of multicomponent integrated care on mortality and hospitalization post-discharge for acute coronary syndrome

**Figure 2:**
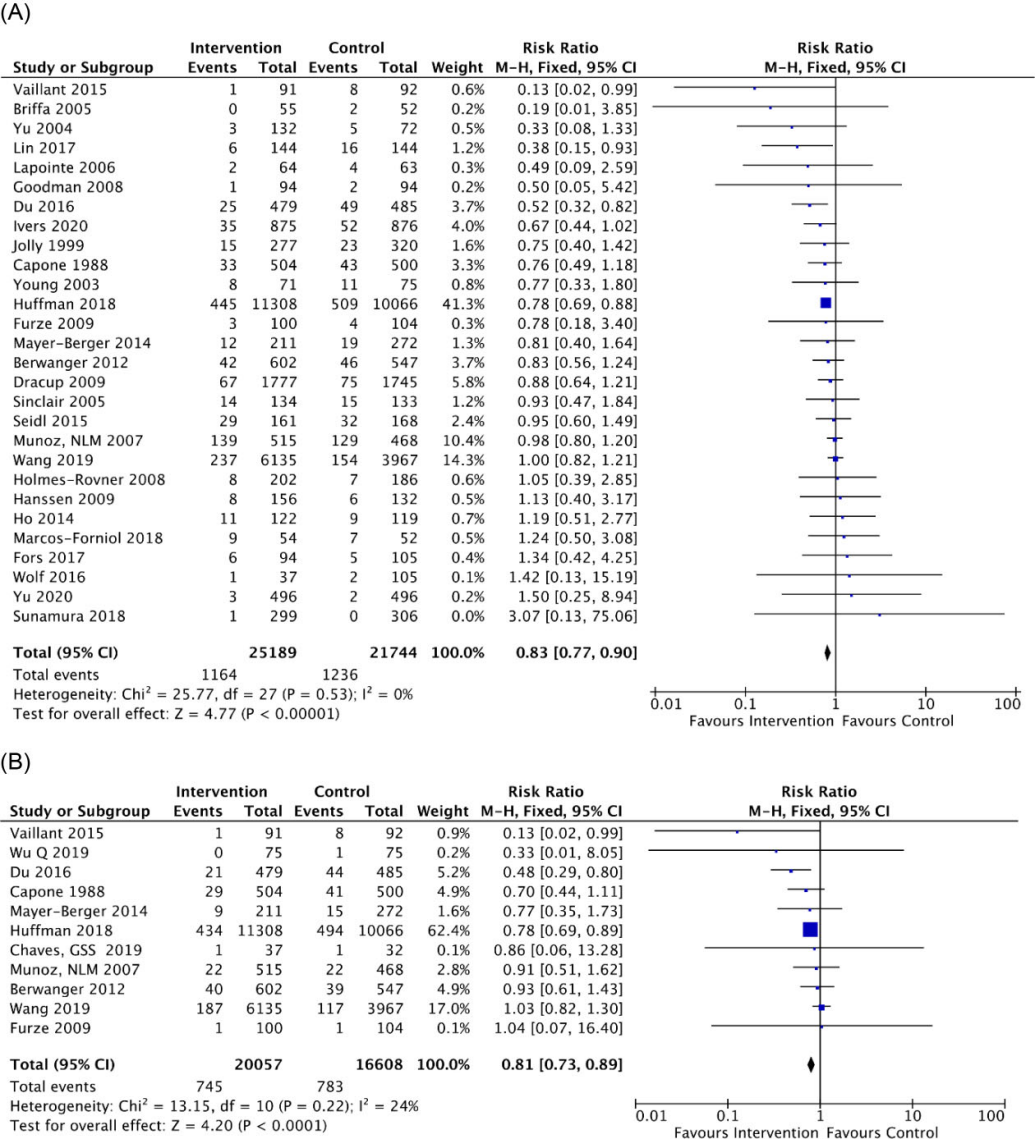
Meta-analysis results of the effects of multicomponent integrated care on (*A*) all-cause mortality and (*B*) cardiovascular-related mortality among patients with acute coronary syndrome. Forest plots were derived from fixed-effects meta-analysis models. M–H, Mantel–Haenszel method; 95% CI, 95% confidence interval.

A total of 28 RCTs reported all-cause mortality post-discharge for ACS, showing a pooled RR of 0.83 (95% CI, 0.77–0.90, *P* <0.001; *I*^2^ = 0%) (*Figure 2A*). Overall, the corresponding pooled RR for cardiovascular-related mortality was 0.81 (95% CI 0.73–0.89, *P* <0.001; *I*^2^ = 24%) (*Figure 2B*). When stratified by median duration of follow-up, the pooled RR for all-cause mortality was 0.88 (95% CI 0.77–1.01, *P* =0.060; *I^2^* = 0%) with ≤12 months of follow-up and 0.80 (95% CI 0.73–0.88, *P* <0.001; *I^2^* = 0%) with >12 months of follow-up (Supplementary material online, *Figure S2A*). Regarding cardiovascular-related mortality, the pooled RR was 0.92 (95% CI 0.77–1.10, *P* = 0.380; *I^2^* = 5%) for ≤12 months of follow-up and 0.77 (95% CI 0.68–0.86, *P* <0.001; *I^2^* = 18%) for >12 months of follow-up (Supplementary material online, *Figure S2B*). After excluding RCTs conducted before 2010, multicomponent integrated care was still associated with reduced risk for all-cause mortality (RR 0.81, 95% CI 0.74–0.89, *P* <0.001; *I^2^* = 26%) and cardiovascular-related mortality (RR 0.81, 95% CI 0.73–0.90, *P* <0.001; *I^2^* = 44%) (Supplementary material online, *Figure S3*).

**Figure 3:**
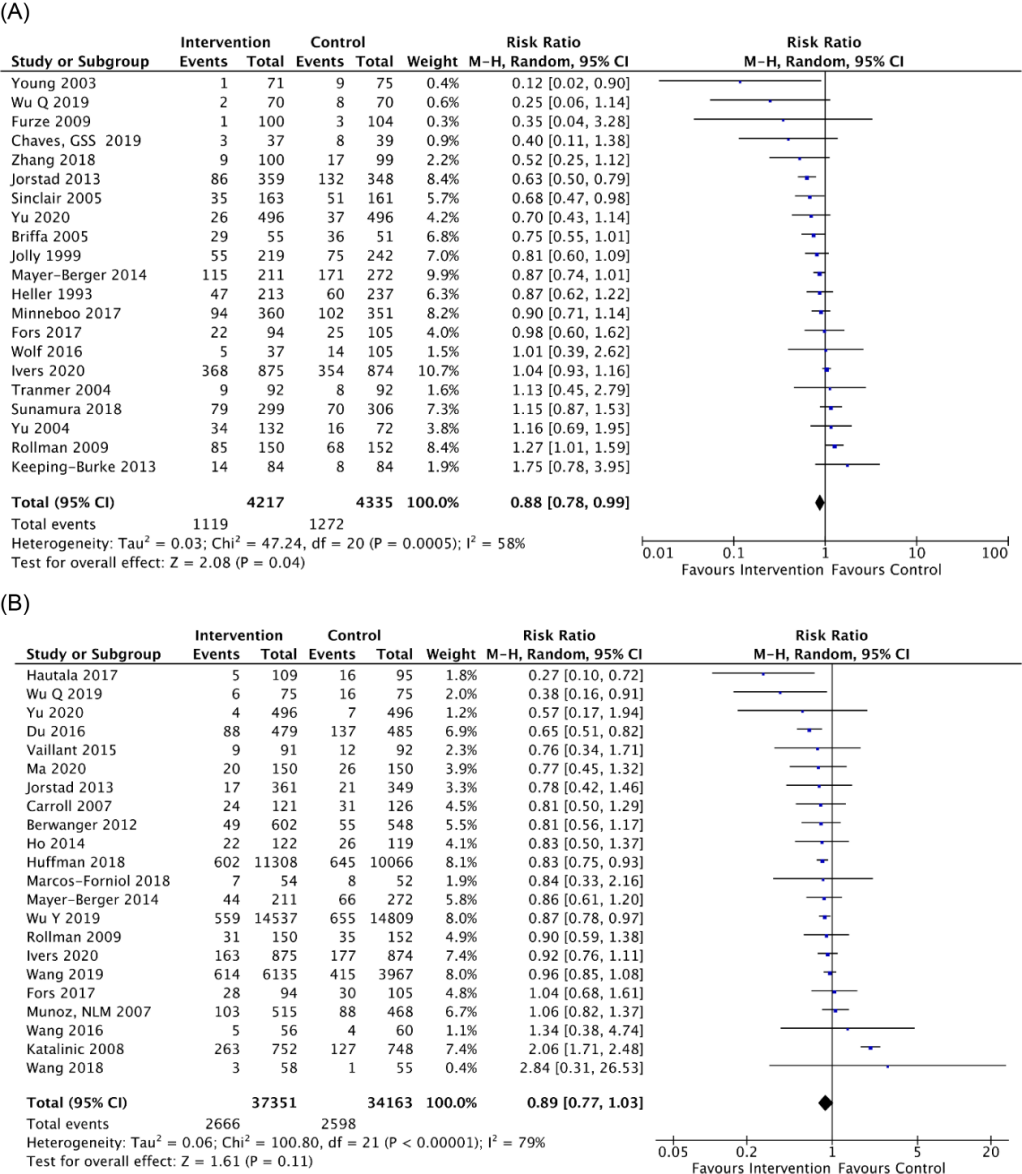
Meta-analysis results of multicomponent integrated care on (*A*) all-cause hospitalization and (*B*) cardiovascular-related hospitalization among patients with acute coronary syndrome. Forest plots were derived from random-effects meta-analysis models. M–H, Mantel–Haenszel method; 95% CI, 95% confidence interval.

A total of 21 RCTs reported all-cause hospitalization. In a random-effect model, the pooled RR was 0.88 (95% CI 0.78–0.99, *P* = 0.040; *I*^2^ = 58%) (*Figure 3A*). Among the 22 RCTs which reported cardiovascular-related hospitalization, the pooled RR was 0.89 (95% CI, 0.77–1.03, *P* = 0.110; *I*^2^ =  79%) (*Figure 3B*). When stratified by median duration of follow-up, the pooled RR for all-cause hospitalization was 0.81 (95% CI 0.69–0.95, *P* = 0.010; *I^2^* = 64%) with ≤12 months of follow-up and 1.03 (95% CI 0.84–1.26, *P* = 0.810; *I^2^* = 38%) with >12 months of follow-up (Supplementary material online, *Figure S4A*). Regarding cardiovascular-related hospitalization, the pooled RR was 0.87 (95% CI 0.68–1.12, *P* = 0.290; *I^2^* = 81%) for ≤12 months of follow-up and 0.85 (95% CI 0.77–0.94, *P* = 0.001; *I^2^* = 35%) for >12 months of follow-up (Supplementary material online, *Figure S4B*). After excluding RCTs conducted before 2010, multicomponent integrated care was not significantly associated with reduced risk for all-cause hospitalization (RR 0.87, 95% CI 0.74–1.02, *P* = 0.090; *I^2^* = 62%), but it was statistically significant for cardiovascular-related hospitalization (RR 0.86, 95% CI 0.81–0.91, *P* <0.001; *I^2^* = 21%) (Supplementary material online, *Figure S5*).

The associations of multicomponent integrated care with emergency department visits (RR 0.98, 95% CI 0.81–1.19; *P* = 0.860; *I*^2^ = 66%) (Supplementary material online, *Figure S6*) and unplanned outpatient visits (RR 1.03, 95% CI 0.99–1.08; *P* = 0.160; *I*^2^ = 40%) (Supplementary material online, *Figure S7*) were not statistically significant.

### Effects of individual quality improvement strategy on mortality and hospitalization post-discharge for acute coronary syndrome

**Figure 4:**
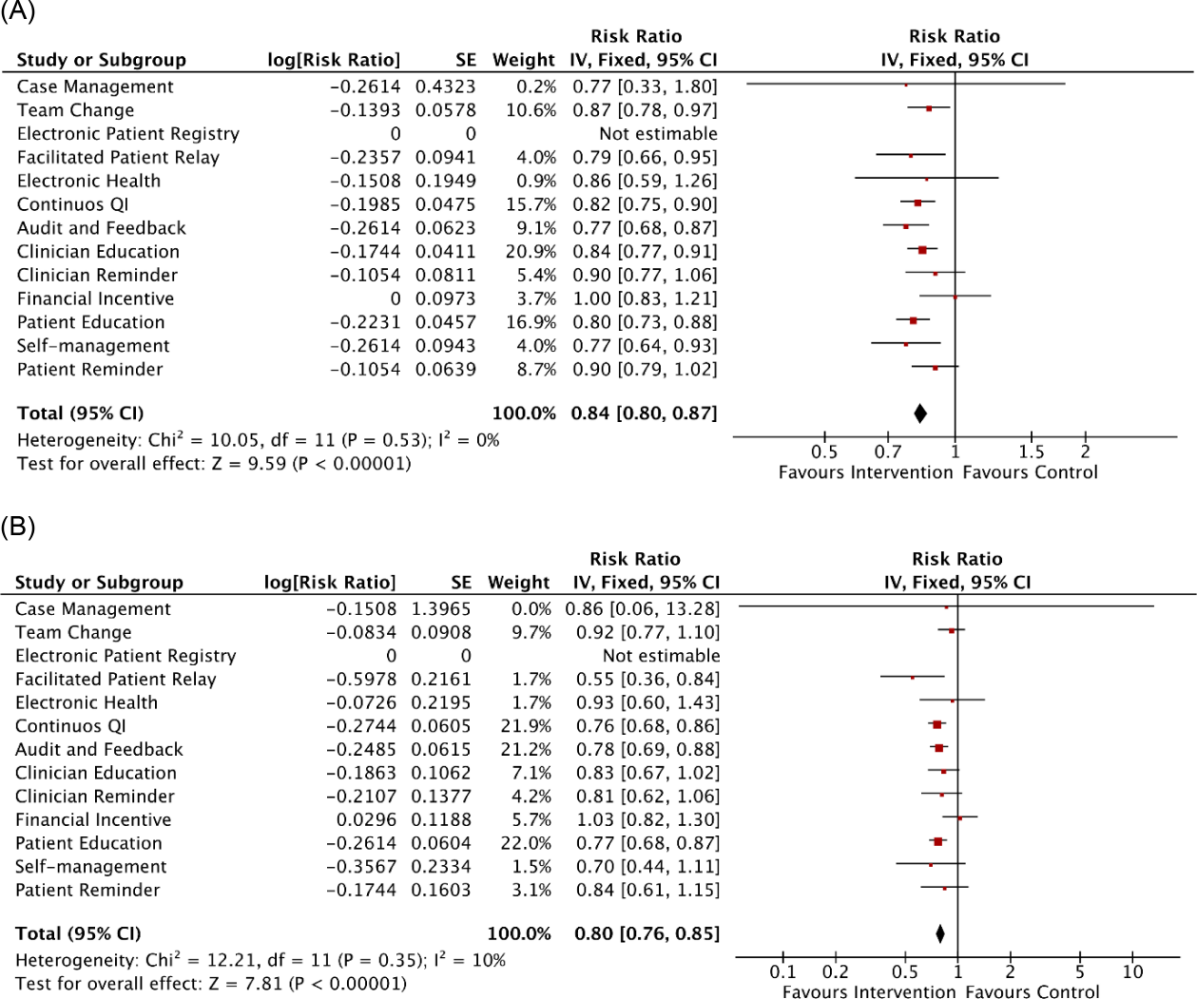
Meta-analysis results of the effects of individual quality improvement strategy on (*A*) all-cause mortality and (*B*) cardiovascular-related mortality among patients with acute coronary syndrome. Forest plots were derived from fixed-effects meta-analysis models. SE, standard error; 95% CI, 95% confidence interval; Continuous QI, continuous quality improvement.

Among 12 QI strategies, team change (RR 0.87, 95% CI 0.78–0.97; 24 RCTs), facilitated relay (RR 0.79, 95% CI 0.66–0.95; 13 RCTs), continuous QI (RR 0.82, 95% CI 0.75–0.90; 5 RCTs), audit and feedback (RR 0.77, 95% CI 0.68–0.87; 3 RCTs), clinician education (RR 0.84, 95% CI 0.77–0.91; 12 RCTs), patient education (RR 0.80, 95% CI 0.73–0.88; 15 RCTs), and self-management (RR 0.77, 95% CI 0.64–0.93; 11 RCTs) significantly reduced the risk for all-cause mortality (*Figure 4A*). Regarding the risk for cardiovascular mortality, facilitated relay (RR 0.55, 95% CI 0.36–0.84; 4 RCTs), continuous QI (RR 0.76, 95% CI 0.68–0.86; 4 RCTs), audit and feedback (RR 0.78, 95% CI 0.69–0.88; 1 RCT), and patient education (RR 0.77, 95% CI 0.68–0.87; 7 RCTs) were the key QI strategies (*Figure 4B* and Supplementary material online*, Table S4*).

**Figure 5:**
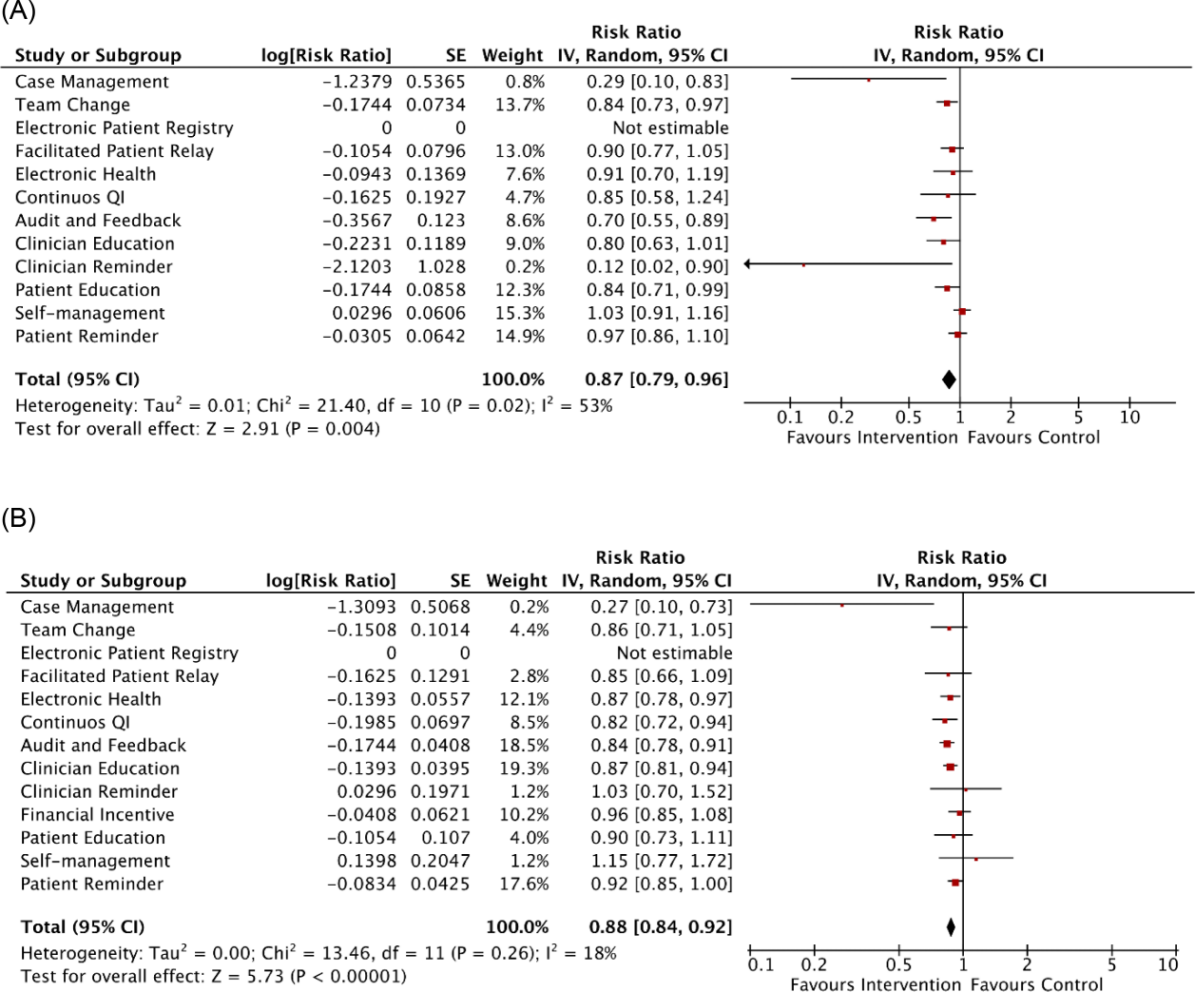
Meta-analysis results of the effects of individual QI strategy on (*A*) all-cause hospitalization and (*B*) cardiovascular hospitalization among patients with acute coronary syndrome. Forest plots were derived from random-effects meta-analysis models. SE, standard error; 95% CI, 95% confidence interval; Continuous QI, continuous quality improvement.

For all-cause hospitalization, case management (RR 0.29, 95% CI 0.10–0.83; 2 RCTs), team change (RR 0.84, 95% CI 0.73–0.97; 17 RCTs), audit and feedback (RR 0.70, 95% CI 0.55–0.89; 2 RCTs), clinician reminder (RR 0.12, 95% CI 0.02–0.90; 1 RCT), and patient education (RR 0.84, 95% CI 0.71–0.99; 12 RCTs) were the key QI strategies (*Figure 5A*). On the other hand, case management (RR 0.27, 95% CI 0.10–0.73; 1 RCT), electronic health (RR 0.87, 95% CI 0.78–0.97; 5 RCTs), continuous QI (RR 0.82, 95% CI 0.72–0.94; 6 RCTs), audit and feedback (RR 0.84, 95% CI 0.78–0.91; 3 RCTs), and clinician education (RR 0.87, 95% CI 0.81–0.94; 10 RCTs) were associated with a reduced risk for cardiovascular-related hospitalization (*Figure 5B*).

### Effects of multicomponent integrated care on the changes of medications and cardiometabolic risk factors

Multicomponent integrated care was associated with increased rates of medication prescriptions, showing an RR of 1.17 (95% CI 1.08–1.27, *P* <0.001; *I*^2^ = 99%) for antiplatelet/antithrombotic therapy, RR 1.13 (95% CI 1.02–1.27, *P* = 0.020; *I*^2^ = 91%) for renin-angiotensin-aldosterone system inhibitor and RR 1.13 (95% CI 1.03–1.23, *P* = 0.008; *I*^2^ = 96%) for lipid-lowering therapy (Supplementary material online, *Table S5* and *Figure S8*).

In addition, multicomponent integrated care was associated with improvements in the diastolic blood pressure (mean difference −1.64 mmHg, 95% CI −3.09 to −0.20, *P* <0.030; *I*^2^ = 60%), total cholesterol (mean difference −0.23 mmol/L, 95% CI −0.43 to −0.04, *P* <0.02; *I*^2^ = 82%), low-density lipoprotein (LDL) cholesterol (mean difference −0.18 mmol/L, 95% CI −0.29 to −0.07, *P* <0.002; *I*^2^ = 78%), triglyceride (mean difference −0.17 mmol/L, 95% CI −0.30 to −0.03, *P* <0.02; *I*^2^ = 82%), and body mass index (BMI) (mean difference −0.83 kg/m^2^, 95% CI −1.05 to −0.61, *P* <0.001; *I*^2^ = 49%) (Supplementary material online, *Figure S9*). There were no significant improvements with glycaemia and systolic blood pressure (Supplementary material online, *Figure S9*).

### Heterogeneity analysis

We conducted meta-regression analysis based on the results of meta-analysis to determine potential sources of heterogeneity. As shown in the supplementary material online *Table S6*, use of antiplatelet/antithrombotic therapy might influence the effect of multicomponent integrated care on cardiovascular-related mortality. When the proportion of antiplatelet/antithrombotic therapy was >50%, multicomponent integrated care was associated with a pooled RR of 0.79 (95% CI 0.70–0.89) for cardiovascular-related mortality (Supplementary material online, *Figure S10*). Regarding the risk for all-cause hospitalization, mean age ≥60 years (RR 0.81, 95% CI 0.69–0.94), >90% proportional use of antiplatelet/antithrombotic therapy (RR 0.81, 95% CI 0.66–1.00), and <90% proportional use of lipid-lowering drugs at baseline (RR 0.71, 95% CI 0.55–0.92) were associated with reduced risk for all-cause hospitalization (Supplementary material online, *Figure S11*). Mean age ≥60 years was also associated with reduced risk for cardiovascular-related hospitalization (RR 0.85, 95% CI 0.77–0.93) (Supplementary material online, *Figure S12*).

## Discussion

To the best of our knowledge, this is the largest meta-analysis investigating the effects of multicomponent integrated care on mortality and hospitalization among patients with ACS managed in the outpatient settings. We reported that the implementation of multicomponent integrated care for at least one month was associated with a 11–19% reduced risk for mortality and hospitalization among patients with ACS, particularly in those aged ≥60 years, with >50% use of antiplatelet/antithrombotic therapy and <90% use of lipid-lowering therapy at baseline. We also reported greater risk reductions with a longer duration of follow-up, highlighting that ongoing multicomponent integrated care program is needed to sustain clinical benefits.

Inter-professional collaboration has become an essential skill to meet complex care needs of patients with chronic health conditions including ACS. Compared to published reviews, the present meta-analysis was the largest by including 74 RCTs involving 93 278 patients with ACS.^21–24^ Furthermore, the present meta-analysis utilized a stricter definition of multicomponent integrated care (defined as two or more QI strategies from different health domains), whereas previous reviews included those with single domain.

Compared with usual care, the present meta-analysis reported that patients receiving multicomponent integrated care had higher medication prescription rate and improved levels of diastolic blood pressure, lipid profile, and BMI. A meta-analysis of 52 RCTs involving adults receiving cholesterol treatment reported that every 1 mmol/L reduction in LDL-cholesterol was associated with a 19% relative risk reduction for major adverse cardiovascular events including cardiovascular mortality, non-fatal myocardial infarction, non-fatal ischaemic stroke, and coronary revascularization.^25^ Another meta-analysis of 54 RCTs involving adults with obesity also reported that weight loss interventions were associated with an 18% relative risk reduction for all-cause mortality, but not for cardiovascular mortality.^26^ In the present meta-analysis, fasting plasma glucose and systolic blood pressure were not significantly improved likely due to lower levels at baseline (median 6.1 mmol/L and 129 mmHg, respectively).

After an initial presentation of ACS, there is an increased risk for recurrence with shortened survival, especially in the presence of sub-optimally controlled cardiometabolic risk factors.^11,27^ Moreover, the complexity of treatment regimen in patients with chronic diseases is a barrier to adherence to guideline-directed medical therapy.^28^ As shown in the present meta-analysis, multicomponent integrated care can enhance patient-provider communication and care continuity, as well as promote patient education and empowerment for better self-management, resulting in improved adherence to guideline-directed medical therapy and control of multiple cardiometabolic risk factors.^29^ In our subgroup analysis, multicomponent integrated care was particularly effective in those with >50% use of antiplatelet/antithrombotic therapy and <90% use of lipid-lowering therapy at baseline, likely due to its impact in overcoming therapeutic inertia among healthcare providers, as well as in promoting self-management among these very high-risk patients.

Patients with ACS tend to have recurrent hospitalizations especially in the first 12 months post-discharge. This is likely due to infections, comorbidities, and medication-related adverse events such as bleeding.^30^ Hence, post-discharge treatment plan is crucial to improve clinical outcomes, prevent hospitalization, and mortality among patients with ACS.^31,32^ Among 12 quality strategies, case management, team-based care (involving allied health personnel such as nurses, pharmacists, and dietitians), and facilitated patient-provider communication (using either the manual/electronic tools, allied health personnel, or peer supporters) are most effective in preventing mortality and hospitalization due to any causes among patients with ACS. Our results are consistent with a meta-analysis involving patients with type 2 diabetes.^16^

In contrast to patients with type 2 diabetes,^16^ continuous QI, audit and feedback, and clinician education were the additional QI strategies that could reduce risk for mortality and hospitalization in patients with ACS. Several studies and disease registries had shown that up to 40% of patients with ACS had coexisting diabetes.^4,33,34^ As such, compared with those with type 2 diabetes, patients with ACS are likely to have a more complex clinical course needing multicomponent integrated care with continuous refinement of the approach, along with audit and feedback at the system- and provider-level. In addition, clinician education is important as the management of ACS has progressed thus far. Decision-making is dependent on the patient's history, haemodynamic stability and timing of initiation, and risk-benefit ratio of available therapies (pharmacological approach vs. coronary revascularization).^35^

Our main strength is by performing an updated meta-analysis of RCTs examining the efficacy of multicomponent integrated care on mortality and hospitalization among patients with ACS in outpatient settings. We also used stricter criteria for defining multicomponent integrated care to emphasize the need for interdisciplinary collaboration in managing patients with chronic diseases including ACS. Given that usual care might transform over time, we reported consistent benefits on mortality and hospitalization with multicomponent integrated care after excluding RCTs conducted before 2010.

We acknowledge several study limitations. First, classification of QI strategies was challenging as they were not clearly defined in low-quality RCTs. Second, as in other RCTs, it was not possible to eliminate selection bias and observation bias in the present meta-analysis. Although the funnel plots showed minor asymmetry corresponding to study heterogeneity (Supplementary material online, *Figure S13*), our results were consistent in several sensitivity and subgroup analyses. Last, this was a trial-level meta-analysis and the lack of access to individual-level data might have limited the robustness of our results. Hence, we were not able to perform subgroup analysis by the status of smoking, diabetes, and obesity.

## Conclusions

Multicomponent integrated care was associated with a reduced risk for mortality and hospitalization among patients after ACS. This was likely due to better treatment adherence and control of multiple cardiometabolic risk factors. Further cost-effectiveness analysis is needed to inform, change, and influence.

## Supplementary Material

qcac032_Supplemental_File

## Data availability

The data that support the findings of this study are available from the corresponding author, Dr Lee-Ling Lim (leeling.lim@ummc.edu.my), upon reasonable request.

## Notes

**PROSPERO registration ID**: CRD42020220107
