# Supplementary material for: Impact of multicomponent integrated care on mortality and hospitalization after acute coronary syndrome: a systematic review and meta-analysis
Source: Eur Heart J Qual Care Clin Outcomes. 2022 Jun 10;9(3):258–67. doi: 10.1093/ehjqcco/qcac032 (PMC10131244; doi:10.1093/ehjqcco/qcac032)
Supplement: qcac032_Supplemental_File [file qcac032_supplemental_file.pdf]

SUPPLEMENTARY MATERIAL

Impact of multicomponent integrated care on mortality and hospitalization after acute coronary syndrome: a systematic review and meta-analysis

	Title	Page(s)
**Figure S1**	Risk of bias assessment	3
**Figure S2**	Meta-analysis results of the effects of multicomponent integrated care on mortality among patients with acute coronary syndrome, stratified by median duration of follow-up	4-5
**Figure S3**	Meta-analysis results of the effects of multicomponent integrated care on mortality among patients with acute coronary syndrome, after excluding trials conducted before 2010	6
**Figure S4**	Meta-analysis results of multicomponent integrated care on hospitalization among patients with acute coronary syndrome, stratified by median duration of follow-up	7-8
**Figure S5**	Meta-analysis results of multicomponent integrated care on hospitalization among patients with acute coronary syndrome, after excluding trials conducted before 2010	9
**Figure S6**	Meta-analysis results of the effects of multicomponent integrated care on emergency department visit	10
**Figure S7**	Meta-analysis results of the effects of multicomponent integrated care on unplanned outpatient visit	11
**Figure S8**	Meta-analysis results of changes of medications	12
**Figure S9**	Meta-analysis results of changes of cardiometabolic risk factors	13-16
**Figure S10**	Association of multicomponent integrated care on cardiovascular-related mortality among patients with acute coronary syndrome by proportion of antiplatelet or antithrombotic therapy	17
**Figure S11**	Association of multicomponent integrated care on all-cause hospitalization among patients with acute coronary syndrome by age groups, proportion of antiplatelet or antithrombotic therapy and proportion of lipid-lowering drugs	18-19
**Figure S12**	Meta-analysis results of multicomponent integrated care on cardiovascular hospitalization among patients with acute coronary syndrome, stratified by age groups	20
**Figure S13**	Funnel plots	21-23
**Table S1**	Search strategy	24
**Table S2**	Definitions of quality improvement strategies in acute coronary syndrome	25-26
**Table S3**	Baseline characteristics of trials included in the meta-analysis	27
**Table S4**	Effects of individual quality improvement strategies on mortality and hospitalization (all-cause and cardiovascular-related) among patients with acute coronary syndrome	28
**Table S5**	Medication use	29

	Title	Page(s)
**Table S6**	Meta-regression of associations between multicomponent integrated care and outcomes among patients with acute coronary syndrome	30
**Table S7**	Study characteristics of trials included in the meta-analysis	31-50

Figure S1. Risk of bias assessment

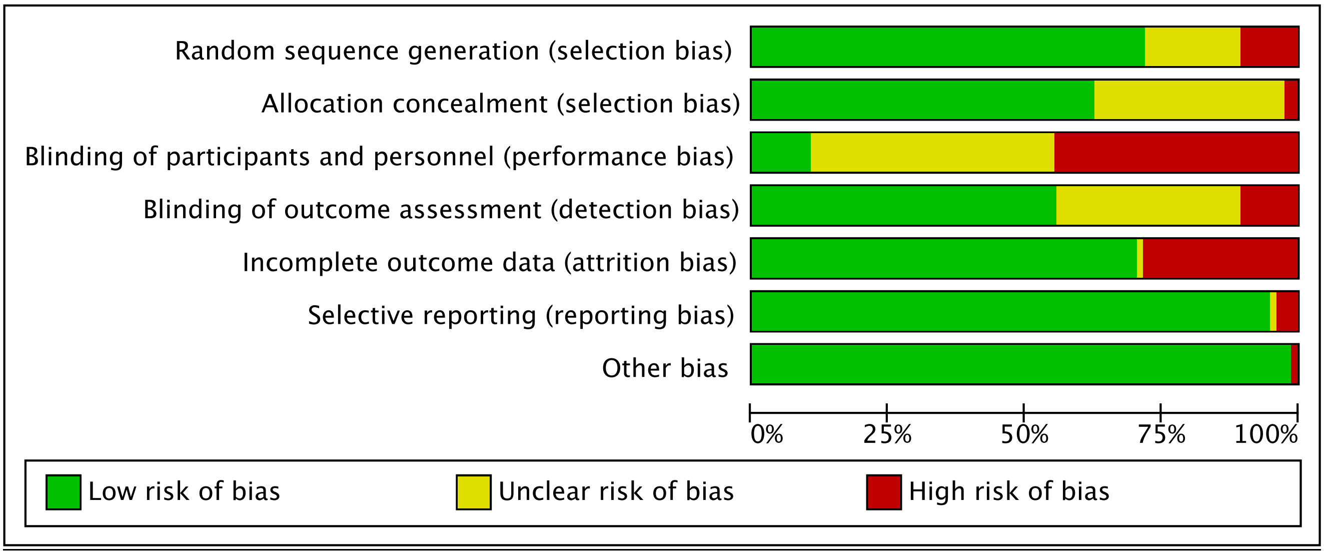

The Cochrane Collaboration's tool for assessing quality of the included trials: Random sequence generation; Treatment allocation concealment; Blinding (participants and personnel); Blinding (outcome assessment); Incomplete outcome data; Selective reporting; Other bias. Each bias was classified into "high risk", "low risk" or "unclear risk".

Figure S2. Meta-analysis results of the effects of multicomponent integrated care on (A) all-cause mortality and (B) cardiovascular-related mortality among patients with acute coronary syndrome, stratified by median duration of follow-up

(A)

Study or Subgroup	Intervention Events	Intervention Total	Control Events	Control Total	Weight	Risk Ratio M-H, Fixed, 95% CI
**2.1.1 Follow-up time ≤12 months**						
Vaillant 2015	1	91	8	92	0.6%	0.13 [0.02, 0.99]
Briffa 2005	0	55	2	52	0.2%	0.19 [0.01, 3.85]
Goodman 2008	1	94	2	94	0.2%	0.50 [0.05, 5.42]
Ivers 2020	35	875	52	876	4.0%	0.67 [0.44, 1.02]
Jolly 1999	15	277	23	320	1.6%	0.75 [0.40, 1.42]
Capone 1988	33	504	43	500	3.3%	0.76 [0.49, 1.18]
Young 2003	8	71	11	75	0.8%	0.77 [0.33, 1.80]
Furze 2009	3	100	4	104	0.3%	0.78 [0.18, 3.40]
Berwanger 2012	42	602	46	547	3.7%	0.83 [0.56, 1.24]
Sinclair 2005	14	134	15	133	1.2%	0.93 [0.47, 1.84]
Wang 2019	237	6135	154	3967	14.3%	1.00 [0.82, 1.21]
Holmes-Rovner 2008	8	202	7	186	0.6%	1.05 [0.39, 2.85]
Ho 2014	11	122	9	119	0.7%	1.19 [0.51, 2.77]
Marcos-Forniol 2018	9	54	7	52	0.5%	1.24 [0.50, 3.08]
Wolf 2016	1	37	2	105	0.1%	1.42 [0.13, 15.19]
Yu 2020	3	496	2	496	0.2%	1.50 [0.25, 8.94]
**Subtotal (95% CI)**		**9849**		**7718**	**32.2%**	**0.88 [0.77, 1.01]**
Total events	421		387			
Heterogeneity: $Chi^2 = 10.21$, df = 15 (P = 0.81); $I^2 = 0\%$						
Test for overall effect: Z = 1.86 (P = 0.06)						
**2.1.2 Follow-up time >12 months**						
Yu 2004	3	132	5	72	0.5%	0.33 [0.08, 1.33]
Lin 2017	6	144	16	144	1.2%	0.38 [0.15, 0.93]
Lapointe 2006	2	64	4	63	0.3%	0.49 [0.09, 2.59]
Du 2016	25	479	49	485	3.7%	0.52 [0.32, 0.82]
Huffman 2018	445	11308	509	10066	41.3%	0.78 [0.69, 0.88]
Mayer-Berger 2014	12	211	19	272	1.3%	0.81 [0.40, 1.64]
Dracup 2009	67	1777	75	1745	5.8%	0.88 [0.64, 1.21]
Seidl 2015	29	161	32	168	2.4%	0.95 [0.60, 1.49]
Munoz, NLM 2007	139	515	129	468	10.4%	0.98 [0.80, 1.20]
Hanssen 2009	8	156	6	132	0.5%	1.13 [0.40, 3.17]
Fors 2017	6	94	5	105	0.4%	1.34 [0.42, 4.25]
Sunamura 2018	1	299	0	306	0.0%	3.07 [0.13, 75.06]
**Subtotal (95% CI)**		**15340**		**14026**	**67.8%**	**0.80 [0.73, 0.88]**
Total events	743		849			
Heterogeneity: $Chi^2 = 14.54$, df = 11 (P = 0.20); $I^2 = 24\%$						
Test for overall effect: Z = 4.54 (P < 0.00001)						
**Total (95% CI)**		**25189**		**21744**	**100.0%**	**0.83 [0.77, 0.90]**
Total events	1164		1236			
Heterogeneity: $Chi^2 = 25.77$, df = 27 (P = 0.53); $I^2 = 0\%$						
Test for overall effect: Z = 4.77 (P < 0.00001)						
Test for subgroup differences: $Chi^2 = 1.10$, df = 1 (P = 0.29), $I^2 = 9.5\%$						

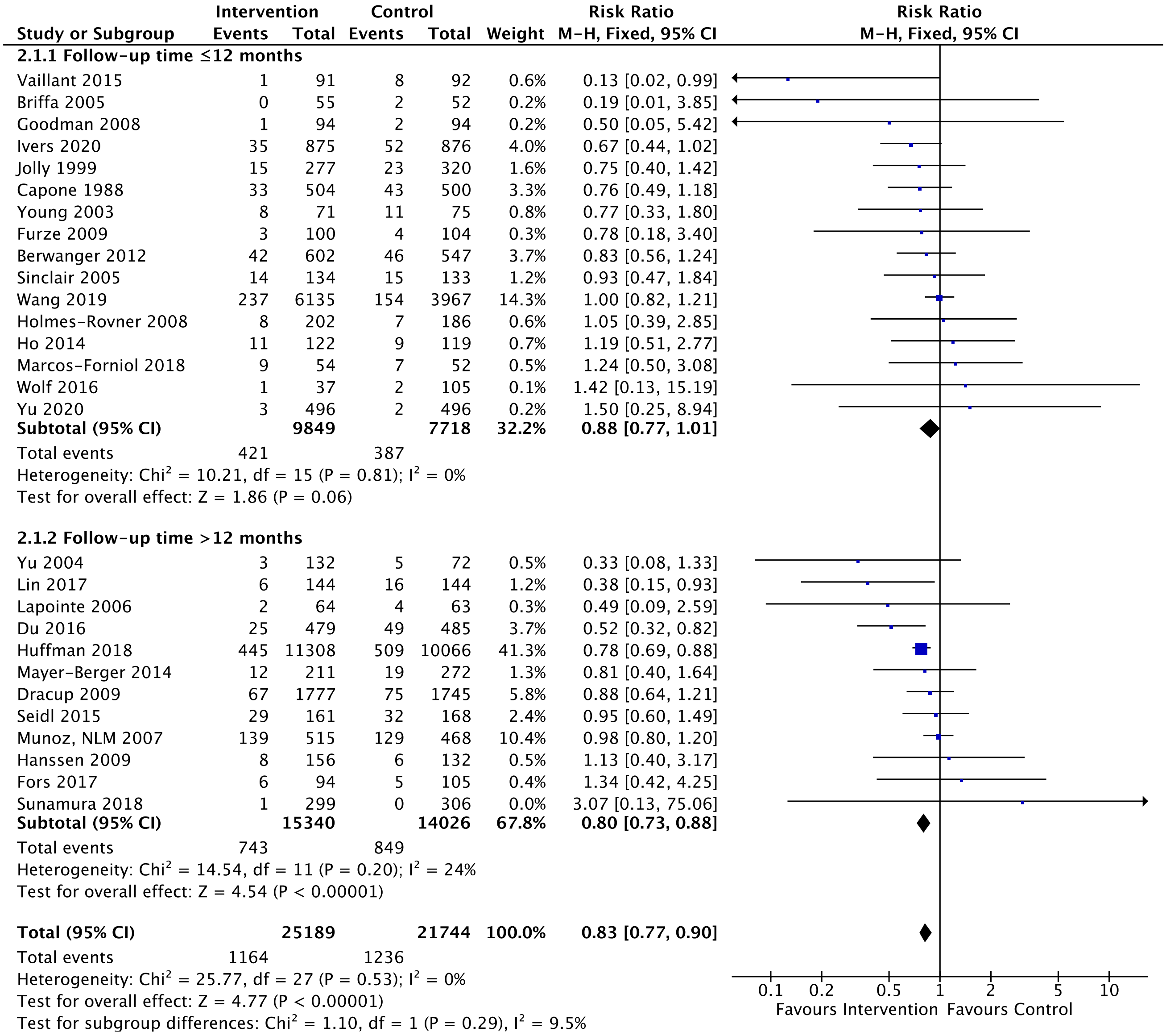

(B)

Study or Subgroup	Intervention Events	Intervention Total	Control Events	Control Total	Weight	Risk Ratio M-H, Fixed, 95% CI
**2.2.1 Follow-up time ≤12 months**						
Vaillant 2015	1	91	8	92	0.9%	0.13 [0.02, 0.99]
Wu Q 2019	0	75	1	75	0.2%	0.33 [0.01, 8.05]
Capone 1988	29	504	41	500	4.9%	0.70 [0.44, 1.11]
Chaves, GSS 2019	1	37	1	32	0.1%	0.86 [0.06, 13.28]
Berwanger 2012	40	602	39	547	4.9%	0.93 [0.61, 1.43]
Wang 2019	187	6135	117	3967	17.0%	1.03 [0.82, 1.30]
Furze 2009	1	100	1	104	0.1%	1.04 [0.07, 16.40]
**Subtotal (95% CI)**		**7544**		**5317**	**28.1%**	**0.92 [0.77, 1.10]**
Total events	259		208			
Heterogeneity: $Chi^2 = 6.31$, df = 6 (P = 0.39); $I^2 = 5\%$						
Test for overall effect: Z = 0.88 (P = 0.38)						
**2.2.2 Follow-up time >12 months**						
Du 2016	21	479	44	485	5.2%	0.48 [0.29, 0.80]
Mayer-Berger 2014	9	211	15	272	1.6%	0.77 [0.35, 1.73]
Huffman 2018	434	11308	494	10066	62.4%	0.78 [0.69, 0.89]
Munoz, NLM 2007	22	515	22	468	2.8%	0.91 [0.51, 1.62]
**Subtotal (95% CI)**		**12513**		**11291**	**71.9%**	**0.77 [0.68, 0.86]**
Total events	486		575			
Heterogeneity: $Chi^2 = 3.65$, df = 3 (P = 0.30); $I^2 = 18\%$						
Test for overall effect: Z = 4.44 (P < 0.00001)						
**Total (95% CI)**		**20057**		**16608**	**100.0%**	**0.81 [0.73, 0.89]**
Total events	745		783			
Heterogeneity: $Chi^2 = 13.15$, df = 10 (P = 0.22); $I^2 = 24\%$						
Test for overall effect: Z = 4.20 (P < 0.0001)						
Test for subgroup differences: $Chi^2 = 2.88$, df = 1 (P = 0.09), $I^2 = 65.2\%$						

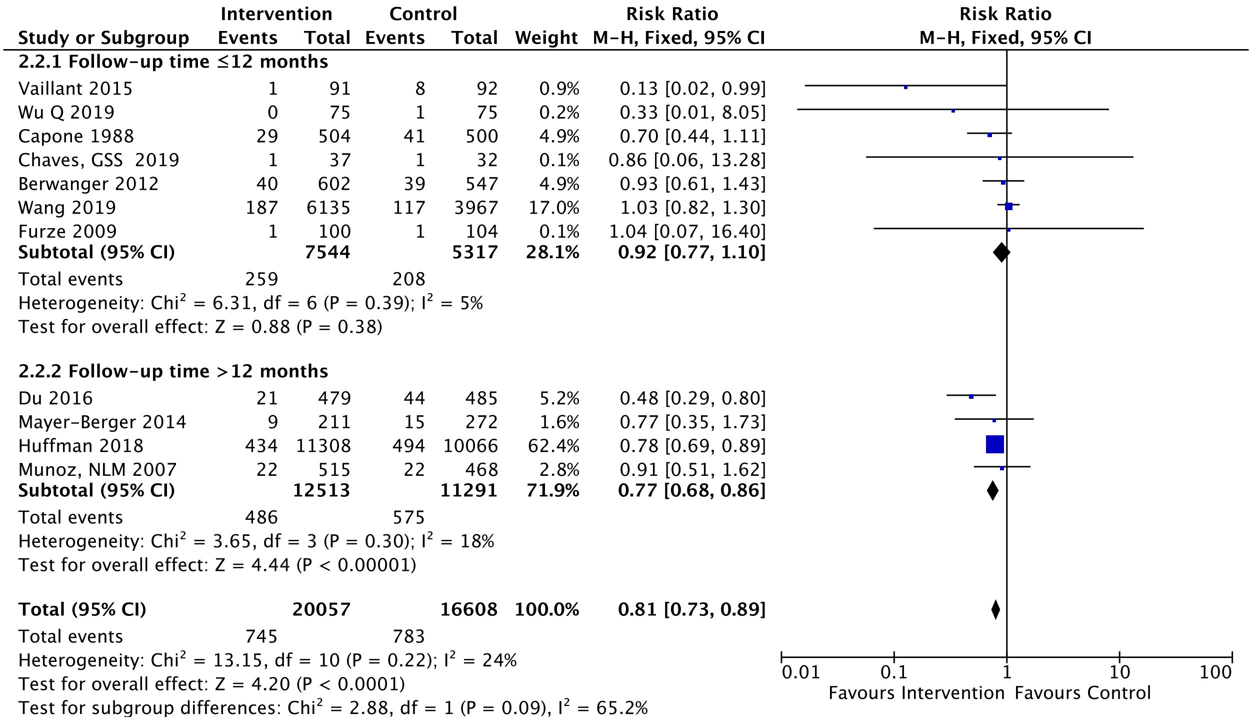

Footnotes: Forest plots were derived from fixed-effects meta-analysis models. M-H, Mantel-Haenszel method; 95% CI, 95% confidence interval.

Figure S3. Meta-analysis results of the effects of multicomponent integrated care on (A) all-cause mortality and (B) cardiovascular-related mortality among patients with acute coronary syndrome, after excluding trials conducted before 2010

(A)

Study or Subgroup	Intervention Events	Intervention Total	Control Events	Control Total	Weight	Risk Ratio M-H, Fixed, 95% CI
Vaillant 2015	1	91	8	92	0.8%	0.13 [0.02, 0.99]
Lin 2017	6	144	16	144	1.6%	0.38 [0.15, 0.93]
Du 2016	25	479	49	485	5.0%	0.52 [0.32, 0.82]
Ivers 2020	35	875	52	876	5.4%	0.67 [0.44, 1.02]
Huffman 2018	445	11308	509	10066	55.5%	0.78 [0.69, 0.88]
Mayer-Berger 2014	12	211	19	272	1.7%	0.81 [0.40, 1.64]
Berwanger 2012	42	602	46	547	5.0%	0.83 [0.56, 1.24]
Seidl 2015	29	161	32	168	3.2%	0.95 [0.60, 1.49]
Wang 2019	237	6135	154	3967	19.3%	1.00 [0.82, 1.21]
Ho 2014	11	122	9	119	0.9%	1.19 [0.51, 2.77]
Marcos-Forniol 2018	9	54	7	52	0.7%	1.24 [0.50, 3.08]
Fors 2017	6	94	5	105	0.5%	1.34 [0.42, 4.25]
Wolf 2016	1	37	2	105	0.1%	1.42 [0.13, 15.19]
Yu 2020	3	496	2	496	0.2%	1.50 [0.25, 8.94]
Sunamura 2018	1	299	0	306	0.1%	3.07 [0.13, 75.06]
**Total (95% CI)**		**21108**		**17800**	**100.0%**	**0.81 [0.74, 0.89]**
Total events	863		910			

Heterogeneity: $Chi^2 = 18.90$, df = 14 (P = 0.17); $I^2 = 26\%$
Test for overall effect: Z = 4.49 (P < 0.00001)

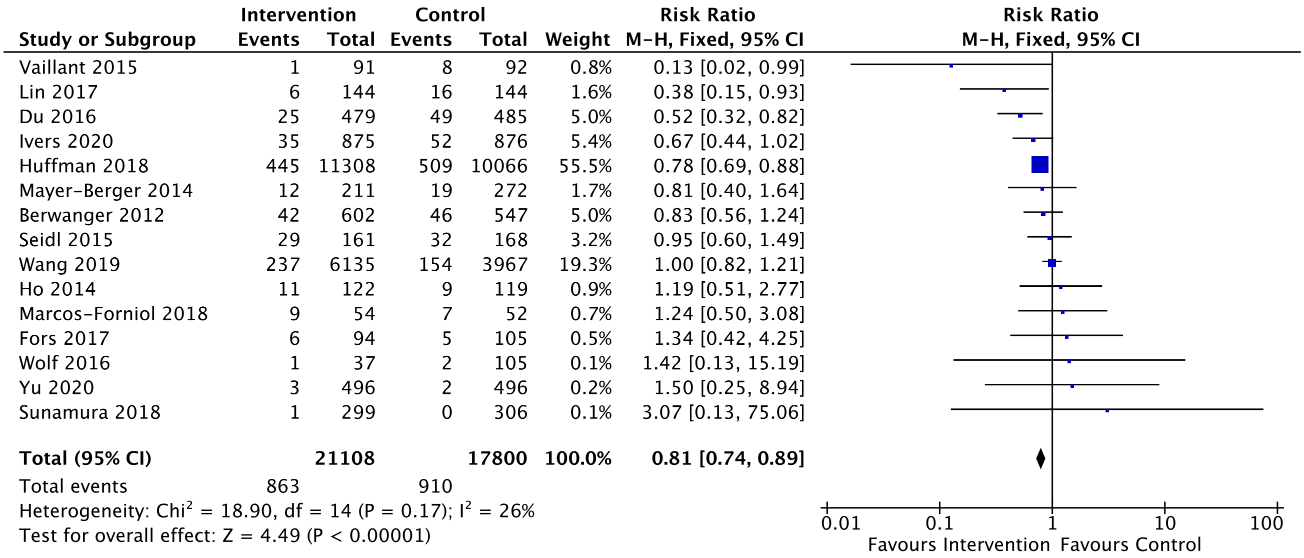

(B)

Study or Subgroup	Intervention Events	Intervention Total	Control Events	Control Total	Weight	Risk Ratio M-H, Fixed, 95% CI
Vaillant 2015	1	91	8	92	1.0%	0.13 [0.02, 0.99]
Wu Q 2019	0	75	1	75	0.2%	0.33 [0.01, 8.05]
Du 2016	21	479	44	485	5.7%	0.48 [0.29, 0.80]
Mayer-Berger 2014	9	211	15	272	1.7%	0.77 [0.35, 1.73]
Huffman 2018	434	11308	494	10066	67.6%	0.78 [0.69, 0.89]
Chaves, GSS 2019	1	37	1	32	0.1%	0.86 [0.06, 13.28]
Berwanger 2012	40	602	39	547	5.3%	0.93 [0.61, 1.43]
Wang 2019	187	6135	117	3967	18.4%	1.03 [0.82, 1.30]
**Total (95% CI)**		**18938**		**15536**	**100.0%**	**0.81 [0.73, 0.90]**
Total events	693		719			

Heterogeneity: $Chi^2 = 12.58$, df = 7 (P = 0.08); $I^2 = 44\%$
Test for overall effect: Z = 3.98 (P < 0.0001)

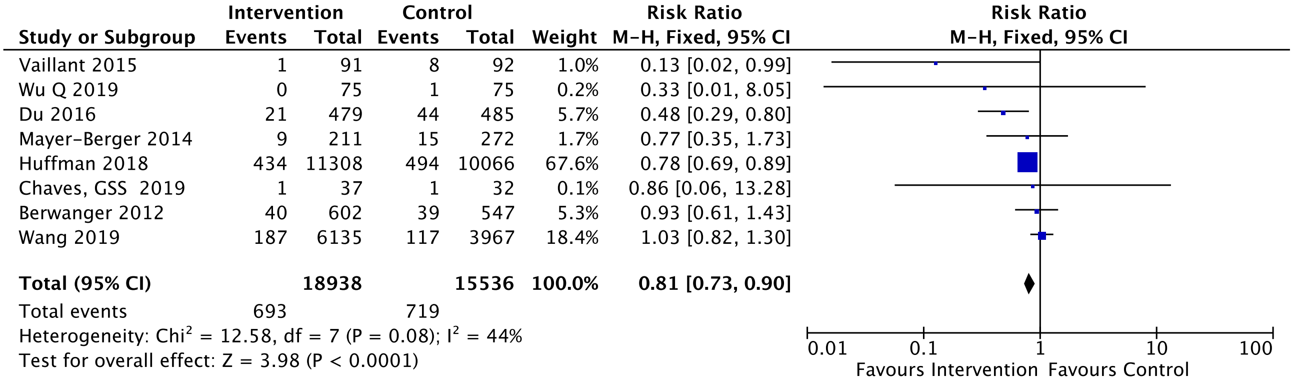

Footnotes: Forest plots were derived from fixed-effects meta-analysis models. M-H, Mantel-Haenszel method; 95% CI, 95% confidence interval.

Figure S4. Meta-analysis results of multicomponent integrated care on (A) all-cause hospitalization and (B) cardiovascular-related hospitalization among patients with acute coronary syndrome, stratified by median duration of follow-up

(A)

	Intervention		Control			Risk Ratio
**Study or Subgroup**	**Events**	**Total**	**Events**	**Total**	**Weight**	**M-H, Random, 95% CI**
**4.1.1 Follow-up time ≤12 months**						
Young 2003	1	71	9	75	0.4%	0.12 [0.02, 0.90]
Wu Q 2019	2	70	8	70	0.6%	0.25 [0.06, 1.14]
Furze 2009	1	100	3	104	0.3%	0.35 [0.04, 3.28]
Chaves, GSS 2019	3	37	8	39	0.9%	0.40 [0.11, 1.38]
Zhang 2018	9	100	17	99	2.2%	0.52 [0.25, 1.12]
Jorstad 2013	86	359	132	348	8.4%	0.63 [0.50, 0.79]
Sinclair 2005	35	163	51	161	5.7%	0.68 [0.47, 0.98]
Yu 2020	26	496	37	496	4.2%	0.70 [0.43, 1.14]
Briffa 2005	29	55	36	51	6.8%	0.75 [0.55, 1.01]
Jolly 1999	55	219	75	242	7.0%	0.81 [0.60, 1.09]
Heller 1993	47	213	60	237	6.3%	0.87 [0.62, 1.22]
Minneboo 2017	94	360	102	351	8.2%	0.90 [0.71, 1.14]
Wolf 2016	5	37	14	105	1.5%	1.01 [0.39, 2.62]
Ivers 2020	368	875	354	874	10.7%	1.04 [0.93, 1.16]
Tranmer 2004	9	92	8	92	1.6%	1.13 [0.45, 2.79]
Rollman 2009	85	150	68	152	8.4%	1.27 [1.01, 1.59]
**Subtotal (95% CI)**		**3397**		**3496**	**73.0%**	**0.81 [0.69, 0.95]**
Total events	855		982			
Heterogeneity: Tau² = 0.05; Chi² = 41.34, df = 15 (P = 0.0003); I² = 64%						
Test for overall effect: Z = 2.54 (P = 0.01)						
**4.1.2 Follow-up time >12 months**						
Mayer-Berger 2014	115	211	171	272	9.9%	0.87 [0.74, 1.01]
Fors 2017	22	94	25	105	4.0%	0.98 [0.60, 1.62]
Sunamura 2018	79	299	70	306	7.3%	1.15 [0.87, 1.53]
Yu 2004	34	132	16	72	3.8%	1.16 [0.69, 1.95]
Keeping-Burke 2013	14	84	8	84	1.9%	1.75 [0.78, 3.95]
**Subtotal (95% CI)**		**820**		**839**	**27.0%**	**1.03 [0.84, 1.26]**
Total events	264		290			
Heterogeneity: Tau² = 0.02; Chi² = 6.46, df = 4 (P = 0.17); I² = 38%						
Test for overall effect: Z = 0.24 (P = 0.81)						
**Total (95% CI)**		**4217**		**4335**	**100.0%**	**0.88 [0.78, 0.99]**
Total events	1119		1272			
Heterogeneity: Tau² = 0.03; Chi² = 47.24, df = 20 (P = 0.0005); I² = 58%						
Test for overall effect: Z = 2.08 (P = 0.04)						
Test for subgroup differences: Chi² = 3.13, df = 1 (P = 0.08), I² = 68.1%						

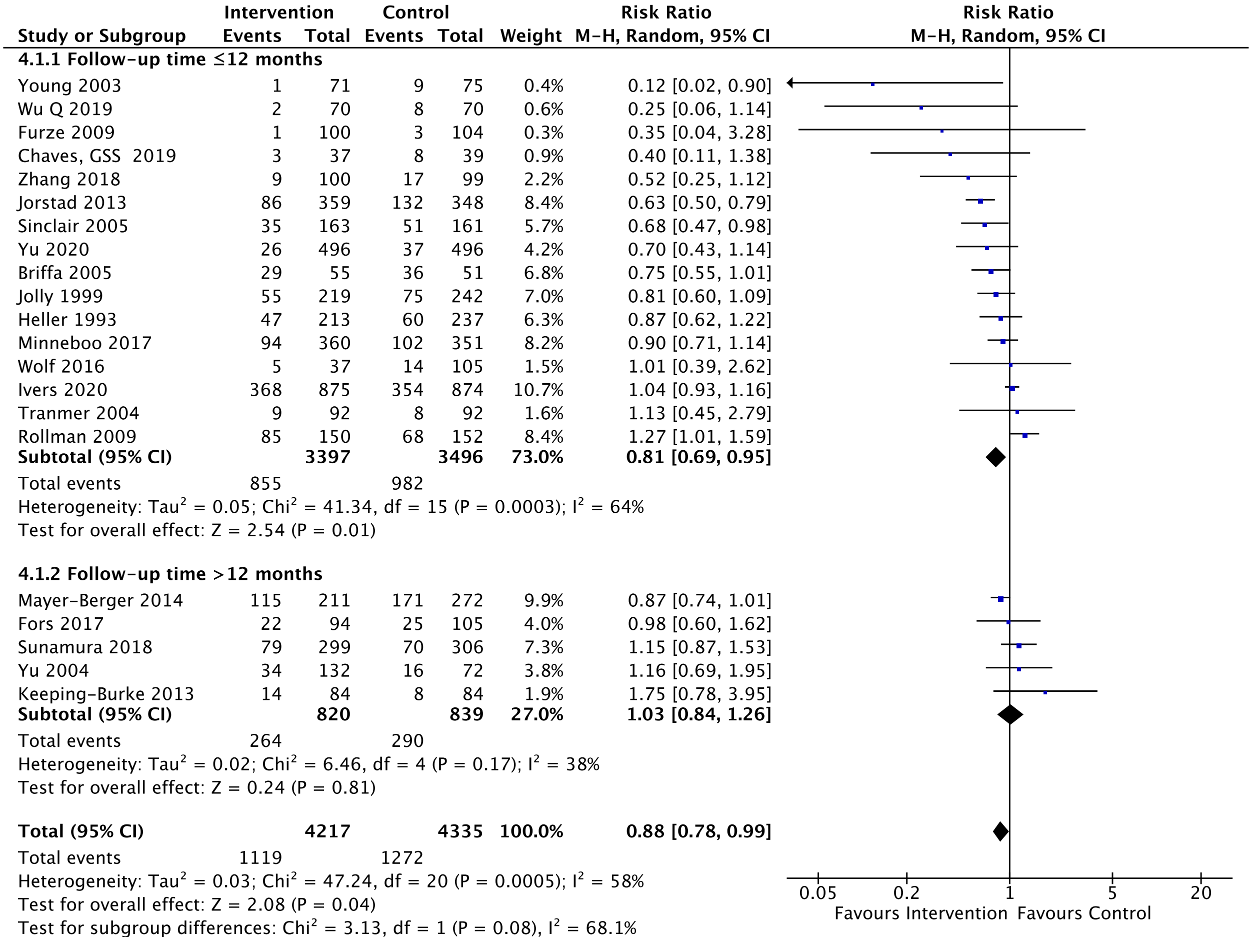

(B)

Study or Subgroup	Intervention Events	Intervention Total	Control Events	Control Total	Weight	Risk Ratio M-H, Random, 95% CI
**4.2.1 Follow-up time ≤12 months**						
Hautala 2017	5	109	16	95	1.8%	0.27 [0.10, 0.72]
Wu Q 2019	6	75	16	75	2.0%	0.38 [0.16, 0.91]
Yu 2020	4	496	7	496	1.2%	0.57 [0.17, 1.94]
Vaillant 2015	9	91	12	92	2.3%	0.76 [0.34, 1.71]
Jorstad 2013	17	361	21	349	3.3%	0.78 [0.42, 1.46]
Carroll 2007	24	121	31	126	4.5%	0.81 [0.50, 1.29]
Berwanger 2012	49	602	55	548	5.5%	0.81 [0.56, 1.17]
Ho 2014	22	122	26	119	4.1%	0.83 [0.50, 1.37]
Marcos-Forniol 2018	7	54	8	52	1.9%	0.84 [0.33, 2.16]
Rollman 2009	31	150	35	152	4.9%	0.90 [0.59, 1.38]
Ivers 2020	163	875	177	874	7.4%	0.92 [0.76, 1.11]
Wang 2019	614	6135	415	3967	8.0%	0.96 [0.85, 1.08]
Wang 2016	5	56	4	60	1.1%	1.34 [0.38, 4.74]
Katalinic 2008	263	752	127	748	7.4%	2.06 [1.71, 2.48]
Wang 2018	3	58	1	55	0.4%	2.84 [0.31, 26.53]
**Subtotal (95% CI)**		**10057**		**7808**	**55.8%**	**0.87 [0.68, 1.12]**
Total events	1222		951			
Heterogeneity: $Tau^2 = 0.14$; $Chi^2 = 75.44$, df = 14 (P < 0.00001); $I^2 = 81\%$						
Test for overall effect: Z = 1.05 (P = 0.29)						
**4.2.2 Follow-up time >12 months**						
Du 2016	88	479	137	485	6.9%	0.65 [0.51, 0.82]
Ma 2020	20	150	26	150	3.9%	0.77 [0.45, 1.32]
Huffman 2018	602	11308	645	10066	8.1%	0.83 [0.75, 0.93]
Mayer-Berger 2014	44	211	66	272	5.8%	0.86 [0.61, 1.20]
Wu Y 2019	559	14537	655	14809	8.0%	0.87 [0.78, 0.97]
Fors 2017	28	94	30	105	4.8%	1.04 [0.68, 1.61]
Munoz, NLM 2007	103	515	88	468	6.7%	1.06 [0.82, 1.37]
**Subtotal (95% CI)**		**27294**		**26355**	**44.2%**	**0.85 [0.77, 0.94]**
Total events	1444		1647			
Heterogeneity: $Tau^2 = 0.01$; $Chi^2 = 9.21$, df = 6 (P = 0.16); $I^2 = 35\%$						
Test for overall effect: Z = 3.22 (P = 0.001)						
**Total (95% CI)**		**37351**		**34163**	**100.0%**	**0.89 [0.77, 1.03]**
Total events	2666		2598			
Heterogeneity: $Tau^2 = 0.06$; $Chi^2 = 100.80$, df = 21 (P < 0.00001); $I^2 = 79\%$						
Test for overall effect: Z = 1.61 (P = 0.11)						
Test for subgroup differences: $Chi^2 = 0.05$, df = 1 (P = 0.83), $I^2 = 0\%$						

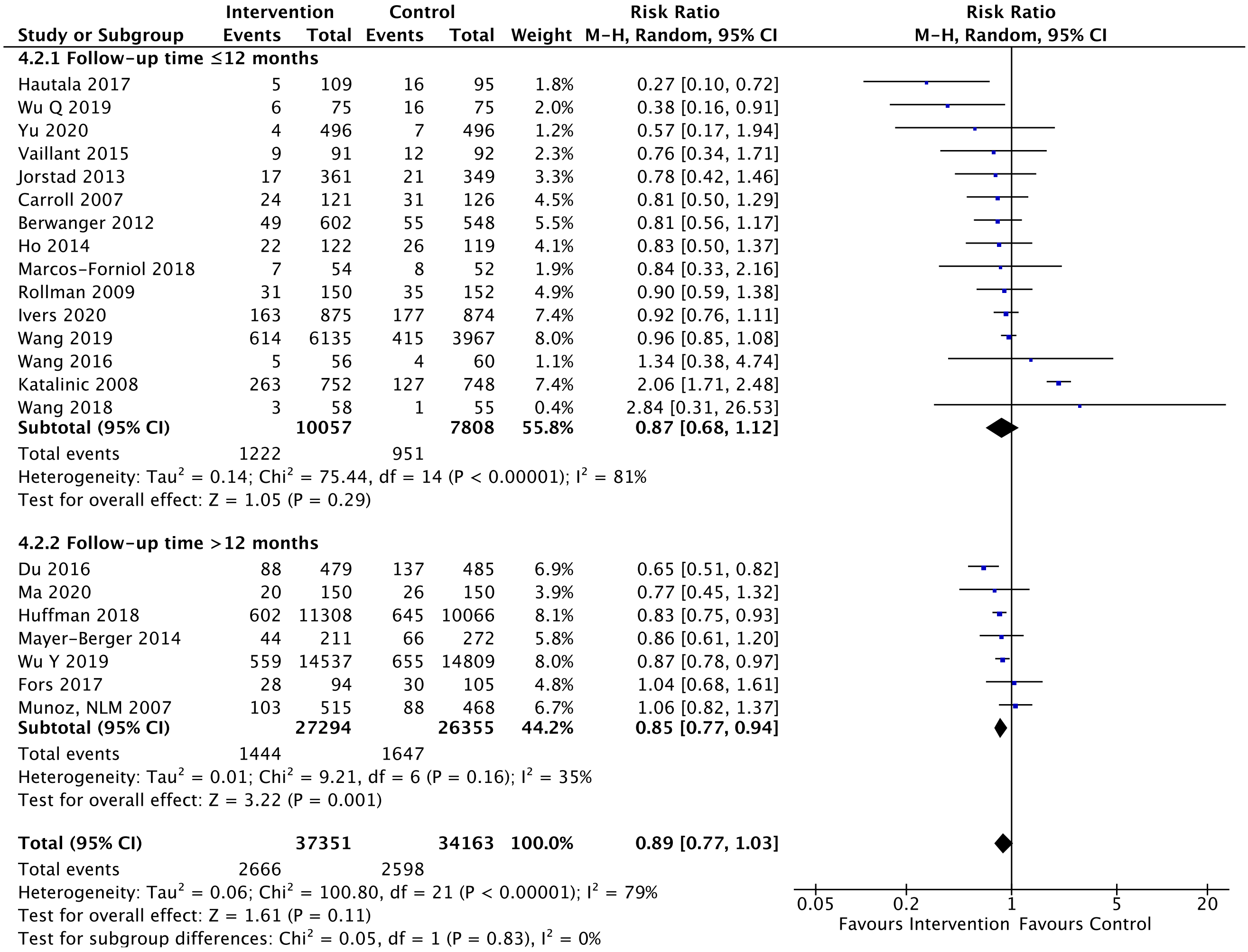

Footnotes: Forest plots were derived from random-effects meta-analysis models. M-H, Mantel-Haenszel method; 95% CI, 95% confidence interval.

Figure S5. Meta-analysis results of multicomponent integrated care on (A) all-cause hospitalization and (B) cardiovascular-related hospitalization among patients with acute coronary syndrome, after excluding trials conducted before 2010

(A)

Study or Subgroup	Intervention Events	Intervention Total	Control Events	Control Total	Weight	Risk Ratio M-H, Random, 95% CI
Wu Q 2019	2	70	8	70	1.0%	0.25 [0.06, 1.14]
Chaves, GSS 2019	3	37	8	39	1.5%	0.40 [0.11, 1.38]
Zhang 2018	9	100	17	99	3.6%	0.52 [0.25, 1.12]
Jorstad 2013	86	359	132	348	14.0%	0.63 [0.50, 0.79]
Yu 2020	26	496	37	496	6.9%	0.70 [0.43, 1.14]
Mayer-Berger 2014	115	211	171	272	16.7%	0.87 [0.74, 1.01]
Minneboo 2017	94	360	102	351	13.7%	0.90 [0.71, 1.14]
Fors 2017	22	94	25	105	6.7%	0.98 [0.60, 1.62]
Wolf 2016	5	37	14	105	2.5%	1.01 [0.39, 2.62]
Ivers 2020	368	875	354	874	18.0%	1.04 [0.93, 1.16]
Sunamura 2018	79	299	70	306	12.3%	1.15 [0.87, 1.53]
Keeping-Burke 2013	14	84	8	84	3.2%	1.75 [0.78, 3.95]
**Total (95% CI)**		**3022**		**3149**	**100.0%**	**0.87 [0.74, 1.02]**
Total events	823		946			

Heterogeneity: $Tau^2 = 0.03$; $Chi^2 = 28.58$, df = 11 (P = 0.003); $I^2 = 62\%$
Test for overall effect: Z = 1.69 (P = 0.09)

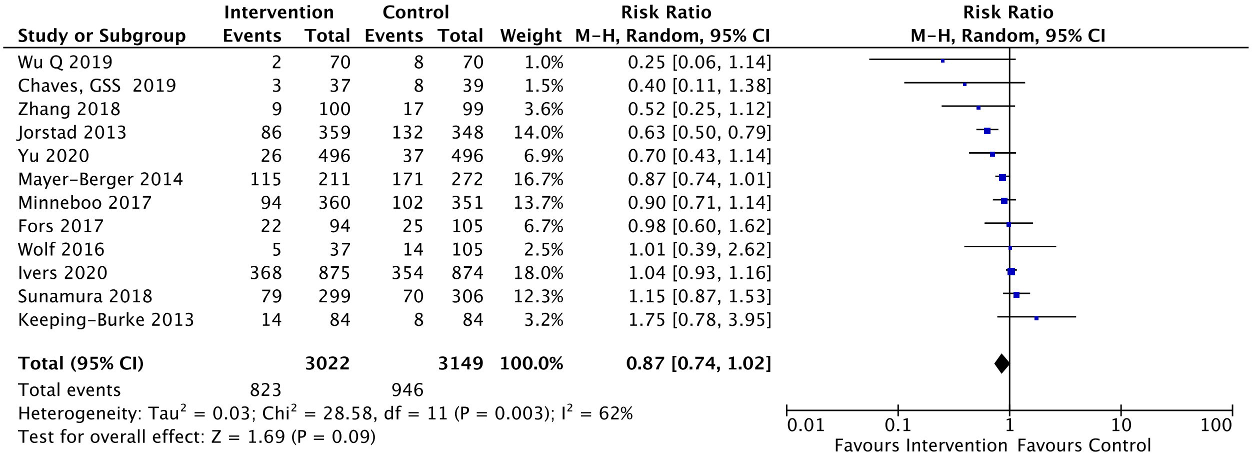

(B)

Study or Subgroup	Intervention Events	Intervention Total	Control Events	Control Total	Weight	Risk Ratio M-H, Fixed, 95% CI
Hautala 2017	5	109	16	95	0.7%	0.27 [0.10, 0.72]
Wu Q 2019	6	75	16	75	0.7%	0.38 [0.16, 0.91]
Yu 2020	4	496	7	496	0.3%	0.57 [0.17, 1.94]
Du 2016	88	479	137	485	5.6%	0.65 [0.51, 0.82]
Vaillant 2015	9	91	12	92	0.5%	0.76 [0.34, 1.71]
Ma 2020	20	150	26	150	1.1%	0.77 [0.45, 1.32]
Jorstad 2013	17	361	21	349	0.9%	0.78 [0.42, 1.46]
Berwanger 2012	49	602	55	548	2.4%	0.81 [0.56, 1.17]
Ho 2014	22	122	26	119	1.1%	0.83 [0.50, 1.37]
Huffman 2018	602	11308	645	10066	28.1%	0.83 [0.75, 0.93]
Marcos-Forniol 2018	7	54	8	52	0.3%	0.84 [0.33, 2.16]
Mayer-Berger 2014	44	211	66	272	2.4%	0.86 [0.61, 1.20]
Wu Y 2019	559	14537	655	14809	26.7%	0.87 [0.78, 0.97]
Ivers 2020	163	875	177	874	7.3%	0.92 [0.76, 1.11]
Wang 2019	614	6135	415	3967	20.7%	0.96 [0.85, 1.08]
Fors 2017	28	94	30	105	1.2%	1.04 [0.68, 1.61]
Wang 2016	5	56	4	60	0.2%	1.34 [0.38, 4.74]
Wang 2018	3	58	1	55	0.0%	2.84 [0.31, 26.53]
**Total (95% CI)**		**35813**		**32669**	**100.0%**	**0.86 [0.81, 0.91]**
Total events	2245		2317			

Heterogeneity: $Chi^2 = 21.50$, df = 17 (P = 0.20); $I^2 = 21\%$
Test for overall effect: Z = 5.36 (P < 0.00001)

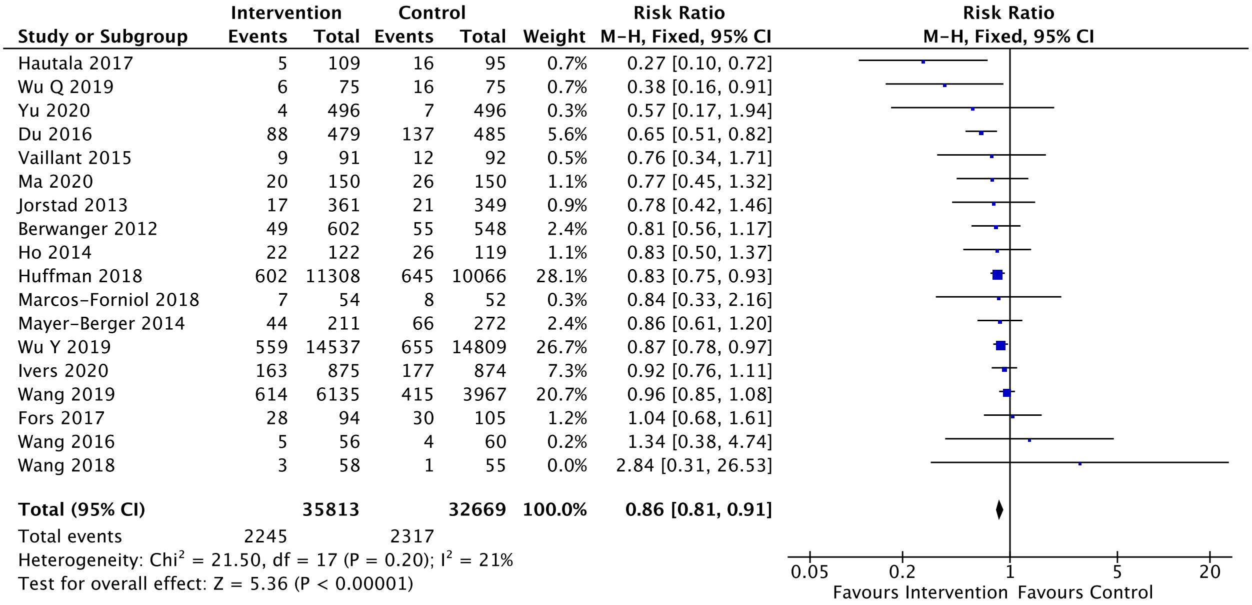

Footnotes: Forest plots were derived from random-effects (A) and fixed-effects (B) meta-analysis models. M-H, Mantel-Haenszel method; 95% CI, 95% confidence interval.

Figure S6. Meta-analysis results of the effects of multicomponent integrated care on emergency department visit

Study or Subgroup	Intervention Events	Intervention Total	Control Events	Control Total	Weight	Risk Ratio M-H, Random, 95% CI
Wang 2016	1	59	6	57	0.8%	0.16 [0.02, 1.30]
Chaves, GSS 2019	3	37	9	39	2.2%	0.35 [0.10, 1.20]
Young 2003	16	75	31	71	8.5%	0.49 [0.29, 0.81]
Jorstad 2013	29	361	51	349	10.1%	0.55 [0.36, 0.85]
Wang 2018	2	58	2	55	1.0%	0.95 [0.14, 6.50]
Dracup 2009	305	1580	260	1507	18.1%	1.12 [0.96, 1.30]
Ivers 2020	249	875	213	874	17.9%	1.17 [1.00, 1.37]
Capone 1988	192	504	157	500	17.6%	1.21 [1.02, 1.44]
Sunamura 2018	24	299	20	306	7.3%	1.23 [0.69, 2.18]
Keeping-Burke 2013	29	84	23	84	9.5%	1.26 [0.80, 1.99]
Tranmer 2004	21	92	15	92	6.9%	1.40 [0.77, 2.54]
**Total (95% CI)**		**4024**		**3934**	**100.0%**	**0.98 [0.81, 1.19]**
Total events	871		787			

Heterogeneity: $Tau^2 = 0.05$; $Chi^2 = 29.43$, df = 10 (P = 0.001); $I^2 = 66\%$
Test for overall effect: Z = 0.17 (P = 0.86)

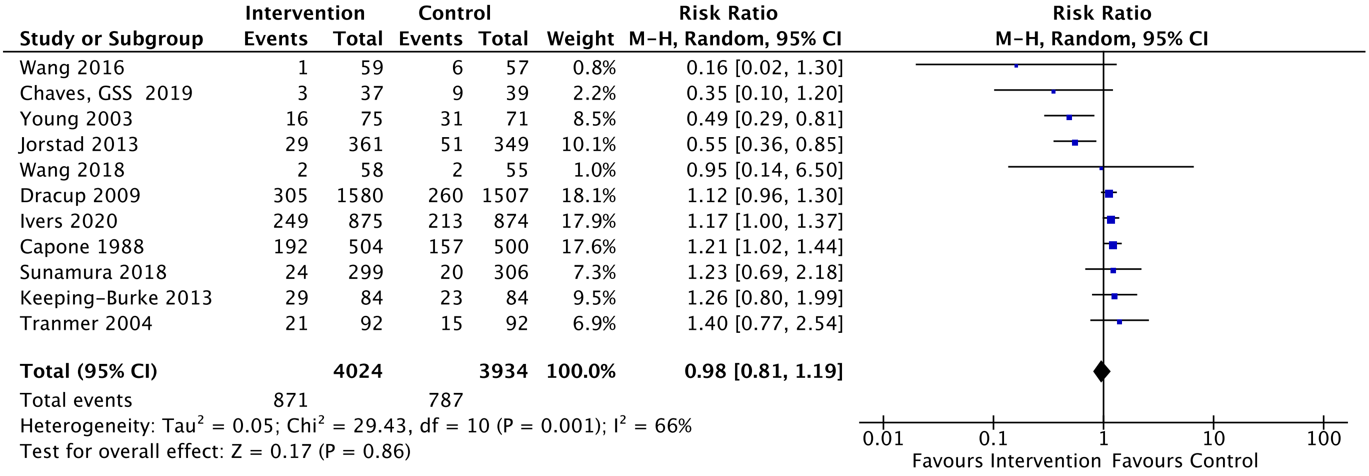

Footnotes: Forest plot was derived from a random-effects meta-analysis model. M-H, Mantel-Haenszel method; SE, standard error; 95% CI, 95% confidence interval.

Figure S7. Meta-analysis results of the effects of multicomponent integrated care on unplanned outpatient visit

Study or Subgroup	Intervention Events	Intervention Total	Control Events	Control Total	Weight	Risk Ratio M-H, Fixed, 95% CI
Keeping-Burke 2013	30	82	43	82	4.5%	0.70 [0.49, 0.99]
Wang 2016	8	56	11	60	1.1%	0.78 [0.34, 1.80]
Wang 2018	2	58	2	55	0.2%	0.95 [0.14, 6.50]
Sinclair 2005	92	134	94	133	10.0%	0.97 [0.83, 1.14]
Ivers 2020	754	875	731	874	77.2%	1.03 [0.99, 1.07]
Jolly 1999	1	219	1	242	0.1%	1.11 [0.07, 17.56]
Tranmer 2004	9	92	8	92	0.8%	1.13 [0.45, 2.79]
Furze 2009	25	100	22	104	2.3%	1.18 [0.71, 1.95]
Zhao 2009	57	100	36	100	3.8%	1.58 [1.16, 2.16]
**Total (95% CI)**		**1716**		**1742**	**100.0%**	**1.03 [0.99, 1.08]**
Total events	978		948			

Heterogeneity: $Chi^2 = 13.32$, df = 8 (P = 0.10); $I^2 = 40\%$
Test for overall effect: Z = 1.42 (P = 0.16)

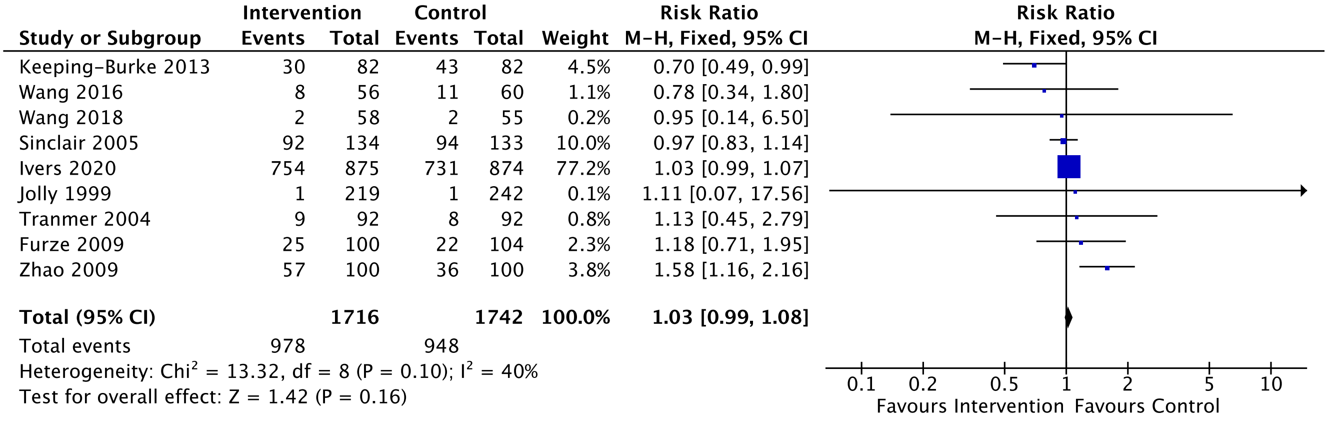

Footnotes: Forest plot was derived from a fixed-effects meta-analysis model. M-H, Mantel-Haenszel method; SE, standard error; 95% CI, 95% confidence interval.

Figure S8. Meta-analysis results of changes of medications namely (A) antiplatelet/antithrombotic therapy, (B) renin-angiotensin-aldosterone system (RAAS) inhibitors and (C) lipid-lowering drugs from baseline to end of intervention.

(A) Antiplatelet or antithrombotic therapy

Study or Subgroup	log[Risk Ratio]	SE	Weight	Risk Ratio IV, Random, 95% CI
Dalal 2007	−0.1863	0.1093	4.7%	0.83 [0.67, 1.03]
Minneboo 2017	−0.0619	0.011	7.2%	0.94 [0.92, 0.96]
Yu 2020	−0.0513	0.0164	7.2%	0.95 [0.92, 0.98]
Jorstad 2013	−0.0101	0.0157	7.2%	0.99 [0.96, 1.02]
Mayer-Berger 2014	−0.0101	0.0157	7.2%	0.99 [0.96, 1.02]
Mosca 2010	0	0.0481	6.6%	1.00 [0.91, 1.10]
Lear 2003	0	0.0425	6.7%	1.00 [0.92, 1.09]
Huffman 2018	0	0.0051	7.3%	1.00 [0.99, 1.01]
Medman 2007	0.01	0.0206	7.1%	1.01 [0.97, 1.05]
Redfern 2009	0.01	0.0703	6.0%	1.01 [0.88, 1.16]
Williams 2011	0.0392	0.02	7.1%	1.04 [1.00, 1.08]
Munoz, NLM 2007	0.1133	0.0477	6.6%	1.12 [1.02, 1.23]
Ivers 2020	0.239	0.0418	6.7%	1.27 [1.17, 1.38]
Berwanger 2012	1.0682	0.0439	6.7%	2.91 [2.67, 3.17]
Wang 2019	1.3584	0.0808	5.6%	3.89 [3.32, 4.56]
**Total (95% CI)**			**100.0%**	**1.17 [1.08, 1.27]**

Heterogeneity: Tau^2 = 0.02; Chi^2 = 961.21, df = 14 (P < 0.00001); I^2 = 99%
Test for overall effect: Z = 3.89 (P < 0.0001)

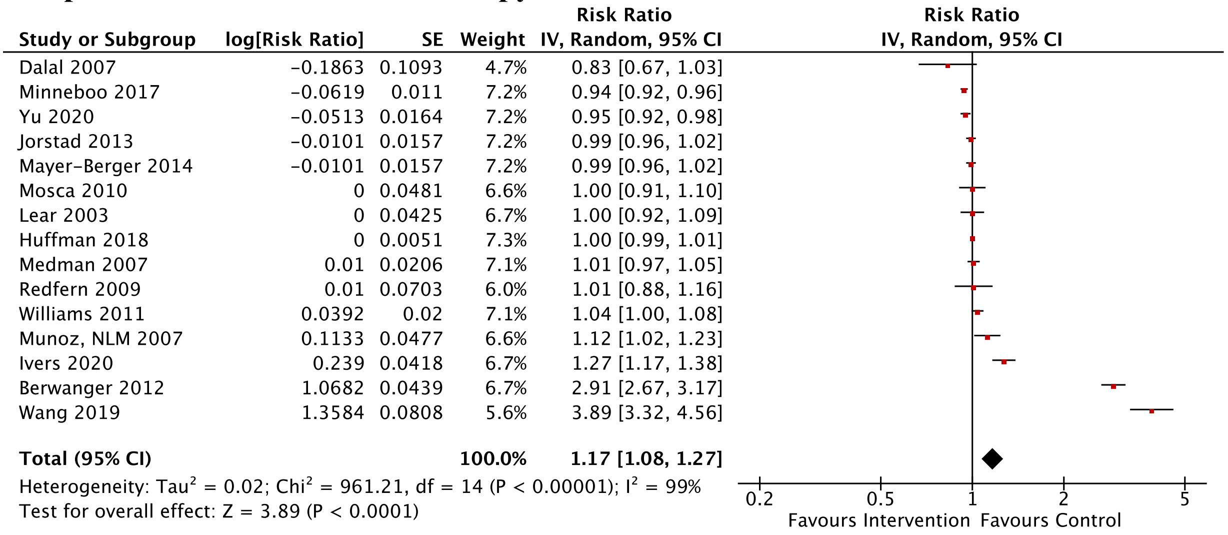

(B) RAAS inhibitor

Study or Subgroup	log[Risk Ratio]	SE	Weight	Risk Ratio IV, Random, 95% CI
Minneboo 2017	−0.2231	0.0329	10.3%	0.80 [0.75, 0.85]
Mayer-Berger 2014	−0.0834	0.0344	10.3%	0.92 [0.86, 0.98]
Redfern 2009	0.0583	0.095	8.1%	1.06 [0.88, 1.28]
Ivers 2020	0.0677	0.0294	10.4%	1.07 [1.01, 1.13]
Jorstad 2013	0.1133	0.0427	10.0%	1.12 [1.03, 1.22]
Mosca 2010	0.1655	0.0547	9.7%	1.18 [1.06, 1.31]
Dalal 2007	0.174	0.1149	7.3%	1.19 [0.95, 1.49]
Williams 2011	0.2151	0.0752	8.9%	1.24 [1.07, 1.44]
Sturchio 2012	0.2311	0.2318	3.7%	1.26 [0.80, 1.98]
Lear 2003	0.2624	0.0852	8.5%	1.30 [1.10, 1.54]
Munoz, NLM 2007	0.3075	0.0768	8.9%	1.36 [1.17, 1.58]
Yu 2020	0.7467	0.2279	3.8%	2.11 [1.35, 3.30]
**Total (95% CI)**			**100.0%**	**1.13 [1.02, 1.27]**

Heterogeneity: Tau^2 = 0.03; Chi^2 = 119.90, df = 11 (P < 0.00001); I^2 = 91%
Test for overall effect: Z = 2.27 (P = 0.02)

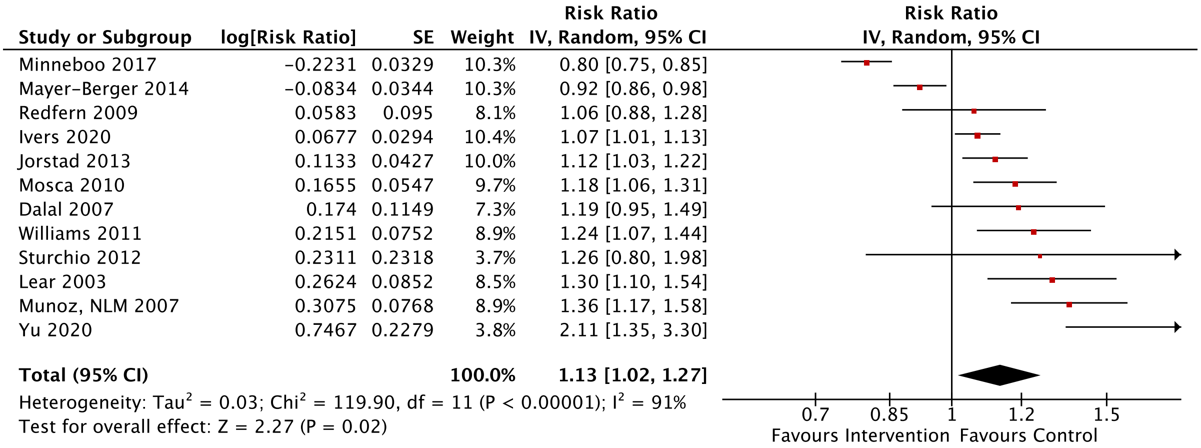

(C) Lipid-lowering drugs

Study or Subgroup	log[Risk Ratio]	SE	Weight	Risk Ratio IV, Random, 95% CI
Yu 2020	−0.1863	0.0252	8.4%	0.83 [0.79, 0.87]
Jorstad 2013	−0.0305	0.0106	8.6%	0.97 [0.95, 0.99]
Medman 2007	−0.0202	0.0322	8.3%	0.98 [0.92, 1.04]
Mayer-Berger 2014	0	0.0262	8.4%	1.00 [0.95, 1.05]
Lear 2003	0.01	0.0366	8.2%	1.01 [0.94, 1.09]
Ivers 2020	0.0488	0.0565	7.6%	1.05 [0.94, 1.17]
Dalal 2007	0.1655	0.0547	7.7%	1.18 [1.06, 1.31]
Redfern 2009	0.174	0.0687	7.2%	1.19 [1.04, 1.36]
Williams 2011	0.174	0.0309	8.3%	1.19 [1.12, 1.26]
Minneboo 2017	0.2311	0.0292	8.3%	1.26 [1.19, 1.33]
Lapointe 2006	0.3221	0.0629	7.4%	1.38 [1.22, 1.56]
Munoz, NLM 2007	0.3716	0.0365	8.2%	1.45 [1.35, 1.56]
Sturchio 2012	0.5423	0.1923	3.3%	1.72 [1.18, 2.51]
Carlsson 1998	1.411	0.9978	0.2%	4.10 [0.58, 28.98]
**Total (95% CI)**			**100.0%**	**1.13 [1.03, 1.23]**

Heterogeneity: Tau^2 = 0.02; Chi^2 = 306.57, df = 13 (P < 0.00001); I^2 = 96%
Test for overall effect: Z = 2.65 (P = 0.008)

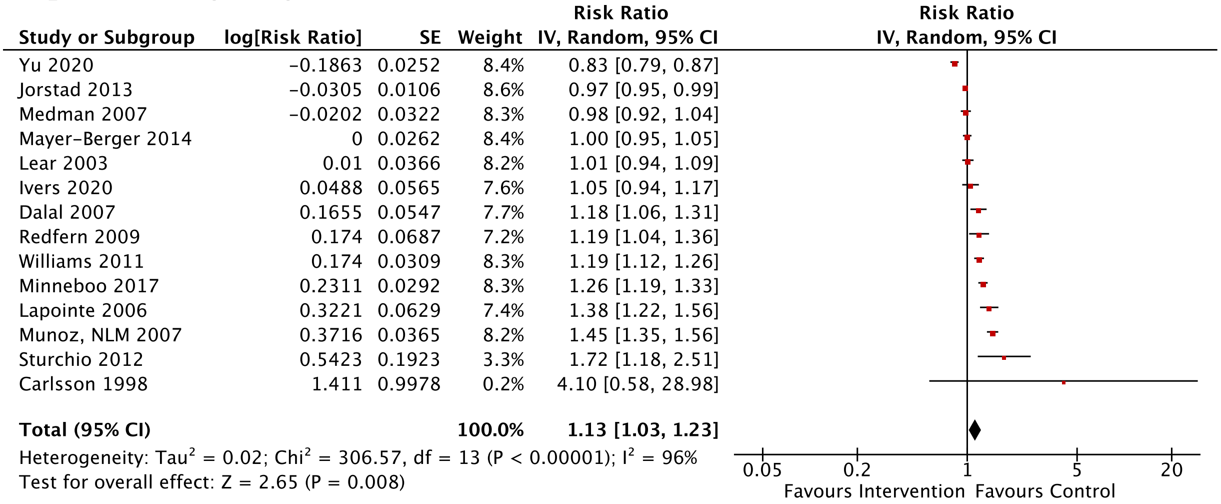

Footnotes: Forest plots were derived from random-effects meta-analysis models. SE, standard error; 95% CI, 95% confidence interval.

Figure S9. Meta-analysis results of changes of cardiometabolic risk factors namely (A) fasting plasma glucose (FPG), (B) systolic blood pressure (SBP), (C) diastolic blood pressure (DBP), (D) total cholesterol, (E) low-density lipoprotein (LDL) cholesterol, (F) triglyceride and (G) body mass index (BMI), from baseline to the end of intervention.

(A) FPG

Study or Subgroup	Experimental Mean	Experimental SD	Experimental Total	Control Mean	Control SD	Control Total	Weight	Mean Difference IV, Fixed, 95% CI
Wang 2016	−0.55	3.5005	128	0.49	2.8839	128	8.3%	−1.04 [−1.83, −0.25]
Du 2016	−0.5	5.5713	958	−0.1	5.3849	970	21.3%	−0.40 [−0.89, 0.09]
Skobel 2017	−0.27	3.3644	74	−0.12	4.1531	105	4.2%	−0.15 [−1.25, 0.95]
Maddison 2019	0.43	3.5101	150	0.58	3.9982	152	7.1%	−0.15 [−1.00, 0.70]
Leemrijse 2016	0.1	2.3625	300	0.16	3.3663	338	25.4%	−0.06 [−0.51, 0.39]
Munoz, NLM 2007	−0.05	3.4814	893	0	11.0233	808	8.1%	−0.05 [−0.84, 0.74]
Lear 2003	0.2	2.5177	284	0.2	2.8285	272	25.6%	0.00 [−0.45, 0.45]
**Total (95% CI)**			**2787**			**2773**	**100.0%**	**−0.21 [−0.43, 0.02]**

Heterogeneity: $Chi^2 = 6.34$, df = 6 (P = 0.39); $I^2 = 5\%$

Test for overall effect: Z = 1.80 (P = 0.07)

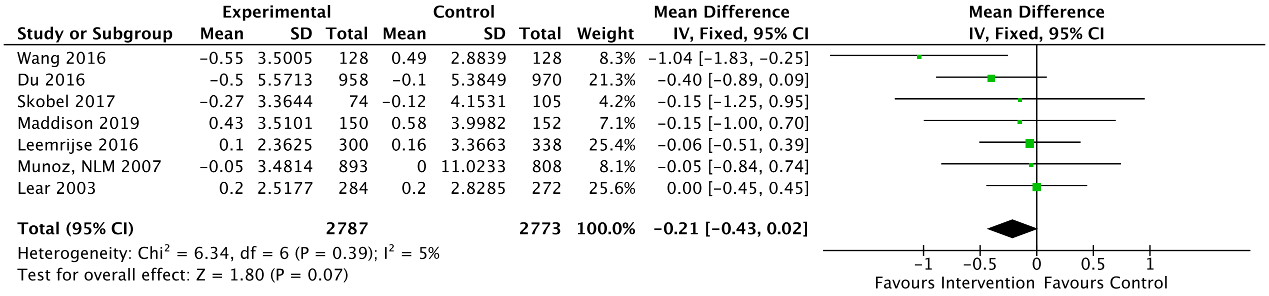

(B) SBP

Study or Subgroup	Experimental Mean	Experimental SD	Experimental Total	Control Mean	Control SD	Control Total	Weight	Mean Difference IV, Random, 95% CI
Redfern 2009	−5	3.91	139	6.4	4.48	141	5.6%	−11.40 [−12.38, −10.42]
Zhang 2017	−14.1	29.73	200	−4.4	28.5	198	4.7%	−9.70 [−15.42, −3.98]
Chaves, GSS 2019	−4.6	37.9	63	5	40.05	69	2.7%	−9.60 [−22.90, 3.70]
Ho 2014	−12	36.18	244	−3	42.03	238	4.4%	−9.00 [−16.01, −1.99]
Sturchio 2012	0	36.04	168	7	67.52	168	3.1%	−7.00 [−18.57, 4.57]
Alsaleh 2016	−6.12	32.01	132	0.57	32.51	158	4.2%	−6.69 [−14.14, 0.76]
Vaillant 2015	−13	26.81	70	−7	28.11	55	3.6%	−6.00 [−15.73, 3.73]
Vale 2003	0.1	54.41	796	4.5	62.59	788	4.7%	−4.40 [−10.18, 1.38]
Goodman 2008	−13.03	43.26	180	−9.11	39.17	186	3.9%	−3.92 [−12.38, 4.54]
Varnfield 2014	−2.8	33.62	92	0.4	29.4	52	3.4%	−3.20 [−13.74, 7.34]
Santo 2019	−0.9	34.36	208	1.6	39.32	107	3.8%	−2.50 [−11.29, 6.29]
Dalal 2007	10.3	47.84	120	12.8	46.52	88	2.8%	−2.50 [−15.45, 10.45]
Lear 2003	1	45.14	284	3	42.07	272	4.3%	−2.00 [−9.25, 5.25]
Maddison 2019	−3.6	35.99	150	−1.7	34.56	149	4.1%	−1.90 [−9.90, 6.10]
Wu Q 2019	−4.34	32.42	140	−3.01	25.41	140	4.4%	−1.33 [−8.15, 5.49]
Jolly 1999	8.9	40.52	512	10.1	42.28	587	4.9%	−1.20 [−6.10, 3.70]
Du 2016	−8.19	42.32	958	−8.19	50.69	970	5.1%	0.00 [−4.17, 4.17]
Leemrijse 2016	5	35.19	316	5	39.2	346	4.7%	0.00 [−5.67, 5.67]
Yu 2020	−0.02	26.86	994	−0.3	26.21	993	5.5%	0.28 [−2.05, 2.61]
Johnston 2016	−0.6	28.92	166	−1.1	35.41	152	4.3%	0.50 [−6.64, 7.64]
Munoz, NLM 2007	−1.1	39.79	893	−2	61.06	808	4.9%	0.90 [−4.05, 5.85]
Wang 2016	11.58	38.67	128	8.19	43.23	128	3.5%	3.39 [−6.66, 13.44]
Chair 2013	1.5	34.45	125	−5.6	36.01	137	3.9%	7.10 [−1.43, 15.63]
Skobel 2017	9.26	36.91	74	−3.67	36.18	105	3.3%	12.93 [2.04, 23.82]
**Total (95% CI)**			**7152**			**7035**	**100.0%**	**−2.48 [−5.53, 0.57]**

Heterogeneity: $Tau^2 = 42.92$; $Chi^2 = 186.04$, df = 23 (P < 0.00001); $I^2 = 88\%$

Test for overall effect: Z = 1.59 (P = 0.11)

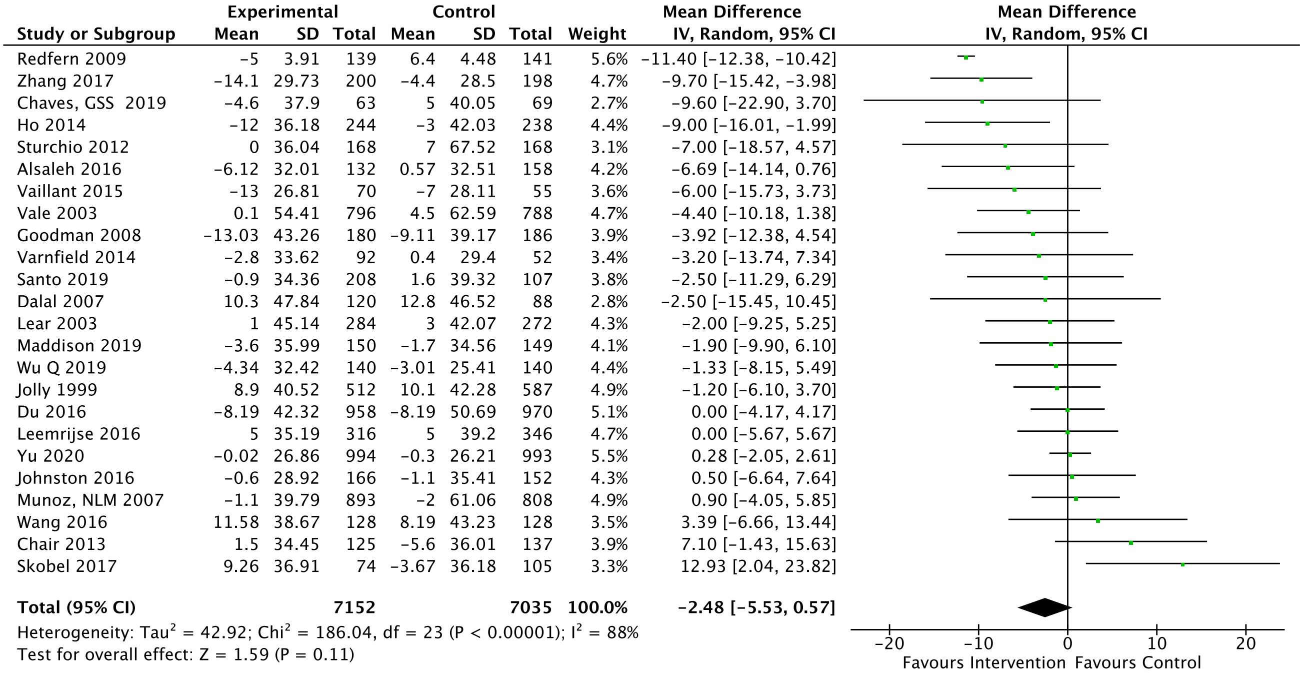

(C)DBP

Study or Subgroup	Experimental Mean	Experimental SD	Experimental Total	Control Mean	Control SD	Control Total	Weight	Mean Difference IV, Random, 95% CI
Vaillant 2015	−9	24.8	70	2	29.43	66	2.0%	−11.00 [−20.17, −1.83]
Zhang 2017	−7.6	27.8	218	−0.7	15.41	217	5.2%	−6.90 [−11.12, −2.68]
Alsaleh 2016	−1.79	19.7	132	4.8	16.74	158	5.2%	−6.59 [−10.85, −2.33]
Redfern 2009	−0.3	2.53	139	5.07	14.6	141	7.4%	−5.37 [−7.82, −2.92]
Varnfield 2014	−2.5	17.62	92	1.4	17.22	52	3.7%	−3.90 [−9.81, 2.01]
Wu Q 2019	−3.18	19.8	140	0	24.39	140	4.2%	−3.18 [−8.38, 2.02]
Goodman 2008	−7.96	22.25	180	−5.38	22.82	186	4.8%	−2.58 [−7.20, 2.04]
Vale 2003	0.4	35.6	773	2.8	38.02	773	5.9%	−2.40 [−6.07, 1.27]
Santo 2019	−0.1	19.28	208	2.2	21.9	107	4.5%	−2.30 [−7.21, 2.61]
Ho 2014	−5	23.99	244	−3	30.62	238	4.5%	−2.00 [−6.92, 2.92]
Chaves, GSS 2019	0.1	24.46	70	2	32.89	69	1.8%	−1.90 [−11.55, 7.75]
Jolly 1999	2.7	26.21	512	4	27.19	587	6.5%	−1.30 [−4.46, 1.86]
Jorstad 2013	1.9	22.53	734	3	22.7	727	7.6%	−1.10 [−3.42, 1.22]
Lear 2003	0	23.06	274	1	20.05	282	5.9%	−1.00 [−4.60, 2.60]
Maddison 2019	−1.72	21.47	147	−2.07	21.05	149	4.6%	0.35 [−4.49, 5.19]
Yu 2020	4.72	17.37	994	4.25	17.04	993	8.5%	0.47 [−1.04, 1.98]
Chair 2013	−2.4	21.39	125	−3.8	21.98	137	4.2%	1.40 [−3.85, 6.65]
Wang 2016	2.9	18.01	128	0.63	23.55	128	4.3%	2.27 [−2.87, 7.41]
Munoz, NLM 2007	−0.7	24.7	893	−3.5	100.36	808	2.9%	2.80 [−4.31, 9.91]
Dalal 2007	11.3	26.27	120	7.7	28.62	88	2.6%	3.60 [−4.01, 11.21]
Skobel 2017	4.09	19.05	74	−3.35	19.66	105	3.8%	7.44 [1.70, 13.18]
**Total (95% CI)**			**6267**			**6151**	**100.0%**	**−1.64 [−3.09, −0.20]**

Heterogeneity: $Tau^2 = 5.73$; $Chi^2 = 50.07$, df = 20 (P = 0.0002); $I^2 = 60\%$
Test for overall effect: Z = 2.24 (P = 0.03)

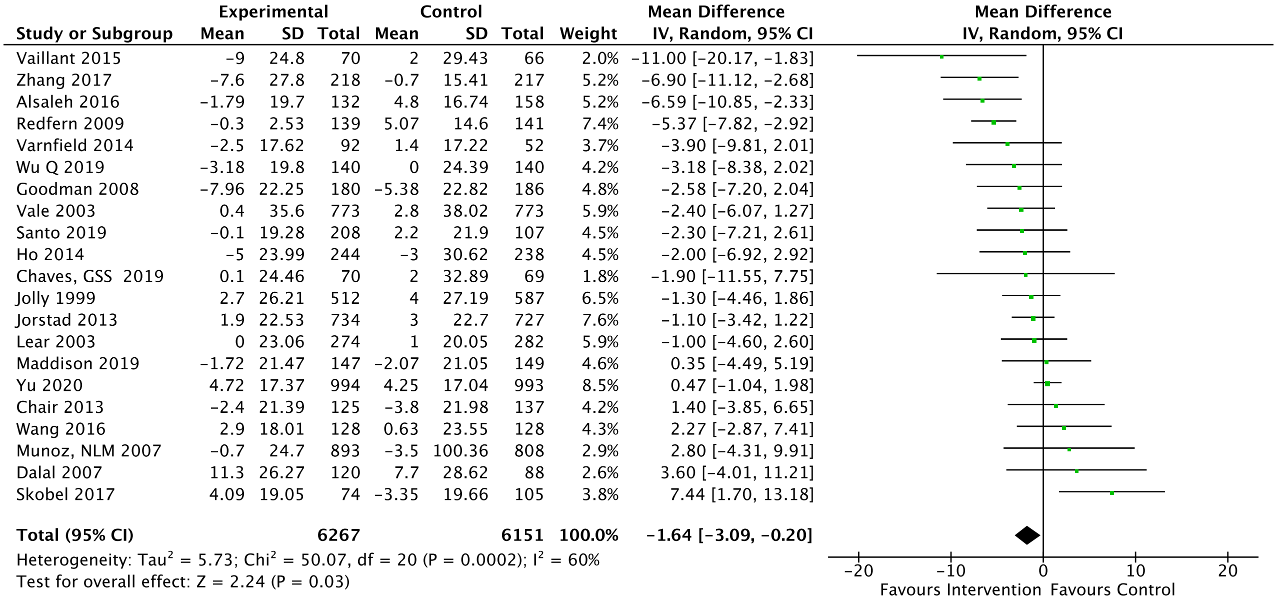

(D)Total cholesterol

Study or Subgroup	Intervention Mean	Intervention SD	Intervention Total	Control Mean	Control SD	Control Total	Weight	Mean Difference IV, Random, 95% CI
Sturchio 2012	−0.72	2.38	168	0.73	2.31	168	4.6%	−1.45 [−1.95, −0.95]
Redfern 2009	−0.8	0.18	139	0.19	1.27	141	6.1%	−0.99 [−1.20, −0.78]
Zhang 2017	0.1	2.09	199	1.01	2.99	598	5.3%	−0.91 [−1.29, −0.53]
Wu Q 2019	−0.95	2.23	140	−0.46	1.99	140	4.6%	−0.49 [−0.99, 0.01]
Vale 2003	−0.54	2.3	796	−0.18	6.87	788	4.6%	−0.36 [−0.87, 0.15]
Skobel 2017	−0.19	2.28	74	0.11	1.75	105	4.0%	−0.30 [−0.92, 0.32]
Lin 2017	−0.28	1.82	288	−0.06	1.9	288	5.7%	−0.22 [−0.52, 0.08]
Dalal 2007	−1.12	2.35	120	−0.93	2.25	88	3.9%	−0.19 [−0.82, 0.44]
Jolly 1999	−0.3	2.66	512	−0.17	2.6	587	5.6%	−0.13 [−0.44, 0.18]
Leemrijse 2016	0.1	1.68	302	0.22	1.78	338	5.8%	−0.12 [−0.39, 0.15]
Munoz, NLM 2007	−0.38	2.52	893	−0.29	3.05	808	5.9%	−0.09 [−0.36, 0.18]
Chair 2013	−0.8	1.83	125	−0.74	1.97	137	4.8%	−0.06 [−0.52, 0.40]
Lear 2003	−0.1	1.81	284	−0.05	1.77	272	5.7%	−0.05 [−0.35, 0.25]
Jorstad 2013	−0.01	2.2	725	0.02	2.05	715	6.1%	−0.03 [−0.25, 0.19]
Santo 2019	−0.5	2.06	208	−0.5	2.01	107	4.8%	0.00 [−0.47, 0.47]
Goodman 2008	−0.18	2.46	170	−0.2	1.88	162	4.8%	0.02 [−0.45, 0.49]
Maddison 2019	0.23	1.81	150	0.1	1.7	152	5.2%	0.13 [−0.27, 0.53]
Brügemann 2007	0.02	1.89	119	−0.15	2.09	122	4.6%	0.17 [−0.33, 0.67]
Wang 2016	−0.35	1.9	128	−0.62	1.9	128	4.8%	0.27 [−0.20, 0.74]
Varnfield 2014	−0.26	2.01	62	−0.63	1.84	30	3.1%	0.37 [−0.46, 1.20]
**Total (95% CI)**			**5602**			**5874**	**100.0%**	**−0.23 [−0.43, −0.04]**

Heterogeneity: $Tau^2 = 0.16$; $Chi^2 = 106.05$, df = 19 (P < 0.00001); $I^2 = 82\%$
Test for overall effect: Z = 2.32 (P = 0.02)

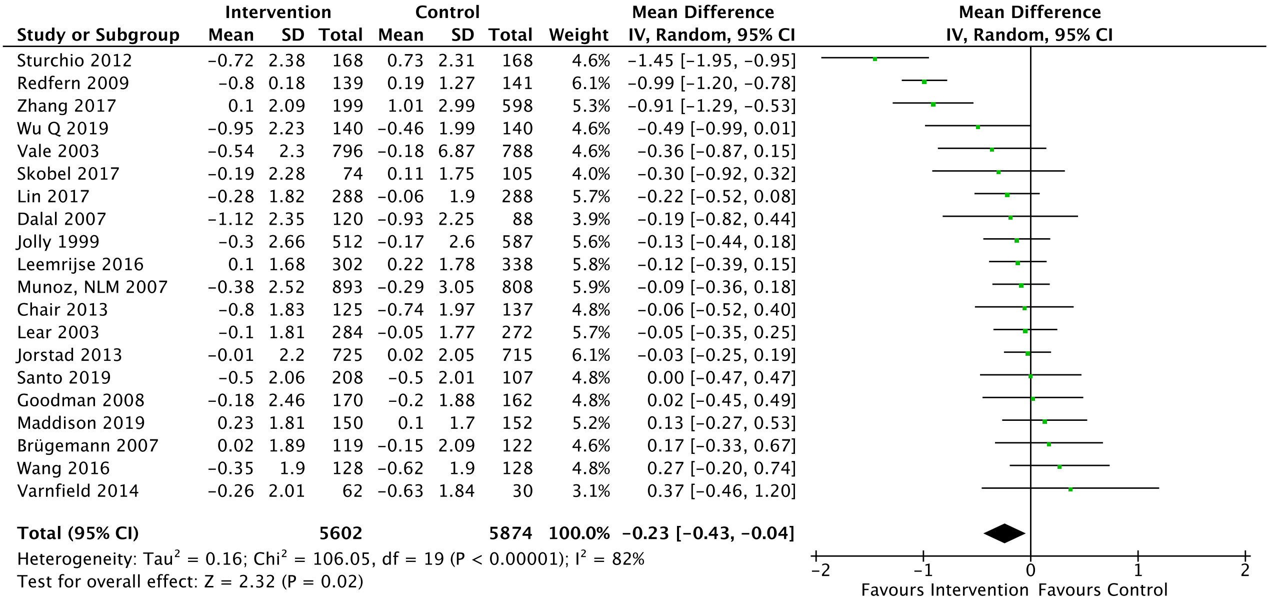

(E) LDL cholesterol

	Intervention			Control				Mean Difference
Study or Subgroup	Mean	SD	Total	Mean	SD	Total	Weight	IV, Random, 95% CI
Brügemann 2007	−0.19	1.84	119	−0.25	1.8	122	3.5%	0.06 [−0.40, 0.52]
Chair 2013	−0.73	1.6	125	−0.62	1.61	137	4.2%	−0.11 [−0.50, 0.28]
Du 2016	−0.26	1.74	958	−0.07	1.91	970	7.2%	−0.19 [−0.35, −0.03]
Ho 2014	−0.34	1.79	244	−0.3	1.46	238	5.4%	−0.04 [−0.33, 0.25]
Johnston 2016	−1.8	2.01	120	−1	1.61	110	3.4%	−0.80 [−1.27, −0.33]
Jorstad 2013	−0.1	1.93	732	0.03	1.66	734	6.9%	−0.13 [−0.31, 0.05]
Lear 2003	−0.09	1.55	284	−0.07	1.43	272	6.0%	−0.02 [−0.27, 0.23]
Leemrijse 2016	0.07	1.77	302	0.1	1.49	332	5.9%	−0.03 [−0.29, 0.23]
Lin 2017	−0.39	1.56	288	−0.02	1.73	288	5.7%	−0.37 [−0.64, −0.10]
Maddison 2019	0.15	1.69	150	0.03	1.2	152	4.9%	0.12 [−0.21, 0.45]
Munoz, NLM 2007	−0.31	1.83	893	−0.26	2.32	808	6.7%	−0.05 [−0.25, 0.15]
Redfern 2009	−0.6	0.18	139	−0.2	0.18	141	8.3%	−0.40 [−0.44, −0.36]
Santo 2019	−0.58	1.77	208	−0.52	1.64	107	4.2%	−0.06 [−0.45, 0.33]
Sturchio 2012	−0.78	2.31	168	0.13	2.18	168	3.3%	−0.91 [−1.39, −0.43]
Vaillant 2015	−0.39	0.72	68	−0.49	0.59	78	6.4%	0.10 [−0.12, 0.32]
Vale 2003	−0.55	1.56	774	−0.21	1.98	769	7.0%	−0.34 [−0.52, −0.16]
Varnfield 2014	−0.13	1.41	62	−0.39	1.48	26	2.1%	0.26 [−0.41, 0.93]
Wu Q 2019	−0.73	1.75	140	−0.58	1.69	140	4.1%	−0.15 [−0.55, 0.25]
Zhang 2017	−0.8	1.59	200	−0.4	1.72	198	4.9%	−0.40 [−0.73, −0.07]
**Total (95% CI)**			**5974**			**5790**	**100.0%**	**−0.18 [−0.29, −0.07]**

Heterogeneity: $Tau^2 = 0.04$; $Chi^2 = 82.07$, df = 18 ($P < 0.00001$); $I^2 = 78\%$
Test for overall effect: Z = 3.13 (P = 0.002)

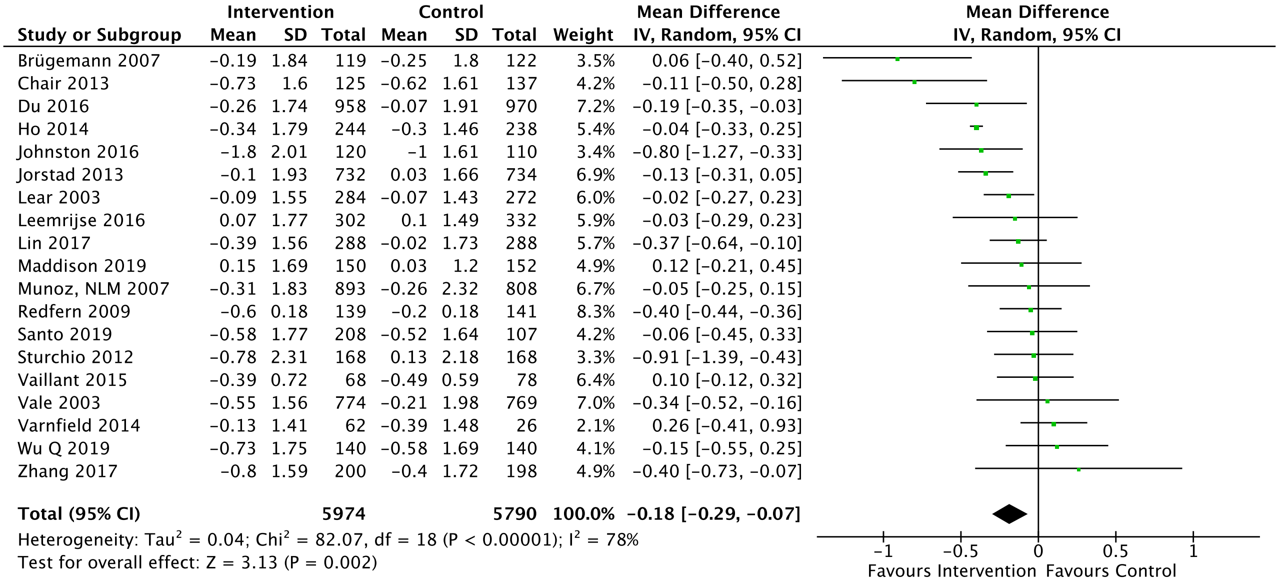

(F) Triglycerides

	Intervention			Control				Mean Difference
Study or Subgroup	Mean	SD	Total	Mean	SD	Total	Weight	IV, Random, 95% CI
Wu Q 2019	−0.78	2.31	168	0.13	2.18	168	4.3%	−0.91 [−1.39, −0.43]
Lin 2017	−1.8	2.01	120	−1	1.61	110	4.4%	−0.80 [−1.27, −0.33]
Varnfield 2014	−0.6	0.18	139	−0.2	0.18	141	9.6%	−0.40 [−0.44, −0.36]
Sturchio 2012	−0.39	1.56	288	−0.02	1.73	288	7.0%	−0.37 [−0.64, −0.10]
Jorstad 2013	−0.26	1.74	958	−0.07	1.91	970	8.5%	−0.19 [−0.35, −0.03]
Maddison 2019	−0.1	1.93	732	0.03	1.66	734	8.2%	−0.13 [−0.31, 0.05]
Chair 2013	−0.73	1.6	125	−0.62	1.61	137	5.3%	−0.11 [−0.50, 0.28]
Wang 2016	−0.58	1.77	208	−0.52	1.64	107	5.3%	−0.06 [−0.45, 0.33]
Lear 2003	−0.34	1.79	244	−0.3	1.46	238	6.6%	−0.04 [−0.33, 0.25]
Redfern 2009	0.07	1.77	302	0.1	1.49	332	7.2%	−0.03 [−0.29, 0.23]
Vale 2003	−0.17	1.83	893	−0.14	2.32	808	8.0%	−0.03 [−0.23, 0.17]
Munoz, NLM 2007	−0.09	1.55	284	−0.07	1.43	272	7.3%	−0.02 [−0.27, 0.23]
Brügemann 2007	−0.19	1.84	119	−0.25	1.8	122	4.5%	0.06 [−0.40, 0.52]
Zhang 2017	−0.39	0.72	68	−0.49	0.59	78	7.7%	0.10 [−0.12, 0.32]
Vaillant 2015	0.15	1.69	150	0.03	1.2	152	6.1%	0.12 [−0.21, 0.45]
**Total (95% CI)**			**4798**			**4657**	**100.0%**	**−0.17 [−0.30, −0.03]**

Heterogeneity: $Tau^2 = 0.05$; $Chi^2 = 79.28$, df = 14 ($P < 0.00001$); $I^2 = 82\%$
Test for overall effect: Z = 2.42 (P = 0.02)

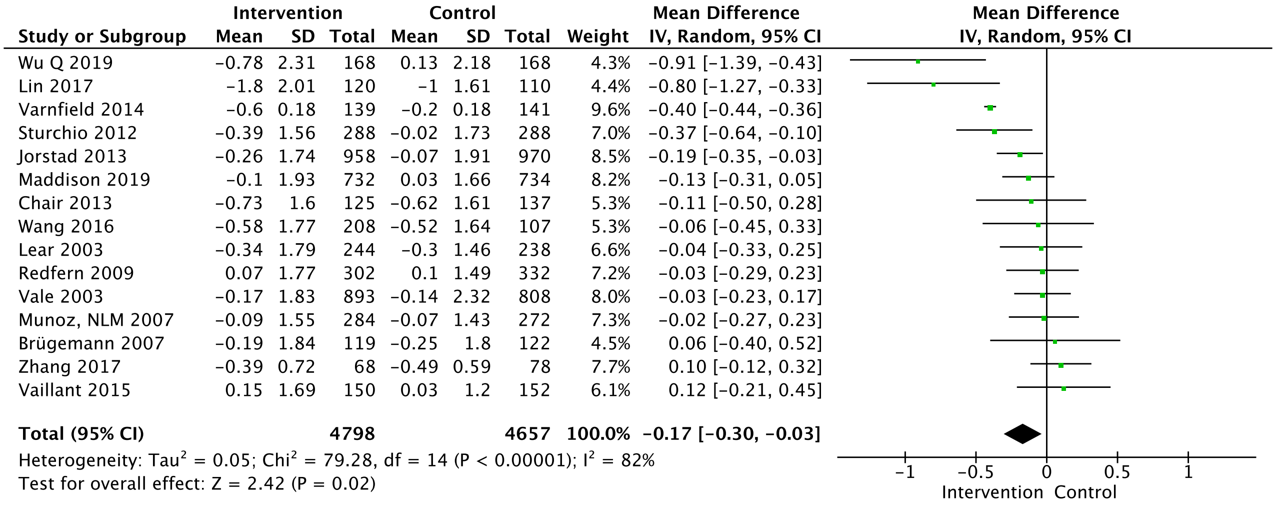

(G) BMI

Study or Subgroup	Intervention Mean	Intervention SD	Intervention Total	Control Mean	Control SD	Control Total	Weight	Mean Difference IV, Fixed, 95% CI
Wang 2016	−2.51	7.68	128	−0.03	6.98	128	1.5%	−2.48 [−4.28, −0.68]
Alsaleh 2016	−1.86	10.84	132	0.45	11.61	158	0.7%	−2.31 [−4.90, 0.28]
Minneboo 2017	−1.5	11.36	720	0.35	9.06	702	4.3%	−1.85 [−2.92, −0.78]
Skobel 2017	−1.57	10.67	74	0.11	7.53	105	0.6%	−1.68 [−4.51, 1.15]
Dalal 2007	0.43	6.37	120	1.86	6.99	88	1.4%	−1.43 [−3.28, 0.42]
Redfern 2009	−0.6	1.38	139	0.7	1.39	141	46.7%	−1.30 [−1.62, −0.98]
Zhang 2017	−1.2	6.35	200	−0.4	7.39	198	2.7%	−0.80 [−2.15, 0.55]
Chair 2013	−0.8	7.81	125	−0.2	7.11	137	1.5%	−0.60 [−2.41, 1.21]
Vale 2003	−0.45	6.91	796	−0.14	6.95	788	10.6%	−0.31 [−0.99, 0.37]
Jorstad 2013	−0.2	8.65	725	0.03	7.37	715	7.1%	−0.23 [−1.06, 0.60]
Lear 2003	−0.1	8.91	293	0.1	7	287	2.9%	−0.20 [−1.50, 1.10]
Chaves, GSS 2019	0	11.95	44	0.2	8.17	55	0.3%	−0.20 [−4.34, 3.94]
Johnston 2016	−0.6	11.17	166	−0.5	9.44	152	1.0%	−0.10 [−2.37, 2.17]
Vaillant 2015	−0.35	7.2	84	−0.27	9.33	102	0.9%	−0.08 [−2.46, 2.30]
Leemrijse 2016	0	7.07	316	0	40.03	344	0.3%	0.00 [−4.30, 4.30]
Maddison 2019	0.04	9.16	147	−0.04	6.98	149	1.4%	0.08 [−1.78, 1.94]
Goodman 2008	−0.08	8.44	184	−0.16	8.07	186	1.7%	0.08 [−1.60, 1.76]
Wu Q 2019	−0.04	5.55	140	−0.22	7.85	140	1.9%	0.18 [−1.41, 1.77]
Jolly 1999	0.4	6.48	554	0.2	5.81	640	10.0%	0.20 [−0.50, 0.90]
Yu 2020	0.46	17.54	995	0.23	14.79	993	2.4%	0.23 [−1.20, 1.66]
**Total (95% CI)**			**6082**			**6208**	**100.0%**	**−0.83 [−1.05, −0.61]**

Heterogeneity: $Chi^2 = 37.00$, df = 19 (P = 0.008); $I^2 = 49\%$
Test for overall effect: Z = 7.33 (P < 0.00001)

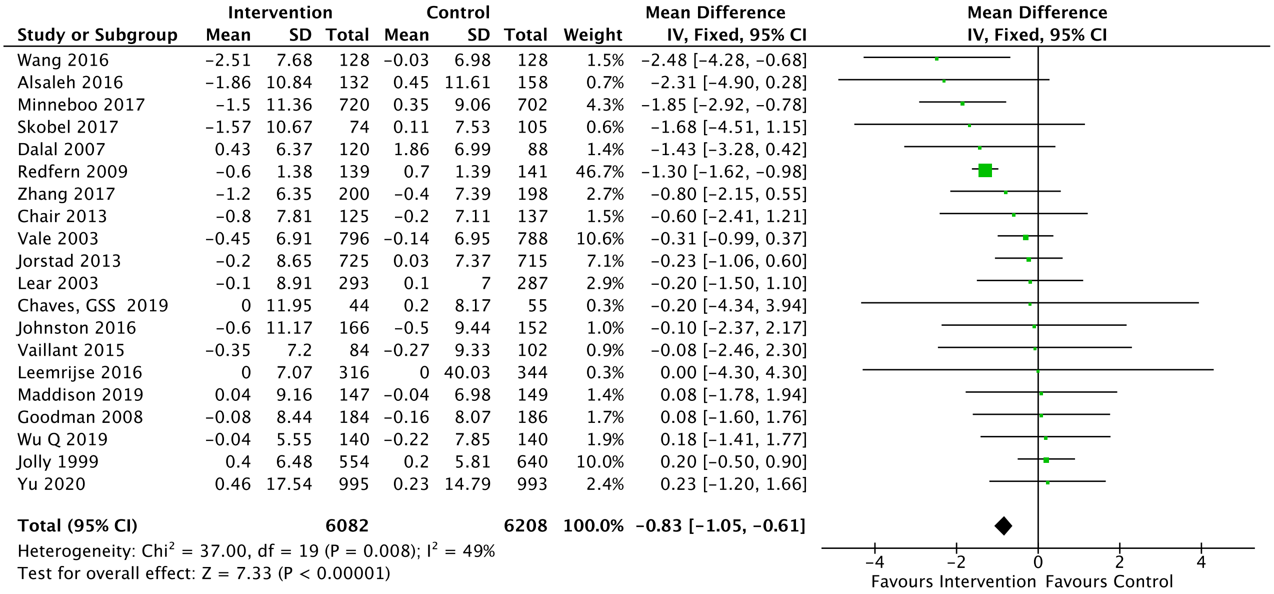

Footnotes: Forest plot was derived from a fixed-effects or random-effect meta-analysis model, respectively. To convert fasting plasma glucose to mmol/L, divide by 18; total cholesterol and LDL-C to mmol/L, multiply by 0.02586; triglycerides to mmol/L, multiply by 0.01129. SD, standard deviation; 95% CI, 95% confidence interval.

Figure S10. Association of multicomponent integrated care on cardiovascular-related mortality among patients with acute coronary syndrome, stratified by the proportion of antiplatelet/ antithrombotic therapy

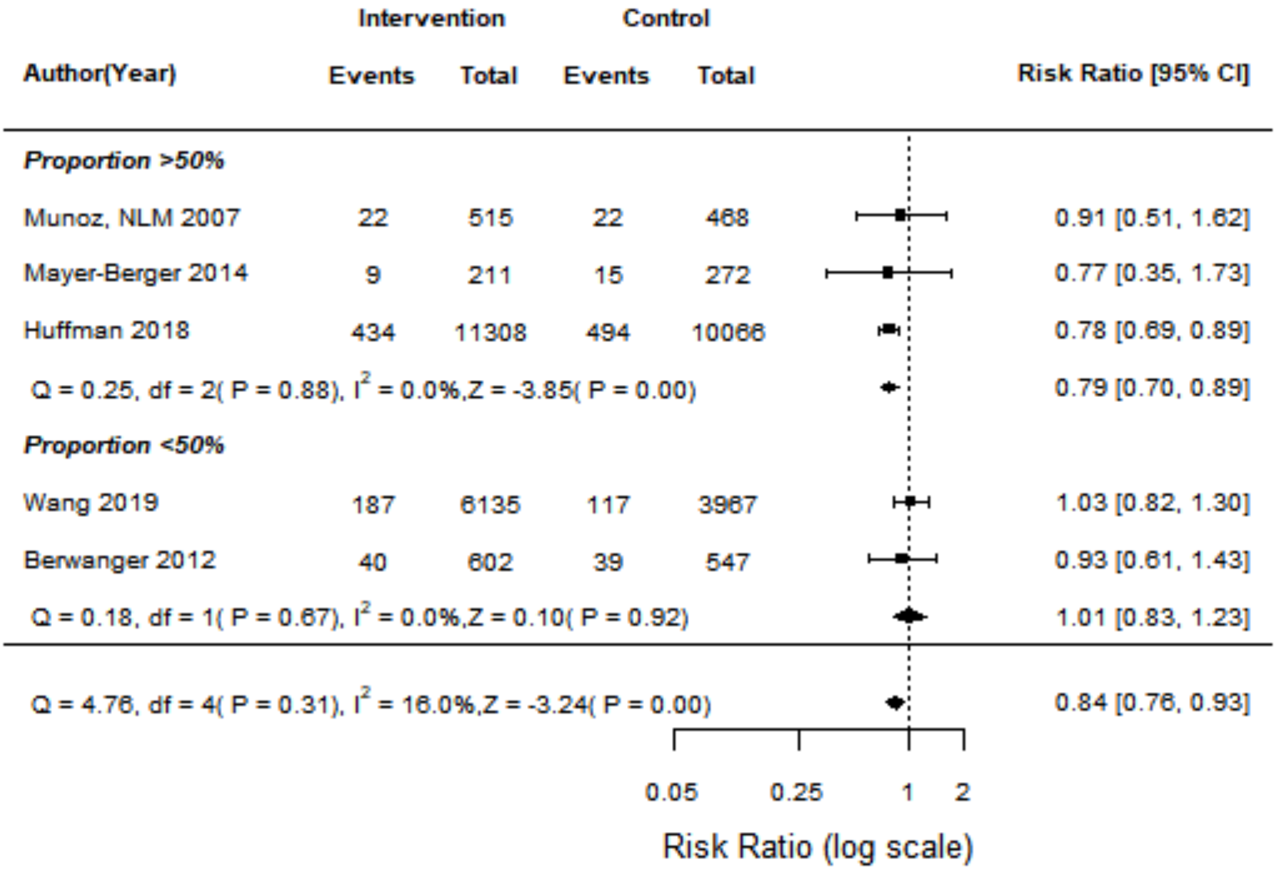

Footnotes: 95% CI, 95% confidence interval.

Figure S11. Association of multicomponent integrated care on all-cause hospitalization among patients with acute coronary syndrome, stratified by (A) age groups, (B) the proportion of antiplatelet/ antithrombotic therapy and (C) the proportion of lipid-lowering drugs

(A)

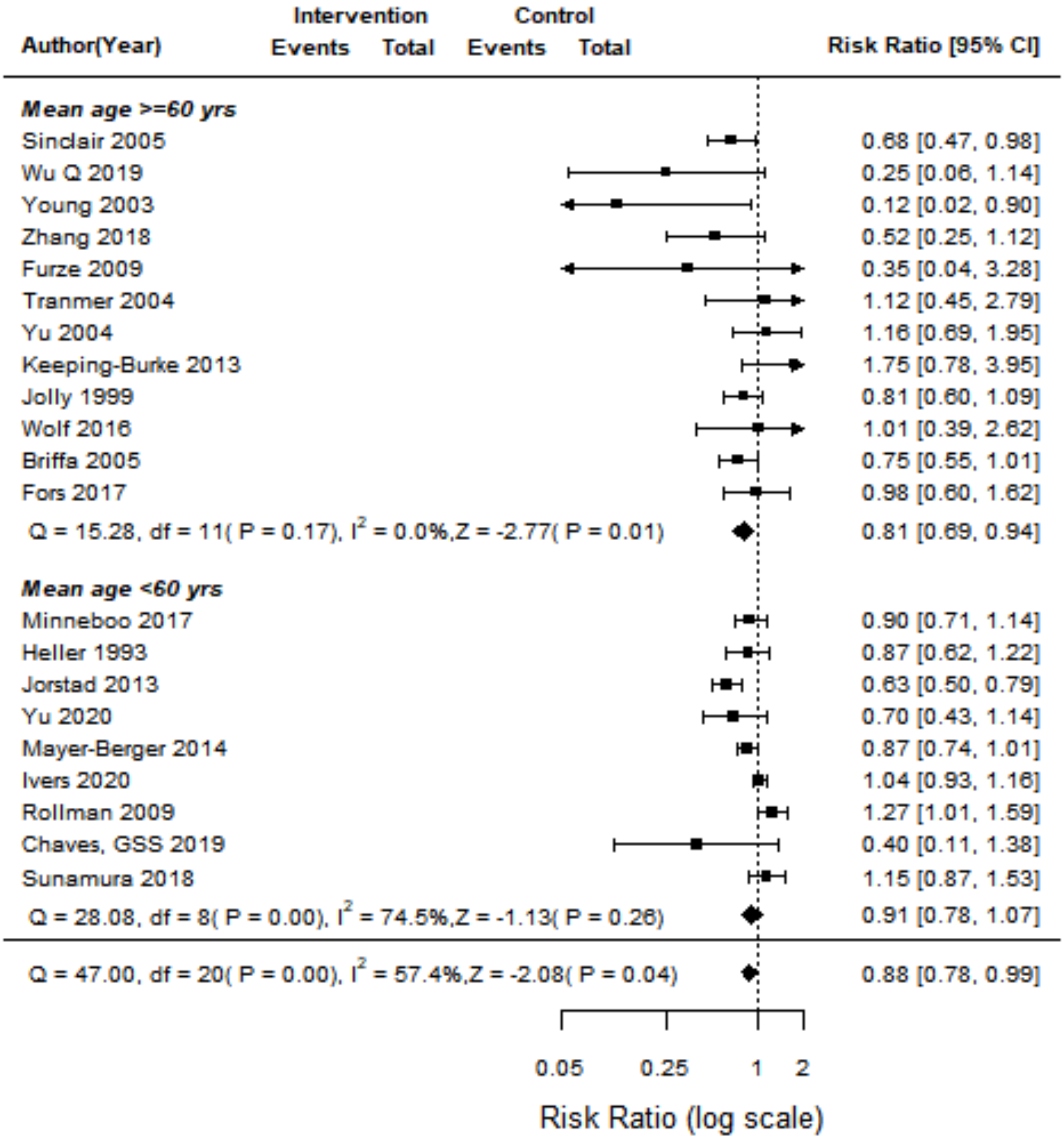

(B)

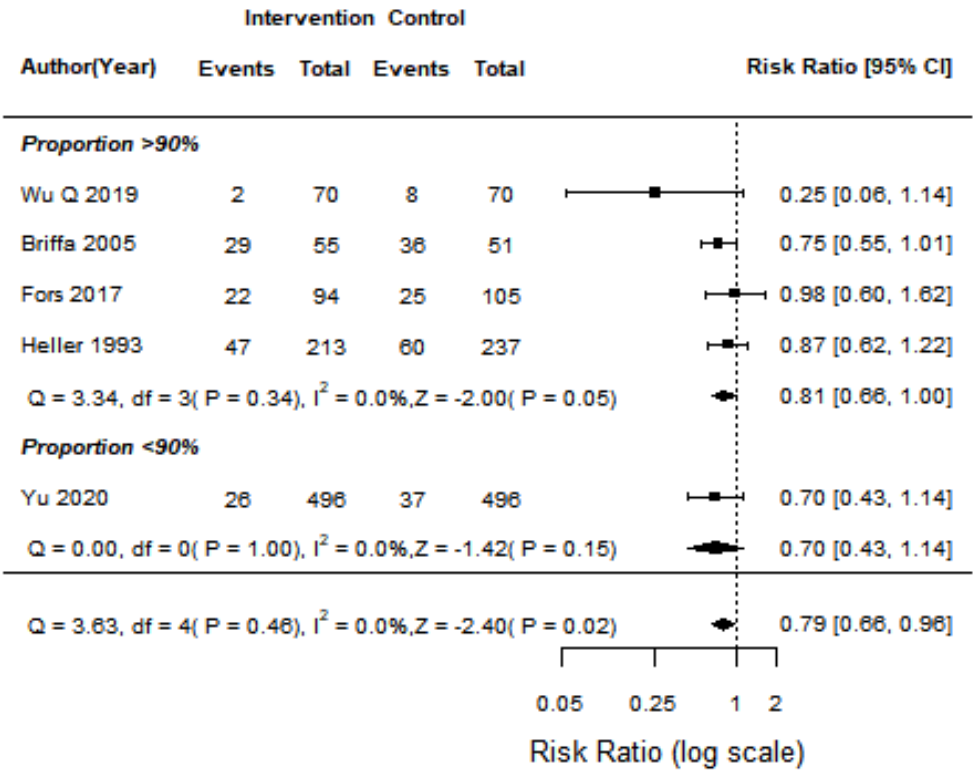

(C)

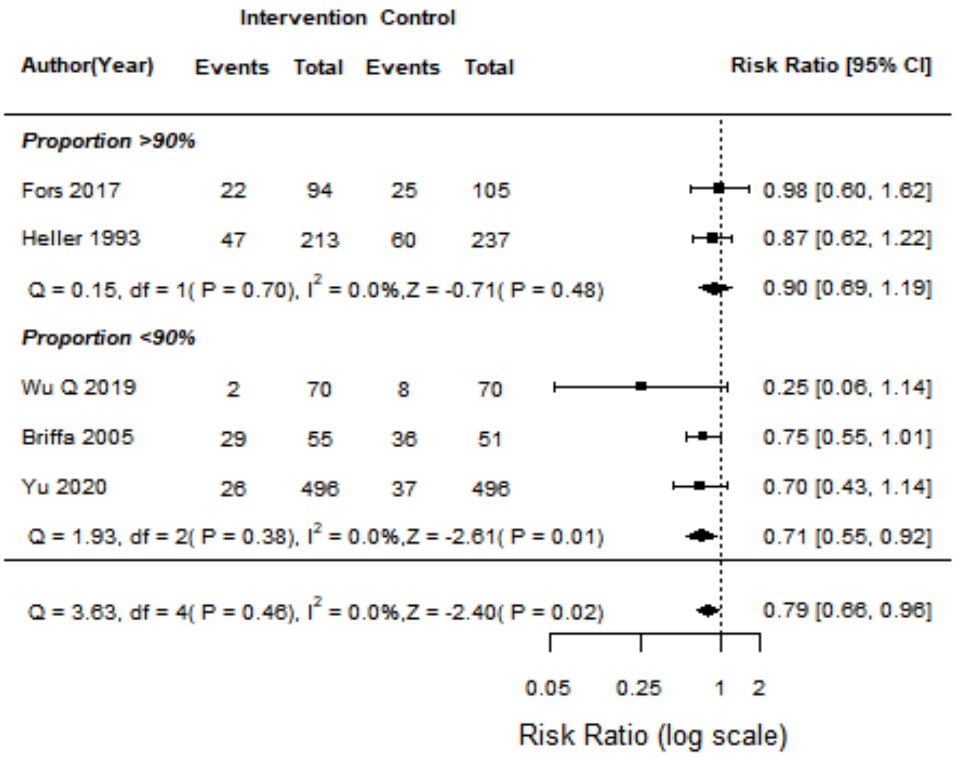

Footnotes: 95% CI, 95% confidence interval.

Figure S12. Meta-analysis results of multicomponent integrated care on cardiovascular hospitalization among patients with acute coronary syndrome, stratified by age groups

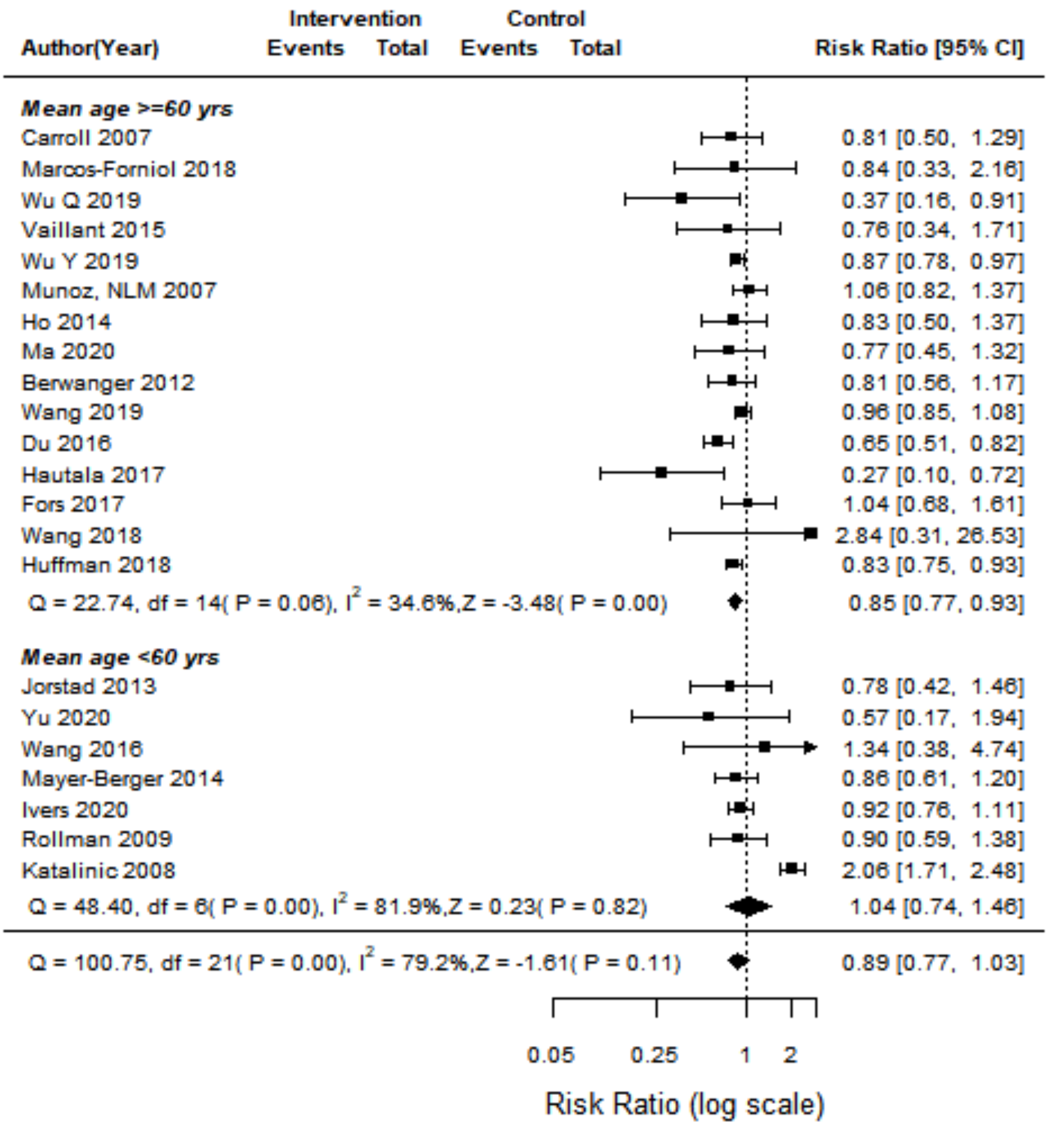

Footnotes: 95% CI, 95% confidence interval.

Figure S13. Funnel plots

(A) All-cause mortality

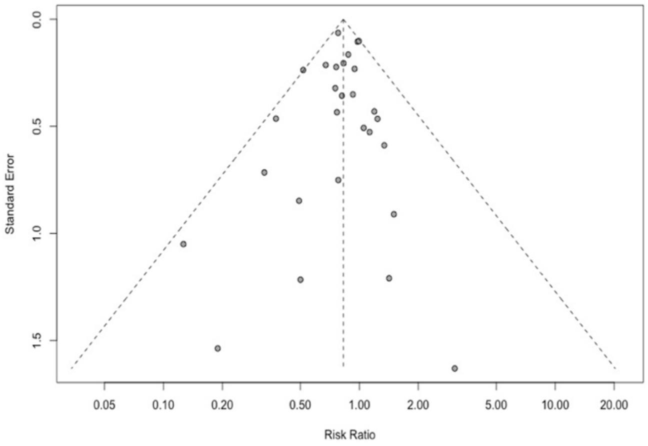

(B) Cardiovascular mortality

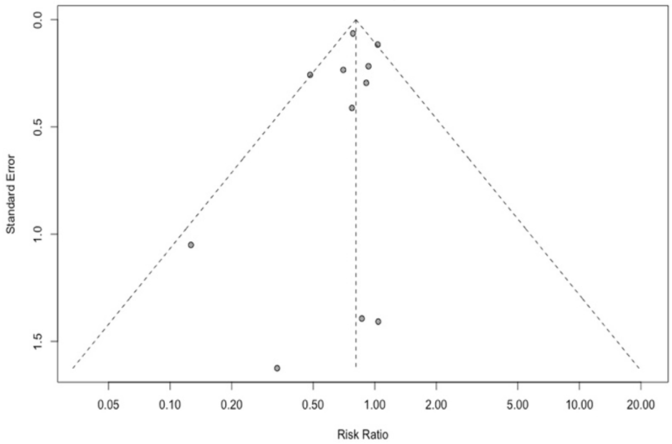

(C) All-cause hospitalization

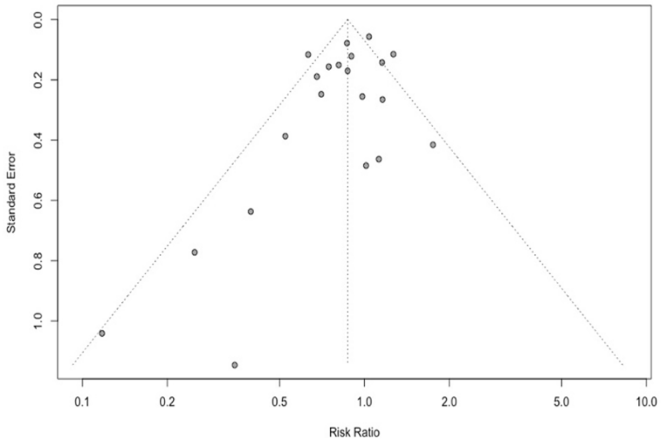

(D) Cardiovascular-related hospitalization

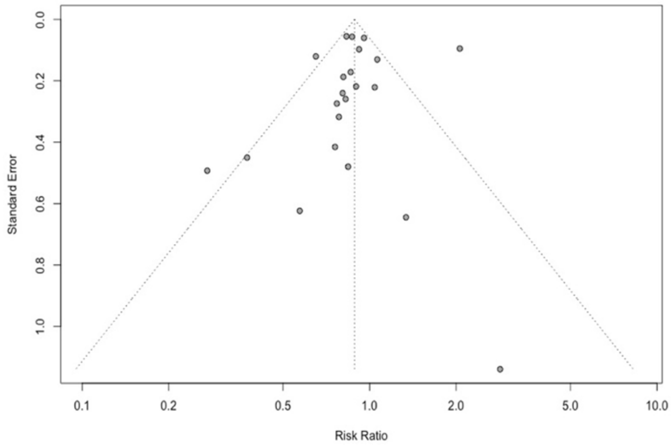

(E) Emergency department visit

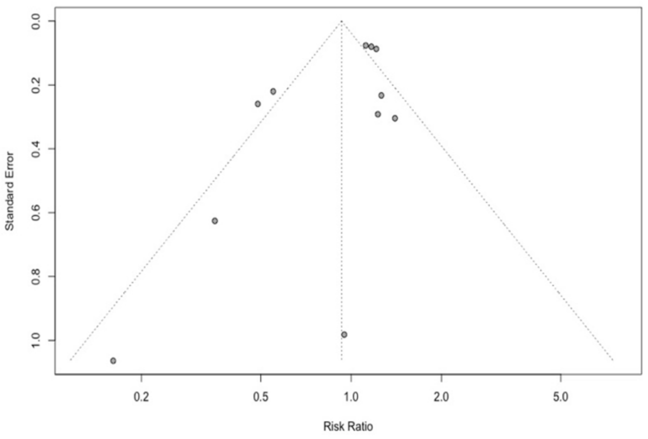

(F) Unplanned outpatient visit

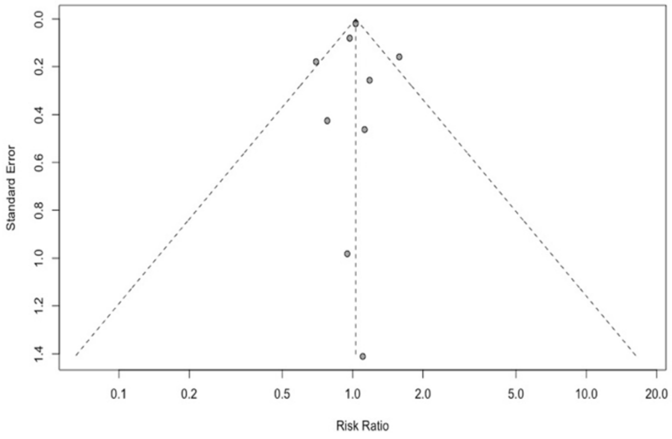

(G) Fasting Plasma Glucose

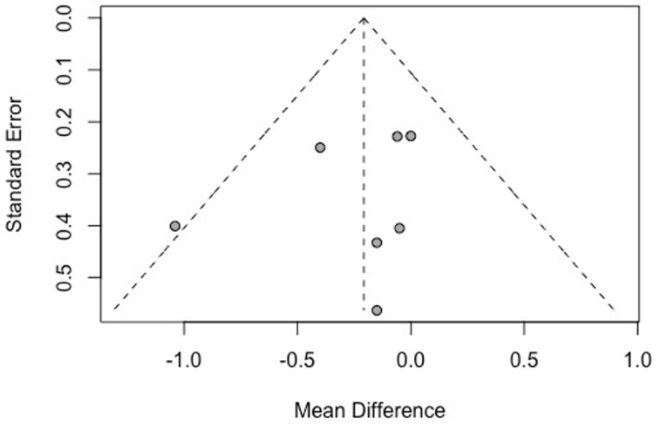

(H) Systolic Blood Pressure

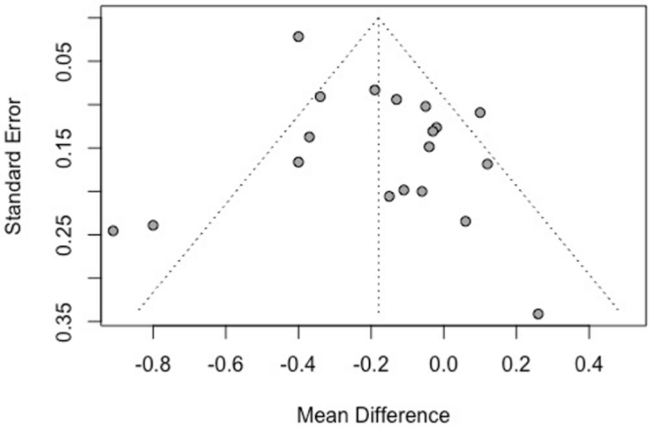

(I) Diastolic Blood Pressure

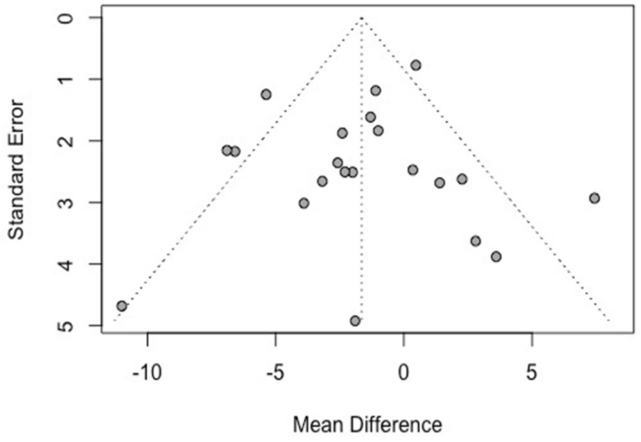

(J) Total Cholesterol

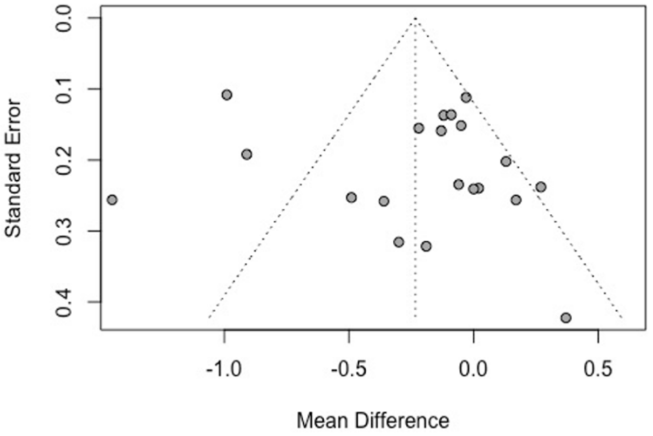

(K) LDL cholesterol

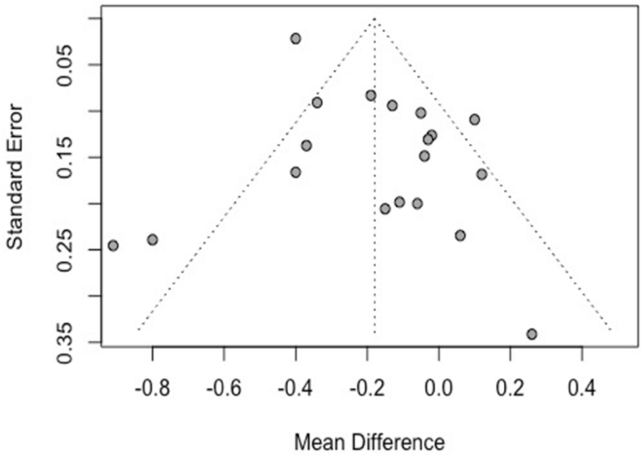

(L) Triglyceride

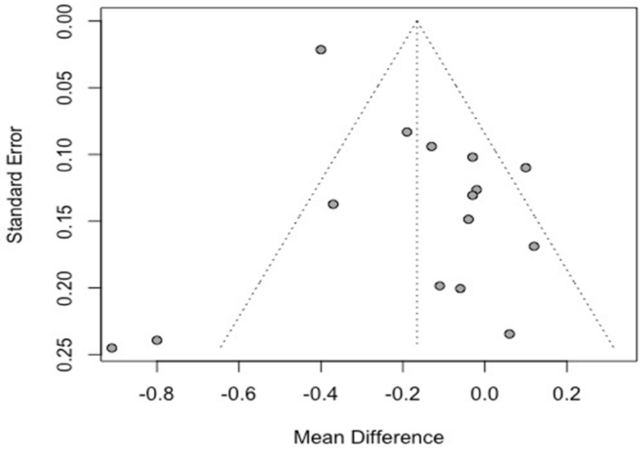

(M) Body Mass Index

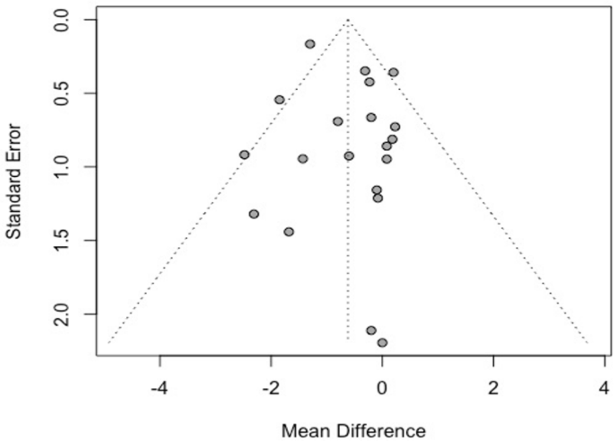

Table S1. Search strategy for implementation of multicomponent integrated care among patients after acute coronary syndrome

1.	"Structured" OR "tailored" OR "shared" OR "multidisciplinary" OR "interdisciplinary" OR "multicomponent" OR "multifaceted" OR "integrated" OR "coordinate*" OR "collaborat*" OR "Quality"
2.	“care” OR “treat*” OR “service*” OR “team*” OR “plan*” OR “therapy” OR “intervention”
3.	#1 AND #2
4.	“Education” OR “self-management” OR “Peer*” OR “nurse*” OR “dietitian*” OR “pharmacist*” OR “Tele*” OR “electronic health” OR “e-health” OR “digital*”
5.	#3 AND #4
6.	“Myocardial Infarction” [Mesh] OR “post-myocardial infarction” OR “post-myocardial*” OR “MI” OR “STEMI” OR “NSTEMI” OR “coronary*” OR “myocardial*” OR “coronary heart disease” OR “ischemic heart disease” OR “coronary artery disease” OR “myocardial ischemia”
7.	“Quality improvement” OR “Quality assurance” OR "Quality of Life"[Mesh] OR “hospital admission” OR “readmission*” OR "Hospitalization"[Mesh] OR “mortality” OR “fatality”
8.	#5 AND #6 AND #7
9.	Filters applied:<br>i. Publication date (custom date range): 1980/01/01 – 2020/11/1<br>ii. English language<br>iii. Human species<br>iv. Clinical study, clinical trial, randomized controlled trial, pragmatic clinical trial, controlled clinical trial

Table S2. Definitions of quality improvement strategies in acute coronary syndrome (Note: Definitions were with reference to a meta-analysis in type 2 diabetes (1))

**(A) Healthcare system**	
**Subcategory**	**Definition(s)**
Case management	Collaboration of standard care of patients in coordination with, or additional to the cardiologist by:<br>i. Personnel: healthcare providers, trained peers or community health workers, social workers.<br>ii. The existence of a multidisciplinary team.
Team change	Modification to the structure or organization of the healthcare team, and there are any of the following situations:<br>i. Add a team member or integrated care/joint visits, e.g. routine visits from cardiac specialist nurses, pharmacists<br>ii. use of a multidisciplinary team, e.g. medicine, nursing, pharmacy, nutrition, psychology.<br>iii. expansion or revision of professional roles, e.g. prescription autonomy to nurses or pharmacists.<br>If at least two of the above conditions are met, a study with "case management" may be eligible for "team change".
Electronic patient registry	Create and outline a new electronic medical record or tracking system, or upgrade the previous electronic system during the study period.
Facilitated relay of patient's information to clinicians	Exchange of health information between patients and health care providers using methods other than traditional medical records e.g. personalized reports, trained peers or community health workers, structured self-monitoring of blood pressure/dietary/exercise diaries, electronic transmission of self-care data.<br>Included internist and patients, feedback meetings with trained peers or community health workers on subsequent changes to the patient's treatment plan and improved referral system in an out-of-office consultation setting.<br>This information must get to someone with prescribing and ordering ability.
Using electronic health (e-health) with team support	Involves the adoption of software or electronic applications to promote better post-discharge care after acute coronary syndrome, e.g. telemedicine, mobile health (m-Health), e-Learning platform (smartphone applications, short messaging service, automated educational messaging, multimedia use, emails, personal digital assistant).<br>Included intensified application of electronic databases, i.e. integration, analysis, interpretation and communication of the information to healthcare team and patients (e.g. electronic patient's report card, risk assessment analysis).
Continuous quality improvement	A repetitive process of examining the effects, checking of quality issues, yielding potential solutions, and re-evaluate for further action if necessary (plan-do-study-act cycles, quality assurance).<br>Assessing on fidelity of intervention or intervention delivery feedback by trained peers or community health workers with solutions provided to improve patient's care.

(B) Healthcare providers	
**Subcategory**	**Definition(s)**
Audit and feedback	Standard reports on the healthcare providers or practices on care processes clinical performance.
Clinician education	Continuous provision of up-to-date post-discharged after acute coronary syndrome care management and guidelines to all healthcare providers, e.g. conferences/workshops, distribution of educational materials (written, video etc.), and academic detailing.<br>If the education was related to the workflow of usual care model implementation, it was not categorized as clinician education.
Clinician reminders	Paper-based or electronic system prompts to healthcare providers on patient-specific information (biomedical data or care processes), including ad-hoc clinician reminders.<br>It is sub-classified as decision support with the provision of treatment algorithms and/or protocols to healthcare providers.
Financial incentive (pay for performance)	Could be positive or negative financial incentives related to healthcare performance that were provided to healthcare providers and patients.<br>Patients: changes in reimbursement as a token of achievement after participation in the program (e.g. capitation, prospective payment, or a shift from fee-for-service to salary pay structure), lower annual fee in case of treatment targets attainment.<br>Excluded transport reimbursement, honorarium, gift cards, or stipend to patients, healthcare providers or trained peer/community health workers for any study procedures unless they contribute to clinical outcomes.
**(C) Patients**	
**Subcategory**	**Definition(s)**
Patient's education	To promote better understanding of acute coronary syndrome and related topics, as well as adoption of positive attitudes towards their active participation in care improvement of their disease, delivered by individual or group sessions with allied health personnel or trained peer/community health workers.<br>Distribution of printed/electronic educational materials or patient's report card.
Promotion of self-management	Distribution of patient's report card or equipment (e.g. sphygmomanometer acute coronary syndrome survivors remain at higher risk, particularly older individuals and patients with comorbid hypertension, diabetes, peripheral artery disease, or history of stroke).<br>Introduction to resources only after joining education programmes (e.g. online platform for transmission of self-care records to healthcare providers, facilitated adjustments of medication dose, on-site grocery shopping, personalized goal-setting and plan).<br>Collaboration of trained peers or community health workers.
Patient reminder system	Any approaches (e.g. in person, postal mail, live/automated phone calls, mobile texts, web/emails) to remind patients about appointments or important self-care aspects.<br>If case management was included, patient's reminders needed to be explicit and an extra task to the normal case management.

Table S3: Baseline characteristics of trials included in the meta-analysis

**Number of patients, n**	93,278
**Age (years)**	62.4±11.9
**Men**	67,253 (72.1%)
**Types of acute coronary syndrome**	
ST-elevation myocardial infarction (STEMI)	31,335 (33.6%)
Non-ST-elevation myocardial infarction (NSTEMI)	6,185 (6.6%)
Unstable angina (UA)	16,968 (18.2%)
Undefined	38,183 (40.9%)
**Duration of intervention (months), median (interquartile range)**	6 (3-10)
**Duration of follow up (months), median (interquartile range)**	12 (6-12)
**Fasting Plasma Glucose (mmol/L), median (interquartile range)**	6.1 (5.7-6.2)
**Systolic blood pressure (mmHg), median (interquartile range)**	129.7 (125.7-134.5)
**Diastolic blood pressure (mmHg), median (interquartile range)**	76.9 (75.2-79.0)
**Total cholesterol, median (interquartile range)**	4.6 (4.1-4.9)
**LDL-cholesterol (mmol/L), median (interquartile range)**	2.7 (2.2-2.9)
**Triglyceride (mmol/L), median (interquartile range)**	1.7 (1.5-1.2)
**Body mass index (BMI), median (interquartile range)**	28.0 (26.1-28.9)
**Number of studies per quality improvement strategy**	
**Health system**	
Case management	6 (8.1%)
Team change	62 (83.8%)
Electronic patient registry	1 (1.4%)
Facilitated relay of patient's information to clinicians	40 (54.1%)
Electronic health	14 (18.9%)
Continuous quality improvement	24 (32.4%)
Healthcare providers	
Audit and feedback	9 (12.2%)
Clinician education	20 (27.0%)
Clinician reminder/decision support	17 (23.0%)
Financial incentives	1 (1.4%)
Patient	
Patient education	46 (62.2%)
Promotion of self-management	31 (41.9%)
Patient reminder system	39 (52.7%)

Footnotes: All the data are indicated as number (percentages) or median (interquartile range [IQR]), except age in mean±standard deviation.

*To convert fasting plasma glucose to mmol/L, divide by 18; total cholesterol and LDL-C to mmol/L, multiply by 0.02586; triglycerides to mmol/L, multiply by 0.01129.

Table S4: Effects of individual quality improvement strategies on mortality and hospitalization (all-cause and cardiovascular-related) among patients with acute coronary syndrome

	**All-cause mortality**		**Cardiovascular-related mortality**		**All-cause hospitalization**		**Cardiovascular-related hospitalization**	
**QI strategy**	**N**	**RR (95% CI)**	**N**	**RR (95% CI)**	**N**	**RR (95% CI)**	**N**	**RR (95% CI)**
**Healthcare system**								
Case Management	1	0.77 (0.33-1.80)	1	0.86 (0.06-13.28)	2	0.29 (0.10-0.83)	1	0.27 (0.10-0.73)
Team Change	24	0.87 (0.78-0.97)	24	0.92 (0.77-1.10)	17	0.84 (0.73-0.97)	19	0.86 (0.71-1.05)
Electronic Patient Registry	0	N/A	0	N/A	0	N/A	0	N/A
Facilitated Patient Relay	13	0.79 (0.66-0.95)	13	0.55 (0.36-0.84)	13	0.90 (0.77-1.05)	13	0.85 (0.66-1.09)
Electronic Health	3	0.86 (0.59-1.26)	3	0.93 (0.60-1.43)	4	0.91 (0.70-1.19)	5	0.87 (0.78-0.97)
Continuous QI	5	0.82 (0.75-0.90)	5	0.76 (0.68-0.86)	6	0.85 (0.58-1.24)	6	0.82 (0.72-0.94)
**Healthcare providers**								
Audit and Feedback	3	0.77 (0.68-0.87)	3	0.78 (0.69-0.88)	2	0.70 (0.55-0.89)	3	0.84 (0.78-0.91)
Clinician Education	12	0.84 (0.77-0.91)	12	0.83 (0.67-1.02)	6	0.80 (0.63-1.01)	10	0.87 (0.81-0.94)
Clinician Reminder	7	0.90 (0.77-1.06)	7	0.81 (0.62-1.06)	1	0.12 (0.02-0.90)	6	1.03 (0.70-1.52)
Financial Incentive	1	1.00 (0.83-1.21)	1	1.03 (0.82-1.30)	0	N/A	1	0.96 (0.85-1.08)
**Patients**								
Patient Education	15	0.80 (0.73-0.88)	15	0.77 (0.68-0.87)	12	0.84 (0.71-0.99)	15	0.90 (0.73-1.11)
Self-management	11	0.77 (0.64-0.93)	11	0.70 (0.44-1.11)	9	1.03 (0.91-1.16)	7	1.15 (0.77-1.72)
Patient Reminder	18	0.90 (0.79-1.02)	18	0.84 (0.61-1.15)	13	0.97 (0.86-1.10)	12	0.92 (0.85-1.00)

Footnotes: Continuous QI, Continuous quality improvement; CV-related hospitalization, Cardiovascular-related hospitalization; N, number of trials involved; RR (95% CI), Risk ratio (95% confidence interval); N/A, not available; QI strategy, Quality improvement strategy.

Table S5: Medication Use

Care processes	Definitions used in available trials	Total number of available trials	Number of trials which reported significant improvement with multi-component integrated care (Reference no.)	Number of trials which reported no between group difference (Reference no.)
Use of antiplatelet or antithrombotic therapy	Aspirin only	15	Berwanger, O 2012 (2)	Mayer-Berger W 2014 (3); Williams JB 2011 (4); MEDMAN 2007 (5); Lear, S. A 2003 (6)
	Aspirin or clopidogrel		Ivers, N. M. 2020 (7); Munoz MA 2007(8); Wang 2019(9)	Minneboo 2017 (10); Dalal, H 2007 (11); Huffman, M. D. 2018 (12); Mosca, L 2010 (13); Yu C 2020 (14); Redfern J 2008 (15)
	Aspirin or warfarin		N/A	Sturchio, A 2012 (16)
Use of renin-angiotensin system inhibitors	ACE inhibitors only	12	Ivers, N. M. 2020 (7); Mosca, L 2010 (13); Williams JB 2011 (4); Munoz MA 2007 (8)	Dalal, H 2007 (11); Mayer-Berger W 2014 (3); Sturchio, A 2012 (16)
	ARBs only		N/A	N/A
	ACE inhibitors or ARBs		Jørstad, H. T. 2016 (17); Yu C 2020(14); Lear, S. A 2003(6)	Minneboo 2017(10); Redfern J 2008(15)
Use of lipid lowering agents	Statin only	14	Redfern J 2008 (15); Sturchio, A 2012(16)	Ivers, N. M. 2020 (7); Yu C 2020 (14); Lear, S. A 2003 (6)
	Statin, fibrate or ezetimibe		Minneboo 2017 (10); Dalal, H 2007 (11); Lapointe, F 2006 (18); Williams JB 2011(4); Munoz MA 2007 (8)	Jørstad, H. T. 2016 (17); Mayer-Berger W 2014 (3); Carlsson R 1998 (19); MEDMAN 2007 (5)

Footnotes: ACE inhibitors, Angiotensin-converting enzyme inhibitors; ARBs, Angiotensin receptor blockers; N/A, not available.

Table S6: Meta-regression analysis of associations between multicomponent integrated care and study outcomes among patients with acute coronary syndrome

	**Mortality**						**Hospitalization**						**Visit**					
	**All cause**			**Cardiovascular-related**			**All cause**			**Cardiovascular-related**			**Emergency department**			**Unplanned outpatient**		
	**β**	**se**	**p**	**β**	**se**	**p**	**β**	**se**	**p**	**β**	**se**	**p**	**β**	**se**	**p**	**β**	**se**	**p**
Age	0.009	0.008	0.271	0.016	0.025	0.514	-0.015	0.005	**0.003**	-0.019	0.005	**<0.001**	-0.007	0.014	0.634	0.001	0.002	0.730
Male proportion	-0.459	0.643	0.475	-2.280	1.327	0.086	1.449	0.742	0.051	0.308	0.613	0.616	1.427	1.442	0.322	-0.738	0.412	0.073
Antiplatelet/ antithrombotic therapy	-0.248	0.135	0.067	-0.321	0.149	**0.031**	-1.197	0.586	**0.041**	-0.137	0.092	0.135	N/A	N/A	N/A	N/A	N/A	N/A
RAAS inhibitors	-0.693	0.484	0.152	N/A	N/A	N/A	0.962	1.207	0.425	-0.400	0.358	0.264	N/A	N/A	N/A	N/A	N/A	N/A
Lipid-lowering drugs	-1.048	0.878	0.233	N/A	N/A	N/A	-1.624	0.651	0.013	-0.733	0.563	0.193	N/A	N/A	N/A	N/A	N/A	N/A

Footnotes: N/A, not available (only 1 or 2 study was included.) RAAS, renin-angiotensin aldosterone system.

Table S7: Study characteristics of trials included in the meta-analysis

Study Setting	Country	Study Population	Age	Gender Male	Socio-economic & Education Status	Baseline SBP	Personnel Involved	Freq & Duration of patient/personnel training	Patients per group	Curriculum	Quality Assurance	Attendance Rates/ Intensity
Maddison, R 2018 (20)												
Metropolitan hospitals, outpatient clinics and community-based cardiac rehabilitation seminars	New Zealand	162 adults with coronary heart disease	61.5 ± 12.2	85.9%	N/A	139.0 ±17.4 vs 134.4 ±17.00	Exercise-based cardiac rehabilitation specialist, clinical exercise physiologists	Patient education (12w) Smartphone and chest-worn wearable sensor enabling audio coaching, feedback and social support throughout prior real-time exercise monitoring.	Individual	Exercise prescription, exercise monitoring and coaching plus theory-based behavioural strategies.	N/A	82.7%
Duan YP 2018(21)												
Outpatients cardiac rehabilitation centre of hospital	China	114 with coronary heart disease	N/A	46.9%	Higher education 89.0%	N/A	Nurse, role models	Patient education (8w) Web-based health program (once a week) with short-message service text as reminder. First 4 weeks on PA and subsequent 4 weeks on fruit and vegetable consumption.	Individual	Tailored electronic health (eHealth) lifestyle promotion: Behaviour change techniques-PA, fruit and vegetable consumption.	N/A	72.8%
Hautala 2016(22)												
Division of Cardiology of University Hospital	Finland	204 CAD patients who suffered from acute coronary syndrome (ACS)	60.0 ± 11.0 vs 62.0 ± 9.0	72.0%	N/A	136.0 ± 22.0 vs 139.0 ±24.0	Physical therapist, Internist	Patient education (12m) Exercise based cardiac rehabilitation: 4-5 exercise sessions on a weekly basis.	Individual followed by groups (<8)	Gym and home-based exercise training with exercise training diary, use of the perceived ratings of exertion (RPE) scale, schedule for gym visits, and use of an accelerometer.	N/A	72.6%
Minneboo 2017(10)												
15 hospitals	Netherlands	824 patients with coronary artery disease (CAD)	58.2 ± 9.0 vs 59.2 ± 9.4	79.0%	>13years 44.0%	100.0 ± 28.0 vs 116.0 ± 33.0	Nurse, Internist, cardiologist & partners	Patient education (12m) Community-based lifestyle programs: Each intervention program takes at least 3 months.	Individual	Community-based lifestyle programs for smoking cessation, weight reduction and PA. If appropriate, partners are encouraged to participate in all programs for free.	N/A	91.2% vs 86.2%
Wang, W 2018(23)												
outpatient clinic in a public hospital	Singapore	129 patients with coronary heart disease (CHD)	60.8 ± 8.32 vs 60.8 ± 9.33	89.2%	Retired: 26.2% vs 39.1% Secondary Education or above 49 (75.4) vs 46 (71.9)	134.4 ± 25.2 vs 137.4 ± 21.8	Research nurse, cardiologists	Patient education (4w) Coronary Heart Disease Self-management Programme (CHDSMP): Home-based self-help psychoeducation program.	Individual & family members	CHDSMP :booklet, a digital video disc (DVD), an individual face-to-face education session and 4telephone follow-ups.	N/A	88.4%

Study Setting	Country	Study Population	Age	Gender Male	Socio-economic & Education Status	Baseline SBP	Personnel Involved	Freq & Duration of patient/personnel training	Patients per group	Curriculum	Quality Assurance	Attendance Rates/ Intensity
Zhang, P 2018(24)												
hospital	China	236 patients with angina or MI	65.3 ± 8.1 vs 66.6 ±10.5	53.6%	High school education and above 46 (46.0) vs 38 (37.9)	N/A	Nurse	Patient education (7m+) Nurse-led transitional care programme.	Individual & group	Omaha system assessed four domains: environmental, psychosocial, physiological and health-related behaviours. Health education and handed out booklets of the health education contents.	N/A	84.3%
Wu Y 2019(25)												
Non-percutaneous coronary intervention hospitals	China	29, 346 acute coronary syndrome (ACS)	63.9 ± 11.72 vs 64.1±1 1.64	60.1%	High school and above 1616 (14.2%) vs 1815 (16.0%)	<90.0 mm Hg 357 (2.5) vs 391 (2.6)	Quality of care improvement (QCI) team, training clinical staff, senior cardiologists	Patient education (6m) Multifaceted QCI.	N/A	Quality improvement intervention: Hospital performance audit and feedback, implementation of a clinical pathway, training of physicians and nurses, online technical support and patient education.	The fidelity monitored at each site by the clinical associates from the study coordinating center. (Beginning, middle & end).	One hospital from wedge 2 and 1 hospital from wedge 4 dropped out in cycle 2.
Dalal, H 2007(11)												
home-based rehabilitation with hospital-based rehabilitation	United Kingdom	104 Acute MI	60.6 ±10.1 vs 64.3 ±11.2	10.8%	30 (51.0) vs 10 (26.0)	122.0 ± 21.0 vs 125.0 ± 21.0	Cardiac rehabilitation nurse, physiotherapist, or exercise therapist	Patient education (10w) Comprehensive cardiac rehabilitation with Heart Manual. Home visit followed up by telephone calls.	Individual or group	Comprehensive cardiac rehabilitation programme using a structured programme of exercise, stress management, and education.	N/A	85.6%
Ma, L. 2020(26)												
Hospital of medical university	China	300 patients who underwent coronary artery bypass grafting (CABG)	63.1 ± 9.7 vs 62.8 ± 10.7	78.7%	8.1 ± 3.0 vs 8.2 ± 3.1	Hypertension 98.0 (65.3) vs 91.0 (60.7)	Cardiologist, nursing specialist, dietitian, and psychologist	Patient education (12m) CAD-related health education (Once a week (2 months)), Exercise guidance and surveillance (once a month for 10 months), Risk factor control (once a month for 10 months), Psychological nursing (once a month for 10 months).	Individual	CAD-related health education lectures based on the CAD-related health education manual; individualized exercise plan; individualized risk-factor control strategies; Psychological nursing.	N/A	86.7%

Study Setting	Country	Study Population	Age	Gender Male	Socio-economic & Education Status	Baseline SBP	Personnel Involved	Freq & Duration of patient/personnel training	Patients per group	Curriculum	Quality Assurance	Attendance Rates/ Intensity
Santo, K. 2019(27)												
large urban tertiary hospital cardiology and cardiac rehabilitation outpatient clinics	Australia	163 patients with coronary heart disease (CHD)	58.4 ± 9.04 vs 56.8 ± 8.64	87.7%	N/A	125.9 (17.3) vs 124.4 (20.2)	Internist, peers	Patient education (3m) Medication reminder apps.	N/A	Advanced app (1) Daily reminders (2) Scheduled reminders (3) Medication refill reminders, adherence statistics, ability to export and share information with others.	N/A	93.9%
Wu, Q. 2019(28)												
cardiovascular inpatient departments of the Teaching Hospital	China	150 elderly patients with acute myocardial infarction undergoing PCI	70.2 ± 6.2 vs 68.6 ± 5.8	70.0%	Senior high school 11 (15.7%)vs 9 (12.9%)	127.5 (110.0-138.0) vs 130 (120.0-140.0)	Nurse	Patient education (3m) Transitional health management program. Handbook given to patients and their families after PCI and explained, repeatedly emphasizing the precautions during the transition period.	Individual	Maintaining a good lifestyle and self-management skills, drug guidance.	2 deputy chief physicians of CV medicine and 2 experienced health management professionals reviewed and revised.	93.3%
Ivers, N. M. 2020(7)												
Nine cardiac centres	Canada	2623 patients with obstructive coronary artery disease after a myocardial infarction	65.9 (12.1) vs 66.8 (12.5)	71.3%	N/A	N/A	Cardiac care nurse , health worker & hospital team	Patient education (44w) Delivered via booklets & phone call at 4, 8, 20, 32, and 44 weeks after myocardial infarction.	Individual	(1) Booklets: treatment; obtaining drug refills; daily adherence to medication; rehabilitation (2) Phone calls by an automated interactive voice response system one to two weeks after each mail-out.	N/A	64.2%
Huffman, M. D. 2018(12)												
63 hospitals	Southern Indian	21, 374 patients with acute myocardial infarction	60.9 (12.1) vs 60.3 (12.0)	75.7%	N/A	139.0 (28.9) vs 138.1 (29.1)	Quality improvement team	Patient education(4m) Quality improvement tool kit: 4-month steps over a 24-month period. Personnel Education Study team performed central and on-site training for 90 to 120 minutes at each site.	N/A	(1) Locally-developed standard admission & discharge order sets (2) Poster-size clinical pathways (3) Standardized patient lifestyle counselling (4) Electronic audit-feedback systems.	Monthly reporting , site-specific measures on performance comparing inter-hospital-level performance.	99.0%

Study Setting	Country	Study Population	Age	Gender Male	Socio-economic & Education Status	Baseline SBP	Personnel Involved	Freq & Duration of patient/personnel training	Patients per group	Curriculum	Quality Assurance	Attendance Rates/ Intensity
Chaves, GSS. 2019(29)												
Academic center Hospital	Brazil	115 Patients with CAD after myocardial infarction (MI)	60.7 ± 8.8 vs 58.7 ± 9.6	71.1%	Employed: 15 (40.5) vs 17 (43.6) High Education: 16(43.2) vs 11(28.2)	123.8 ± 15.1 vs 117.9 ± 17.6	Physiotherapists, internists, cardiologist, dietitian	Patient Education(6m) Individualised exercise prescription: 36 supervised sessions in decreasing frequency (3 times to 1/week and 1hour session). Additionally 24 education sessions, supported by a workbook for 30minutes.	Individual and groups	Exercise program: Individualized exercise prescription based on a graded exercise stress test.	N/A	80.9%
Zhang, P. 2017(30)												
1700-bedded top level general hospital	China	236 patients with angina or myocardial infarction	66.6 (10.5) vs 65.3 (8.130)	53.8%	High school education and above 38(37.9) vs 46(46.0)	146.1 (17.7) vs 149.5 (14.0)	Nurse	Patient education(7m) 2 phases, pre-discharge phase (about 1 week before discharge) and post-discharge phase (7 months).	Individual & Group	Nurse-led transitional care program: (1) Teaching and counselling scheme (2) Treatment and procedures scheme (3) Case Management scheme (4) Surveillance scheme	Benefits and barriers of health promoting behaviours and perceived self-efficacy were tested from patients.	86.0%
Lin, C. Y. 2017(31)												
Multiple centers	Iran	288 older patients undergoing CABG surgery	75.0 (69.0 - 79.0) vs 76.0 (70.0-80.0)	66.4%	Household income 893.1 (942.9-1286.9) vs 848.7 (453.4-1295.4) Education years 4.0 (1.0-12.0) vs 4.0 (1.0-12.0)	N/A	Nurse, cardiologists, psychologists	Patient education (5w) Psycho-Education for 3 weekly session and each session lasted for 1 hour. 5 weekly sessions of MI that each lasted around 50 minutes.	Individual	(1) Psycho-education: information about CAD and ways of coping with the disease. Medications information& non-adherence, and communication between the family members. (2)Motivational Interviewing (3) Reminders via SMS	N/A	87.9%
Wang, W. 2016(32)												
Cardiology clinics of a tertiary public hospital	Singapore	128 Patients with MI	54.9 (8.7) vs 55.8 (10.3)	89.9%	Secondary and above 55 (86.0%) vs 46 (71.9%)	127.9 (21.8) vs 134.8 (20.0)	Trained RA	Patient education (4w) Myocardial Infarction Home-based Self-management Programme: 40-minute F2F education session.	Individual & family members	Psychologically-related content including relaxation techniques, stress management and CHD symptom-monitoring.	N/A	91.4%

Study Setting	Country	Study Population	Age	Gender Male	Socio-economic & Education Status	Baseline SBP	Personnel Involved	Freq & Duration of patient/personnel training	Patients per group	Curriculum	Quality Assurance	Attendance Rates/ Intensity
Alsaleh, E. 2016(33)												
2 hospitals	United Kingdom	156 Patients with coronary heart disease	57.7 ± 10.5 vs 58.0 ± 8.7	54.2%	Retired 17 (23.9) vs 19 (22.4) Secondary and above 40(56.3%)vs 54(63.5%)	134.9 (17.6) vs 138.8 (18.2)	Cardiac nurse, Internist	Patient education(6m) Initial F2F consultation lasting 20-30 minutes & 6 telephone call-based consultations (15-20 minutes each, 1/month). Personnel Education Prior expertise in delivering health education and interventions using motivational interviewing techniques.	Individual & group	Patient Behavioural change, goal-setting, self-monitoring, feedback.	Intervention was delivered consistently (with respect to length and duration of contacts and extent of advice given), and ensured intervention fidelity.	93.0%
Mosca, L 2010(13)												
Medical centers	United States	304 Women hospitalized with CHD coronary heart disease (CHD)	59.0 (39.0) vs 67.0 (44.0)	0.0%	Employed 39(26.0) vs 35(23.0) >High school education 73(49) vs 61(40)	<140/90mmHg: n(%) 114 (75.0) vs 118 (77.0)	Prevention facilitator, internist	Patient education(6m) Initial educational session included 1 hour of structured counselling before discharge and during phone visits at 2, 4, and 12 weeks and a phone or clinic visit at 6 weeks post discharge.	Individual	Cardiac rehabilitation: Heart rate guidelines and Borg perceived exertion levels Lifestyle modification Educational booklet : rationale for risk factor modification and strategies to attain each goal.	N/A	77.6%
Skobel, E. 2017(34)												
Cardiac rehabilitation	Spain, Great Britain_and _Germany	118 Coronary artery disease	60.0 (50.0-65.0) vs 58.0 (52.0-67.0)	89.0%	N/A	129.0 (120.0-140.0) vs 130.0 (113.0-140.0)	Sport physicians and exercise scientists	Patient education(6m) Smartphone-guided training (GEx) system.	Individual	Disease management system facilitating prescription and administration of CR therapies made up of 3 main components: Professional System (Web-based tool), Patient Station and Portable Station.	N/A	67.0%
Jørstad, H. T. 2016(17)												
11 hospitals	Netherlands	754 patients with symptomatic coronary artery disease	57.7(9.5) vs 58.2(9.7)	79.0%	Employed 162 (53.0 %) vs 171 (56.0 %) Higher education (>8 years) 69(22.0 %)vs 65(21.0 %)	N/A	Nurse, cardiologists	Patient Education(6m) Nurse-coordinated prevention programme: 4 outpatient clinic visits. Personnel Education Received at least 1 day of central training in CV risk management and 1 day of local individual training.	Individual	(1) Healthy lifestyles (2) Biometric risk factors (3) Medication adherence	Individual nurses observed on at least twice. Video of nurses' consultations evaluated by a medical psychologist.	94.2%

Study Setting	Country	Study Population	Age	Gender Male	Socio-economic & Education Status	Baseline SBP	Personnel Involved	Freq & Duration of patient/personnel training	Patients per group	Curriculum	Quality Assurance	Attendance Rates/ Intensity
Melamed, R. J. 2014(35)												
Hospital	Germany	395 patients with coronary heart disease (CHD)	65.7 vs 65.8	79.4%	Education>10 years and above 110(56.4%) vs 112(56.3%)	N/A	Primary care physicians, cardiologists, medical assistants	Patient education(3m) Brochure- independent study and for repeating the previous module. Patients were able to comments and responses to questions (work-book). Exercise diary to enable them to document their daily physical activity.	Individual	(1) A patient brochure (2)Teaching cards (3)A curriculum (4)A poster/wall chart set.	N/A	90.2%
Vaillant, A. 2015(36)												
2 Intensive Coronary Care Units (ICCU)	France	183 coronary artery disease (CAD)	66.0 ±14.0 vs 65.0 ± 15.0	69.0%	N/A	136.0 ±14.0 vs 137.0 ±16.0	Nurse, clinical research assistant	Patient education(4m) an Individual interview that lasted approximately 30 minutes.	Individual	Log book content (1)personalized advice for healthy lifestyle control (2) CVRF evolution, including monthly weight and abdominal perimeter watching, and blood pressure monitoring.	N/A	91.5%
Seidl, H. 2015(37)												
Hospital	Germany	340 elderly patients with AMI	75.2 ± 6.0 vs 75.6 ± 5.9	62.0%	N/A	N/A	Nurse	Patient education(24m) At least 1 home visit and quarterly telephone calls in the first year, and semi-annual calls in the following 2 years. Personnel Education Nurses received a training on assessments and interventions by the study physician and the principle investigator.	N/A	Information booklet: disease and comorbidities, about medication and with behavioural recommendations.	Regular meetings among team members and study nurses to discuss problems, assessment &intervention and for standardization.	79.1%
O'Neil, A. 2015(38)												
6 Hospitals	Australia	121 Acute coronary syndrome	60.0	75.3%	Retired 25(41.0)vs 14(23.3)High school and above 100%	N/A	Psychologists	Patient education(6m) MOODCARE- Telephone-based structured intervention sessions (consisted of short- and long-term goal setting comprised 10 sessions) over 6-months.	N/A	Motivational interviewing, goal setting, behavioural activation, cognitive restructuring & supplementary handbook.	All intervention telephone sessions were audio-taped and 17% were reviewed by a senior clinical psychiatric consultant.	75.2%

Study Setting	Country	Study Population	Age	Gender Male	Socio-economic & Education Status	Baseline SBP	Personnel Involved	Freq & Duration of patient/personnel training	Patients per group	Curriculum	Quality Assurance	Attendance Rates/ Intensity
Holmes-Rovner, M. 2008(39)												
5 community hospitals	United States	525 patients with acute coronary syndrome (ACS)	59.0(12.0)vs 60.5(11.9)	36.5%	12.5 (2.3) vs 12.6 (2.3) years	N/A	Health educator	Patient education(6w)<br>6 session health behaviour change telephone counselling program.	Individual & family members	Behaviour change strategies: behavioural staging, motivational interviewing, goal setting, relapse prevention, and obtaining social support. Each patient and family received an information booklet and goal worksheets.	N/A	73.9%
Berwanger, O 2012(2)												
34 clusters public hospitals	Brazil	1150 patients with acute coronary syndromes	62.0 ±13 vs 62.0 ±13	68.6%	N/A	Hypertension 433 (71.9) vs 402 (73.4)	Nurse, internist	Personnel Education (8m)<br>Educational materials for clinicians, reminders, algorithms, and case manager training.	N/A	(1)Web-based and telephone training.<br>(2) Workshop for additionally 2 health professionals.<br>(3) Checklist of risk stratification & recommended therapies for each risk category.<br>(4) Educational materials.	N/A	97.9%
Lapointe, F 2006(18)												
2 hospitals	Canada	127 Patients with MI	57.8 ± 9.6 vs 56.9 ± 8.8	83.4%	Unemployed 27 (39.7) vs 35 (53.8)<br>Secondary And above 47(69.2) vs 40(61.6)	N/A	Nurse, internists, dietician, pharmacist	Patient Education (12m)<br>Systematic follow-up (by telephone or in person).	Individual	Importance of cholesterol management in the prevention of CAD, and the need for the long-term follow-up and treatment of this risk factor.	N/A	87.4%
Wang W 2012(40)												
2 university-affiliated public general hospitals	China	160 Patients with MI	57.3 (8.6) vs 58.3 (10.4)	83.4%	Secondary Education 47(69.2)vs 40(61.6)	N/A	Principal researcher	Patient Education(6w)<br>Home-based rehabilitation program -1 hour introduction to the manual, explaining its purpose and content, providing instruction in its use, and demonstrating tai chi skills + 30minutes consultation.	Individual & family members	Heart manual:<br>(1)6 weekly topics on health education.<br>(2)Frequently asked questions about medication, PCI, and anxiety and depression after myocardial infarction.<br>(3) Biomedical profiles.	Integrity ensured via well-explained information materials, family members, telephone reinforcement<br>.	85.0% vs 81.2%

Study Setting	Country	Study Population	Age	Gender Male	Socio-economic & Education Status	Baseline SBP	Personnel Involved	Freq & Duration of patient/personnel training	Patients per group	Curriculum	Quality Assurance	Attendance Rates/ Intensity
Hawkes, A. L 2013(41)												
2 large metropolitan hospitals	Australia	430 patients with coronary heart disease (CHD)	61.3 (11.3) vs 59.9 (11.1)	74.7%	Employed 94 (45.0%) vs 101 (48.1%) High school 168 (80.4) vs 177 (83.1)	118.0 (15.8) vs 118.1 (17.4)	Health professional /'health coach'	Patient Education(6m) 10 × 30 minutes scripted telephone health coaching sessions. Personnel Education Web-based computer application and key enter all session information.	Individual	Health coaching sessions: (1) Reduction of clinical risk factors (2) Modification of behavioural risk factors (3) Compliance with medication (4) Management of psychosocial issues.	All intervention calls are audio-taped with a proportion reviewed to ensure delivery of intervention protocol.	75.6%
Young, W 2003(42)												
407-bed acute care hospital	Canada	162 patients recovering from MI	67.8 (13.1) vs 70.1 (13.4)	59.6%	Retired 53 (74.6) vs 55 (73.3) <=Grade8 22 (31.0) vs 23 (30.7)	N/A	Multidisciplinary teams-Cardiac-trained nurse, family physician	Patient Education(12m) 6 home visits.	Individual	Standardized nurses' checklist, referral criteria for specialty care, communication with the family physician and patient education.	N/A	90.1%
Chair SY 2013(43)												
hospital cardiac rehabilitation centre	Hong Kong	146 patients with coronary heart disease	66.8 (10.3) vs 66.0 (11.2)	68.5%	Retired 45 (61.6%) vs 40 (54.8%) Secondary Education 35(49.3%) vs 38(52%)	129.1 (16.1) vs 129.5 (19.6)	Nurse, dietitian	Patient Education(6m) 16 supervised exercise sessions (2hours per session, twice a week) Week 1-8: 6hour group education sessions (1 hour per session per week) and one individualized education session with a dietitian. 9w-6m: 1 supervised exercise session per week (2 hours/session).	Individual and groups	(1)Education Session: diet, exercise, smoking cessation, stress management and drug compliance (2)Motivational interviewing was tailored to the patient's stage of change. Importance of their roles in managing the disease and planning rehabilitation.	3 research members with motivational interviewing expertise supervised motivational interviewing sessions. 15 patients selected for audit of the intervention.	79.5%
Carroll, D. L 2007(44)												
5 academic medical centers	United State	247 adults who had a diagnosis of MI or CABS	76.4 ± 6.4 vs 76.2 ± 6.2	32.5%	Employed 27(22.0)vs 18 (14.0)Secondary Education 98/121(81.0) vs 100/125(80.0)	Hypertension 93.0 (77.0) vs 96.0 (76.0)	Nurse, peer advisor	Patient Education(12w) Social support and self-efficacy enhancement interventions- home visit and contacted the subjects over the telephone at least 3 times. Peer adviser telephone at least once a week.	Individual	Verbal encouragement and support, active listening, sharing by the peer advisors of their experiences, reinterpretation of symptoms, exercise promotion, energy management, teaching about the cardiac disease process.	N/A	81.4%

Study Setting	Country	Study Population	Age	Gender Male	Socio-economic & Education Status	Baseline SBP	Personnel Involved	Freq & Duration of patient/personnel training	Patients per group	Curriculum	Quality Assurance	Attendance Rates/ Intensity
Hanssen, T. A 2009(45)												
University Hospital	Norway	288 adults who had a diagnosis of MI or CABS	59·5 (12·9) vs 60·9 (10·8)	80.6%	Unemployed77/ 153 (50.3)vs 59/129 (45.7) Secondary Education 58/104(55.8) vs 79/121(65.3)	Hypertension 44/156 (28.2) vs 41/131 (31.3)	Nurse, general practitioner, cardiologist	Patient Education(6m) Telephone follow-up intervention (TFI): telephone calls were arranged for the first four weeks; subsequently calls were arranged six, eight, 12 and 24 weeks after discharge.	Individual	Individual needs and support of patients' own coping efforts with respect to lifestyle changes and risk factor reduction.	N/A	81.4%
Tingström PR 2005(46)												
2 hospitals	Sweden	207 patients with coronary artery disease	59.1 ± 7.1 vs 59.4 ± 7.2	73.9%	Secondary Education 93/136(68.4) vs 53/103(51.46)	N/A	Rehabilitation team member-tutor	Patient Education(12m) 13 occasions for one and a half hours. They met weekly in 1st month and then every other week during the next month.	Groups	Structured problem solving process to stimulate self-directed learning(diary) via real-life situations: CAD and symptoms, psychological reactions, risk factors and drug treatment.	N/A	97.0%
Zhao Y 2009(47)												
2 largest comprehensive hospitals	China	200 patients with newly diagnosed with coronary heart disease	72.9 ± 6.4 vs 71.6 ± 4.14	49.0%	Secondary Education 49 (49.0) vs 38 (38.0)	N/A	Nurse, cardiac physicians	Patient Education(12w) Transitional care programme (TCP): predischarge assessment, structured home visits and telephone follow-ups within 4 weeks after discharge. Personnel Education Health teaching and counselling, treatment and procedures, case management and surveillance.	Individual	Adherence behaviours with regard to diet, medications, exercise and health-related lifestyle.	Research team randomly chose 10% cases to visits with Nurse-C and reviewed the telephone calls to ensure compliance with protocol.	90.9%
Mayer-Berger W 2014(3)												
Hospital	Germany	600 patients with coronary artery disease	49.1 ± 5.4 vs 49.2 ± 5.7	89.2%	>13years 8(3.0) vs 5(1.5)	N/A	Physiotherapist, Internists	Patient Education(36m) 3weeks in-patient CR plus additional rehabilitation at 6 months. Telephone consultations over a period of 36 months.	Individual & Group	Physical training and resistance exercise. Group sessions (risk factor management, coping strategies, diabetes, and smoking cessation) and individual counselling in fitness level.	N/A	71.7%

Study Setting	Country	Study Population	Age	Gender Male	Socio-economic & Education Status	Baseline SBP	Personnel Involved	Freq & Duration of patient/personnel training	Patients per group	Curriculum	Quality Assurance	Attendance Rates/ Intensity
Du L 2016(48)												
University Hospital	China	979 acute coronary syndrome and underwent successful PCI	60.4 ±11.0 vs 61.6 ±11.2	72.7%	N/A	127.0 ± 22.8 vs 127.0 ± 27.9	Research nurses and internists	Patient Education(36m)<br>Weekly educational lectures: Telephone calls from cardiologists at 1,2,3,6, 12 and 36 months and medical consultations from cardiologists. F2Fvisits necessary.<br>Personnel Education<br>Received special training for this study.	Individual	Educational lectures focusing on the updated guidelines on secondary prevention after PCI. Health education, disease-prevention suggestions and consultations on medication usage.	Quality controls were performed in randomly selected patients once every three months.	98.5%
Yu CM 2004(49)												
University-affiliated outpatient cardiac rehabilitation and prevention center	China	269 patients with recent AMI/ after elective PCI	64.0 ±11.0 vs 64.0 ±11.0	75.5%	N/A	Hypertension 84 (46.0) vs 37 (43.0)	Physiotherapist, occupational therapist, cardiologist	Patient Education(24m)<br>Phase 2: 2 times weekly outpatient education and exercise program (8weeks).<br>Phase 3: community-based home exercise program lasting 6 months.	N/A	Prevention and treatment of coronary heart disease and risk factor modification.	N/A	75.8%
Murphy BM 2013(50)												
2 hospitals	Australia	275 post-AMI or those who underwent CABG/PCI	58.0 ± 8.9 vs 59.9 ± 9.3	86.5%	N/A	Hypertension 87 (73.7) vs 84 (77.8)	Nurse, psychologists	Patient Education(8w)<br>"Beating Heart Problems" program: F2F group program comprising 8 weekly sessions of 1.5 hours each.	Groups	Modules address PA, diet, medication adherence, smoking cessation, psychosocial support. Within each module, review situations in their lives to identify, challenge, and change thoughts and beliefs with risk factors and negative emotions.	Program developers facilitated the sessions and supervised cofacilitators. A practitioner manual was used to further ensure standardized approach.	77.5%
Katalinic A 2008(51)												
11 hospitals	Germany	1500 patients with coronary artery disease	62.0 ± 10.0 vs 63.0 ± 10.0	77.0%	N/A	Hypertension 134 (78.0) vs 451 (78.0)	Internists	Patient Education(12m)<br>Trans-telephonic ECG	Individual	Trained to use the device and send the ECG by telephone to the call centre. If patients had symptoms, they could contact the call centre, transmit ECG (without re-dialling) and consult a physician.	N/A	100.0%

Study Setting	Country	Study Population	Age	Gender Male	Socio-economic & Education Status	Baseline SBP	Personnel Involved	Freq & Duration of patient/personnel training	Patients per group	Curriculum	Quality Assurance	Attendance Rates/ Intensity
Briffa TG 2005(52)												
2 tertiary hospitals	Australia	113 patients survived from uncomplicated AMI or recovery from unstable angina	61.9 ± 9.4 vs 60.8 ± 8.7	73.6%	N/A	Hypertension 25 vs 29	Clinical nurse consultant, physiotherapist, clinical psychologist, dietitian, social worker or pharmacist	Patient Education(6w) 3 times weekly sessions, each comprising 60-90 minutes of supervised exercise, combined with 45 minutes of education (12 occasions) and 45 minutes of psychosocial counselling (6 occasions).	Groups individual	Aerobic circuit training interspaced with resistance training was the focus of the rehabilitation programs at both participating institutions. Symptom management, pharmacological treatment, healthy eating, psychosocial counselling and stress management.	N/A	94.7%
Sinclair AJ 2005(53)												
3 district general hospitals	United Kingdom	324 MI patients	74.2 ± 6.0 vs 73.8 ± 6.6	57.0%	N/A	N/A	Nurse	Patient Education(8w) At least 2 home visits after hospital discharge. 1-2 and 6-8 weeks after discharge. Extra visits and telephone contacts were permissible if there is specific need and individualised booklet. Personnel Education Trained in cardiac support.	Individual	General information about safe levels of activity and a simple 6-week graded exercise programme, details of personal risk factors, useful telephone numbers and advice on when to seek medical advice and how to manage symptoms.	N/A	91.4%
Vale MJ 2003(54)												
6 university teaching hospital	Australia	792 patients with coronary artery disease	58.6 (10.6) vs 58.3 (10.6)	77.0%	N/A	130.3 (18.1) vs 129.9 (19.5)	Dietitian, nurses	Patient Education(6m) Coach program: via telephone & written reports. Report were mailed for reference and reinforcement of expected progress by the next coaching session. Personnel Education(2w) Part-time training programme in conducting, techniques, and software package.	Individual	Physician clinic visits and measurement of their cardiovascular risk factors and to be informed of the results of these measurements, education regarding targets, plan of action to achieve the target, and monitoring of the patient's progress toward target achievement.	N/A	85.7%

Study Setting	Country	Study Population	Age	Gender Male	Socio-economic & Education Status	Baseline SBP	Personnel Involved	Freq & Duration of patient/personnel training	Patients per group	Curriculum	Quality Assurance	Attendance Rates/ Intensity
Arthur HM 2000(55)												
7 cardiovascular surgeons operating surgical center	Canada	246 patients on a waiting list for elective CABG	61.8 ± 8.4 vs 63.8 ± 7.8	85.4%	Unemployed/retired 79.7 vs 84.7% Education 12.2y vs 11.1y	132.0 vs 136.0	Nurse, primary care physicians, cardiologists, surgeons, kinesiologists and exercise specialists	Patient Education(8w) Prescribed exercise training 2 times/ week in a supervised environment; education and reinforcement; and monthly nurse-initiated telephone calls.	Individual & Group	Supportive–educative component: detailed preoperative teaching + monthly telephone. Educational content: standardized information about CV risk factors(videotaped and written).Patients can ask questions, psychological issues. And referred to the psychologist if necessary.	N/A	84.6%
Goodman H 2007(56)												
Hospital	United Kingdom	188 Patients waiting for coronary artery bypass surgery	63.7 vs 65.9	81.3%	N/A	149.1 (23.9) vs 141.5 (19.6)	Nurse	Patient Education(3m) Copy of the manual + telephone (between visits). Personnel Education Lifestyle counselling and preparation for surgery every monthly.	Individual	Risk factors, lifestyle change, preparation for surgery and what to do if they encounter chest pain.	N/A	94.2%
Jolly K 1999(57)												
67 practices	United Kingdom	597 Post-MI or angina patients	63.0 (10.0) vs 64.0 (10.0)	71.0%	N/A	128 (19.0) vs 129 (21.0)	Specialist cardiac liaison nurses	Personnel Education(6m) Liaison nurse telephoned the practice shortly before patients discharged. Practice nurses were encouraged to telephone back.	N/A	Liaison Nurse: Care of each patient and to book the first FU visit to the practice. Practise Nurse: (1)Discuss problems or seek advice-clinical or organisational issues. (2)Evidence based guidance on clinical management (3) Initial training on behavioural change and an ongoing support group for information needs.	N/A	90.5%

Study Setting	Country	Study Population	Age	Gender Male	Socio-economic & Education Status	Baseline SBP	Personnel Involved	Freq & Duration of patient/personnel training	Patients per group	Curriculum	Quality Assurance	Attendance Rates/ Intensity
Redfern J 2008(15)												
Tertiary referral hospital	Australia	144 patients with acute coronary syndrome	62.0 (1.6) vs 67.0 (1.3)	74.5%	Employed: 35 (49.0) vs 16 (22.0)	>140 mm Hg: 27 (38.0) vs 35 (49.0)	Family doctors, cardiologist	Patient Education(3m) 1 hour initial consultation and multiple follow-up phone call. Rehabilitation included two 60 min gym sessions and a 2 h education session weekly for 6 weeks. Doctor-directed, hospital programme , individual or self-help.	Individual	Stage 1 : Modules set up and leaflets Stage 2: F2F risk factor assessment Stage 3: Risk factors to lower, goal-setting Stage 4: Comprised telephone FU during which each patient's risk factor(s) goals and strategies were re-evaluated. Changed if necessary.	N/A	94.4%
Brügemann J 2006(58)												
1 cardiac rehabilitation centre	Netherlands	137 patients with coronary revascularisation procedure	57.0 (7.7) vs 57.0 (7.7)	100.0%	N/A	Hypertension 51(37.0)	Nurse, dietitian, psychologist	Patient Education (8w) 2 h teaching session, PA of at least 30 min (3/weeks, for 6w). Relaxation therapy and weekly psychoeducation sessions.	Group	(1)Heart function and risk factor management. (2) Physical training. (3)Theory of rational emotive behaviour therapy.	N/A	86.9%
Williams JB 2011(4)												
458 hospitals	United States	458 patients undergoing isolated coronary artery bypass grafting	63.7 ± 1.8 vs 63.8 ± 1.9	75.0%	N/A	Hypertension 74.8 ± 6.4 vs 75.3 ± 6.7	Cardiac surgeons	Patient Education (2y). Discharge "flight plan" checklist Personnel Education(2y) CQI : received educational information on discharged medications prescriptions . Standardized care orders, reminders and periodic newsletters.	N/A	Patients: Importance of secondary prevention medications and other lifestyle modification interventions. Discharge "flight plan" checklist of evidence-based discharge medications to be considered.	Site-specific feedback reports (use of these 4 pharmacological agents)every 6m.	100.0%
Carlsson R 1998(19)												
1 hospital	Sweden	141 AMI/ patients who undergone coronary artery bypass grafting	62.5±5.3 vs 60.8 ± 5.4	80.3%	N/A	Hypertension 47 vs 48	Nurse, nurse rehabilitator, cardiologist	Patient Education(9m) Counselling for 9 hours per patient during the first year. Continuous physical exercise programme 2-3 times weekly for a period of 2-3 months. Individual exercise schedules were provided.	Individual & Group	CAD risk factors and the effect of lifestyle changes on the prognosis. Smoking cessation (1.5 hours); dietary management (5.5 hours); physical activity (2 hours).	N/A	97.0%

Study Setting	Country	Study Population	Age	Gender Male	Socio-economic & Education Status	Baseline SBP	Personnel Involved	Freq & Duration of patient/personnel training	Patients per group	Curriculum	Quality Assurance	Attendance Rates/ Intensity
Capone RJ 1988(59)												
3 hospitals	United States	1004 patients with AMI	56.5 vs 56.2	77.4%	N/A	Hypertension 44.3 vs 48.4	Nurse, internists	Patient Education(12m) Telephone contact with a trans-telephonic ECG transmitter (l-minute ECG rhythm transmission-made prior to discharge, 3 times first week after discharge, weekly for 1 month, semi-monthly for 1 month, and then monthly up to 1 year following MI.	Individual	(1)Instruction program focus on acute or changing cardiac symptoms and the immediate medical care. (2) Routine telephone FU program for new symptoms and to reinforce immediate medical contact. (3) Cardiac emergency protocol.	Use of the emergency telephone system (trained nurse to evaluate).	100.0%
Rollman BL 2009(60)												
7university-based and community hospitals	United States	453 post-CABG patients	64±10. 8 vs 64 ±11.2	88.5%	> high school 57 (86) vs 54 (82)	Hypertension 87 (131.0) vs 80 (122.0)	Nurse, psychologist, psychiatrist	Patient Education(8m). Telephoned patients every other week to review lesson plans, monitor antidepressant, administer the PHQ-9 to assess treatment response, encourage follow-up. Telephone contacts lasted 15 to 45 minutes and continue for 2-4 months.	Individual	(1)Basic psychoeducation (workbook- self-care for depression). (2)Initiation or adjustment of antidepressant; watchful waiting for mildly elevated mood symptoms; or referral to a psychologist or psychiatrist.	N/A	87.6%
Kripalani, S 2012(61)												
primary care clinics	United States	435 patients with coronary heart disease	62.8 ±10.0 vs 63.7 ±9.3	44.4%	11.1 ± 2.6y vs 10.9± 3.2y	Hypertension 114 (98.3)vs 96 (100.0)	Pharmacist	Patient Education(12m) Illustrated daily medication schedules: 5 minutes. Refill reminder postcards: mailed to their home around 25 days after their last medication fill, as a behavioural cue.	Individual	Orientation to the tool and review of medications. Universal Medication Schedule: name, indication, and dosage instructions, purposes. Postcard displayed the refill date & important reminders.	N/A	96.6%
Tranmer JE 2004(62)												
Hospital	Canada	200 patients underwent CABG and/or valve repair	63.8 (38.7 - 87.1) vs 66.61 (41.9- 82.6)	76.1%	N/A	65 (63.7%) vs 58 (59.2%)	Nurse	Patient Education(5w) Active and ongoing FU via telephone calls 3 and 5 days following hospital discharge, then weekly for 4 more weeks (20-30minutes).	Individual	Provided ongoing information and assessment, assisted with self-management of common symptoms, and facilitated referrals to appropriate health care resources.	N/A	92.0%

Study Setting	Country	Study Population	Age	Gender Male	Socio-economic & Education Status	Baseline SBP	Personnel Involved	Freq & Duration of patient/personnel training	Patients per group	Curriculum	Quality Assurance	Attendance Rates/ Intensity
Munoz MA 2007(8)												
23-centre	Spain	983 patients suffered MI or angina	64.2 (9.8) vs 63.6 (10.3)	74.7%	low social class 79.3 vs 77.7	130 (17.0) vs 134 (17.0)	General practitioners (GP)	Patient Education(3y) Mail reminder quarterly to consult with their GP + contacted on two occasions by telephone or mail to obtain clinical information. Personnel Education Instructed to strictly follow the updated guidelines on cardiovascular disease prevention + copy of the study protocol.	Individual	Healthy lifestyle, including materials about the traditional Mediterranean diet , physical exercise (GPs offered various options, such as walking and cycling), hypo-caloric diet, and counselling to quit smoking if applicable.	N/A	79.9%
Heller, R. F 1993(63)												
5 major hospitals	Australia	450 post AMI patients	59.0 (8.0) vs 58.0 (8.0)	72.0%	employed 26.0% vs 28.0 % high school/ higher education 26 .0 vs 32.0	Hypertension 53%	Family doctors	Patient Education(6m) Mail-out intervention program, contact with subjects was maintained over the next 4 months with monthly newsletters + 2 supplementary telephone contact Personnel Education Letter was sent to the family doctor regarding the benefit of aspirin and beta blockers for secondary prevention.	Individual	First of 3 mail-out packages: introduction and Step 1 ("Facts on Fat" kit plus walking programme and information on the "Quit for Life" program for smokers), Step 2 (takeaways, snack food sand extras), Step 3 (meat fats) of the "Facts of Fat" kit. , Step 4 (dairy food) and Step 5 ("putting it all together").	N/A	85.3%
Bolman C 2005(64)												
Hospital	Netherlands	153 patients with coronary artery disease	61.9 (10.5)	81.0%	secondary school and above 50.4%	N/A	Cardiologists	Patient Education(10m) Checklist with the accompanying written instructions mailed to their home address, approximately 1 week before their outpatient visit. Average of 1 (T1), 4 (T2) and 10 months after discharge (T3).	Individual	Frequently asked questions checklist : Checklist only included questions-intended to help patients prepare for a visit to their cardiologist, and it was suggested that patients should complete it at home before the visit.	Process evaluation given to the respondents right after the visit to the cardiologist.	78.5%

Study Setting	Country	Study Population	Age	Gender Male	Socio-economic & Education Status	Baseline SBP	Personnel Involved	Freq & Duration of patient/personnel training	Patients per group	Curriculum	Quality Assurance	Attendance Rates/ Intensity
Furze G 2008(65)												
1tertiary centre	United Kingdom	204 Patients waiting for CABG	64.3 ± 8.8 vs 65.3± 8.5	80.5%	N/A	145.0 ± 20.0 vs 145.0 ±21.0	Nurse	Patient Education(6w)<br>10–15 minute phone calls to their home at weeks 1, 3 and 6 (+/−1 week) and then monthly until they were admitted for their operation. HeartOp Programme: booklet + relaxation programme on audiotape or CD and a diary.	Individual	2 part patient-held booklet (the HeartOp Plan) : cardiac myths and misconceptions, reducing risk factors for secondary prevention, and what to expect during the hospital stay and subsequent recovery period.	N/A	89.3%
Dracup K 2009(66)												
6 sites	United States Australia, New Zealand	3522 patients with ischemic heart disease	<65y: 690 (38.8%) vs 662 (38.0%)	68.0%	Unemployed 1272 (71.8) vs 1245 (71.4) High school and above 100%	Hypertension 986 (56.3%) vs 958 (55.5%)	Nurse	Patient Education(1m)<br>Received education and counselling about ACS symptoms and actions required.1 month post-intervention session, the nurse called each patient and reviewed the main points from the initial session. The average length of the phone call was 15 minutes.	N/A	(1) Standardized information about symptoms of ACS (2)Emotional component: emotional responses to ACS symptoms leading to delay, to discuss their previous experiences accessing the medical system. (3) Social factors: spouse, another family member, or friend to the intervention session if possible.	N/A	91.7%
Keeping-Burke L 2013(67)												
Hospital	Canada	182 dyads of CABG surgery patients and their caregivers	63.6 ± 9.7vs 63.9 ± 8.8	85.7%	11.2 ± 3.8 vs 11.8 ± 3.6	N/A	Multidisciplinary health care team(nurse and physiotherapist)	Patient Education(10m)<br>VITAL program (telehealth): 30-minute information sessions. Caregivers received 2 hours instructional sessions- operation of the home monitoring equipment.	N/A	Booklet: step-by-step set up of the telehealth unit. 1st session: gave instructions to contact the telehealth center. 2nd session: caregiver set up the equipment and how to connect the monitor for the electrocardiogram (EKG), blood pressure, and oxygen saturation level.	Discharge and medication instructions were reviewed.	91.2%

Study Setting	Country	Study Population	Age	Gender Male	Socio-economic & Education Status	Baseline SBP	Personnel Involved	Freq & Duration of patient/personnel training	Patients per group	Curriculum	Quality Assurance	Attendance Rates/ Intensity
Yu C 2020(14)												
5 teaching hospitals	China	1000 patients who underwent an isolated CABG	57.4± 9.0 vs 57.1± 9.2	85.5%	Higher 444/501 (88.6) vs 422/499 (84.6)	124.8 ± 15.3 vs 124.9 ± 14.0	Nurse, internists	Patient Education(6m). Heart Health Application- smartphone application: demonstrated the operation of using the four modules	Individual	Medication reminders, cardiac health education, health questionnaire and feedback, and a personal data centre.	N/A	99.0%
Varnfield M 2014(68)												
4 cardiac rehabilitation centres	Australia	120 post MI patients	54.9 ± 9.6 vs 56.2 ± 10.1	87.0%	N/A	127.5±18.9 vs 124.9±16.1	CR centre clinician	Patient Education(6w) Received detailed programme information and 1 hour of F2F training on technology use. Provided weekly scheduled telephone consultations (~15 min each) over 6 weeks. Synchronised smartphone data via their mobile 3G network to a web portal.	Individual	Platform using smartphone for health and exercise monitoring, and delivery of motivational and educational materials to participants via text messages and preinstalled audio and video files (including understanding cardiovascular disease (CVD), symptoms and management).	N/A	60.0%
Leemrijse, C. J 2016(69)												
5 Dutch hospitals	Netherlands	374 patients with coronary diseases	61.0 (10.0%) vs 60.0 (10.4%)	80.9%	Higher education 34.8% vs 32.5%	130.0 ± 17.2 vs 132.0 ± 19.3	Nurse	Patient Education(6m) Coach(nurse) contacted each patient every 4 to 6 weeks by telephone. Each session lasts about 20minutes with a clear agreement regarding date and time of the next call. Personnel Education Courses on motivational interviewing.	Individual	(1) Information on individual risk factors and targets; (2)Information and education on methods to attain target (3) Plan to reach those targets; (4) Stimulating healthy dietary and exercise behaviour; (5) Monitoring and feedback; (6) Patient responsibility; (7) Stimulating assertiveness to health care providers and the patient's direct environment.	N/A	88.5%

Study Setting	Country	Study Population	Age	Gender Male	Socio-economic & Education Status	Baseline SBP	Personnel Involved	Freq & Duration of patient/personnel training	Patients per group	Curriculum	Quality Assurance	Attendance Rates/ Intensity
MEDMAN 2007(5)												
9 sites primary care organizations	United Kingdom	1493 patients with coronary heart disease	68.7 ± 9.2 vs 68.8 ± 9.1	69.0%	N/A	138.8 ± 18.9 vs 138.6 ± 20.5	Community pharmacists, GP	Patient Education(10m) Medicines management service :Initial consultation by the extracted medical data. Further consultations according to pharmacist. Personnel Education Received training designed and delivered by the Centre for Pharmacy PG Education.	N/A	Assessments: therapy, medication compliance, lifestyle and social support.	Intervention patients were also asked about their experience of the medicine management service.	90.8%
Ho, P. M 2013(70)												
4 Department of Veterans Affairs medical centers	United States	253 patients with cute coronary syndrome	63.8 ± 9.3 vs 64.0 ± 8.6	98.0%	N/A	Hypertension 113 (92.6) vs 106 (89.1)	Pharmacist	Patient Education(1y) Occurred at the 1-week and 1-month visit following discharge. During months 2-6, patients received both medication reminder (monthly) and medication refill calls for the 4 medications. During months 7-12 only received medication refill calls.	N/A	(1)Medication Reconciliation and Tailoring. (2)Educational messages provided through automated voice messages and pharmacist telephone. (3) Voice Messaging (medication reminder and refill calls).	N/A	95.3%
Johnston, N. 2016(71)												
16 cardiology departments	Sweden	174 patients with myocardial infarction (MI)	56.8 (8.0) vs 58.4 (8.6)	80.7%	N/A	131.1 (14.6) vs 125.2 (17.9)	Nurse, internists	Patient Education(6m) Received a complete interactive patient support tool (Web-based application (app) installed on their own smartphones.	Individual	4 main modules: Extended drug adherence e-diary, exercise, weight, and smoking modules. Additional general information regarding the cause, symptoms, and treatment of MI.	All device-related adverse effects and device deficiencies were registered during the study.	93.1%
Lear, S. A 2003(6)												
2 tertiary care hospitals	Canada	302 patients with ischemic heart disease	64.8 ± 8.8 vs 63.4 ± 10.2	82.5%	N/A	128.0 ± 21.0 vs 127.0 ± 20.0	Cardiologist, dietitian, exercise specialist	Patient Education(9m) Extensive Lifestyle Management Intervention : exercise sessions, telephone follow-ups and risk factor and lifestyle counselling.	Individual	Factors and lifestyle behaviours, drugs, compliance, and symptoms. Treatment algorithms for physical activity, diet, weight, diabetes, lipid, and blood pressure.	N/A	92.1%

Study Setting	Country	Study Population	Age	Gender Male	Socio-economic & Education Status	Baseline SBP	Personnel Involved	Freq & Duration of patient/personnel training	Patients per group	Curriculum	Quality Assurance	Attendance Rates/ Intensity
Sunamura, M 2018(72)												
10 hospitals	Netherlands	914 patients with ACS	57.1 ± 9.7 vs 57.4 ± 9.3	81.7%	Retired 56 (24.5%) vs 74 (30.4%) Intermediate High 222/230(96.5%) vs 232/244(95.1%)	119 (39.8%) vs 120 (39.2%)	Nurse, physiotherapist	Patient Education(12m) Group exercise programme(1.5-hour training sessions offered twice weekly for 12 weeks) + five to six individual telephone coaching sessions at 5 to 6-week intervals following standard CR.	Group & individual	Multifactor lifestyle, cardiovascular risk factor, develop a personal plan for a heart-healthy lifestyle.	N/A	75.0% vs 80.0%
Marcos-Forniol, E 2018(73)												
1 tertiary hospital	Barcelona	127 patients with acute coronary syndrome	76.0 (74.1-79.2) vs 75.4 (73.7-78.9)	60.2%	N/A	143.0 (123.0-157.0) vs 140.0 (122.0-157.0)	Multidisciplinary team (nurse, nutritionist and internist)	Patient Education(9m) Attend physician appointments every 3months (at the 3,6,9 months of the study) with a blood test performed the previous week.	Individual	Recommendations to improve lifestyle , adhere to a MedDiet and maintain physical function and changed the drug treatment if required to improve risk factor goals for CVD secondary prevention.	N/A	83.5%
Wolf, A 2016(74)												
2 hospitals	Sweden	199 patients with myocardial infarction or unstable angina pectoris	59.8 ± 10.1 vs 61.3 ± 8.9	75.3%	Employed 24.0 (65.0) vs 60.0 (57.1) High school above 31/37 (83.8%) vs 83/105 (79.0%)	N/A	Nurse, internists	Patient Education(2m) PCC + eHealth (Web-based or mobile-based eHealth tool for at least 2 months).	Individual	PCC plan: goals, expectations, and follow-up actions eHealth tool: Mobile app 3 modules: (1)Self-rated fatigue scale, (2) Symptom trend graph (3) Built-in accelerometer for physical activity level. Webpage 5 modules: (1) Self-rated symptoms of fatigue (2) Symptom trend graph, (3) Diary function for free-text entries (4) Chat function (5) Personal links to relevant webpages and to upload documents	N/A	98.0%

Study Setting	Country	Study Population	Age	Gender Male	Socio-economic & Education Status	Baseline SBP	Personnel Involved	Freq & Duration of patient/personnel training	Patients per group	Curriculum	Quality Assurance	Attendance Rates/ Intensity
Sturchio, A 2012(16)												
1 cardiac rehabilitation department	Italy	202 patient with coronary artery disease	56.0 ± 8.0 vs 56.0 ± 9.0	88.7%	N/A	125.0 ± 17.0 vs 123.0 ± 16.0	Cardiologist, trained nurses, physiotherapists	Patient Education(7m) Day Hospital Management Program (CARIMAP): cardiovascular risk stratification, tailored therapy, management of CAD risks, health education, counselling, physical training, multidisciplinary interventions, and outpatient FU counselling.	Individual	(1) Patient assessment (2) Review of cardiovascular risk factors (3) Goals of the program risk factor; home physical activity; and performance level of physical training.	N/A	83.2%
Fors, A 2017(75)												
2 hospitals	Sweden	252 patients with acute coronary syndrome	60.5 ± 9.3 vs 61.3 ± 8.9	72.5%	Employed 54(57.4) vs 60(57.1)	Hypertension 50 (53.2) vs 58 (55.8)	PCC teams (physician and registered nurse), cardiologist	Patient Education(6m) FU meeting at 4weeks post-discharge at the outpatient clinic & meeting within 8 weeks to one of five designated PCC teams. Personnel Education Specially trained through lectures, seminars and workshops.	Individual	3 routines for guiding of a PCC process to initiate, integrate and safeguard person-centred care in daily clinical practice.	N/A	98.0%
Wang 2019(9)												
301hospitals	United States	11, 001 patients with coronary artery disease	62.0 (54.0-70.0) vs 62.0 (54.0-69.0)	68.0%	Employed 3036 (47.2) vs 2111 (46.2) College Higher 2906 (47.4%) vs 2024 (51.0%)	Hypertension 4332 (67.3) vs 3243 (71.0)	Clinical care team	Patient Education(12m) Co-payment vouchers. Personnel Education Educated all clinicians with a pocket card describing the availability of a study voucher that provided co-payment reductions.	Individual	At the time of each medication fill or refill, the co-payment was charged to the study, resulting in zero out-of-pocket cost for the patient. For Medicare- or Medicaid-insured patients who cannot receive co-payment assistance, the voucher covered the entire cost of clopidogrel or ticagrelor prescriptions.	N/A	91.8%

Footnotes: AMI, acute myocardial infarction; CAD, coronary artery disease; CQI, continuous quality improvement; CR, cardiac rehabilitation; CV, cardiovascular; CVRF, cardiovascular risk factors; exCR, Exercise-based cardiac rehabilitation; freq, frequency; F2F, face-to-face; FU, follow up; GEx, guided training system; h,hour; m,month; N/A , not available; PA, physical activity; PCC, person-centered care; PCI, percutaneous coronary intervention; w,week

References

1. Lim LL, Lau ESH, Kong APS, et al. Aspects of Multicomponent Integrated Care Promote Sustained Improvement in Surrogate Clinical Outcomes: A Systematic Review and Meta-analysis. Diabetes Care. 2018;41(6):1312-20.

2. Berwanger O, Guimarães HP, Laranjeira LN, et al. Effect of a multifaceted intervention on use of evidence-based therapies in patients with acute coronary syndromes in Brazil: the BRIDGE-ACS randomized trial. Jama. 2012;307(19):2041-9.

3. Mayer-Berger W, Simic D, Mahmoodzad J, et al. Efficacy of a long-term secondary prevention programme following inpatient cardiovascular rehabilitation on risk and health-related quality of life in a low-education cohort: a randomized controlled study. Eur J Prev Cardiol. 2014;21(2):145-52.

4. Williams JB, Delong ER, Peterson ED, et al. Secondary prevention after coronary artery bypass graft surgery: findings of a national randomized controlled trial and sustained society-led incorporation into practice. Circulation. 2011;123(1):39-45.

5. The MEDMAN study: a randomized controlled trial of community pharmacy-led medicines management for patients with coronary heart disease. Family practice. 2007;24(2):189-200.

6. Lear SA, Ignaszewski A, Linden W, et al. The Extensive Lifestyle Management Intervention (ELMI) following cardiac rehabilitation trial. European Heart Journal. 2003;24(21):1920-7.

7. Ivers NM, Schwalm JD, Bouck Z, et al. Interventions supporting long term adherence and decreasing cardiovascular events after myocardial infarction (ISLAND): pragmatic randomised controlled trial. Bmj. 2020;369:m1731.

8. Munoz MA, Vila J, Cabañero M, et al. Efficacy of an intensive prevention program in coronary patients in primary care, a randomised clinical trial. Int J Cardiol. 2007;118(3):312-20.

9. Wang TY, Kaltenbach LA, Cannon CP, et al. Effect of Medication Co-payment Vouchers on P2Y12 Inhibitor Use and Major Adverse Cardiovascular Events Among Patients With Myocardial Infarction: The ARTEMIS Randomized Clinical Trial. Jama. 2019;321(1):44-55.

10. Minneboo M, Lachman S, Snaterse M, et al. Community-Based Lifestyle Intervention in Patients With Coronary Artery Disease: The RESPONSE-2 Trial. J Am Coll Cardiol. 2017;70(3):318-27.

11. Dalal HM, Evans PH, Campbell JL, et al. Home-based versus hospital-based rehabilitation after myocardial infarction: A randomized trial with preference arms--Cornwall Heart Attack Rehabilitation Management Study (CHARMS). Int J Cardiol. 2007;119(2):202-11.

12. Huffman MD, Mohanan PP, Devarajan R, et al. Effect of a Quality Improvement Intervention on Clinical Outcomes in Patients in India With Acute Myocardial Infarction: The ACS QUIK Randomized Clinical Trial. Jama. 2018;319(6):567-78.

13. Mosca L, Christian AH, Mochari-Greenberger H, Kligfield P, Smith SC, Jr. A randomized clinical trial of secondary prevention among women hospitalized with coronary heart disease. J Womens Health (Larchmt). 2010;19(2):195-202.

14. Yu C, Liu C, Du J, et al. Smartphone-based application to improve medication adherence in patients after surgical coronary revascularization. Am Heart J. 2020;228:17-26.

15. Redfern J, Briffa T, Ellis E, Freedman SB. Choice of secondary prevention improves risk factors after acute coronary syndrome: 1-year follow-up of the CHOICE (Choice of Health Options In prevention of Cardiovascular Events) randomised controlled trial. Heart. 2009;95(6):468-75.

16. Sturchio A, Di Gianni A, Campana B, et al. Coronary Artery RIsk MAnagement Programme (CARIMAP) delivered by a rehabilitation day-hospital: impact on patients with coronary artery disease. Journal of cardiopulmonary rehabilitation and prevention. 2012;32(6):386-93.

17. Jørstad HT, Minneboo M, Helmes HJ, et al. Effects of a nurse-coordinated prevention programme on health-related quality of life and depression in patients with an acute coronary

syndrome: results from the RESPONSE randomised controlled trial. BMC Cardiovasc Disord. 2016;16(1):144.

18. Lapointe F, Lepage S, Larrivée L, Maheux P. Surveillance and treatment of dyslipidemia in the post-infarct patient: can a nurse-led management approach make a difference? Can J Cardiol. 2006;22(9):761-7.

19. Carlsson R. Serum cholesterol, lifestyle, working capacity and quality of life in patients with coronary artery disease. Experiences from a hospital-based secondary prevention programme. Scand Cardiovasc J Suppl. 1998;50:1-20.

20. Maddison R, Rawstorn JC, Stewart RAH, et al. Effects and costs of real-time cardiac telerehabilitation: randomised controlled non-inferiority trial. Heart. 2019;105(2):122-9.

21. Duan YP, Liang W, Guo L, et al. Evaluation of a Web-Based Intervention for Multiple Health Behavior Changes in Patients With Coronary Heart Disease in Home-Based Rehabilitation: Pilot Randomized Controlled Trial. J Med Internet Res. 2018;20(11):e12052.

22. Hautala AJ, Kiviniemi AM, Mäkikallio T, et al. Economic evaluation of exercise-based cardiac rehabilitation in patients with a recent acute coronary syndrome. Scand J Med Sci Sports. 2017;27(11):1395-403.

23. Wang W, Lim JY, Lopez V, et al. The effect of a self-help psychoeducation programme for people with coronary heart disease: A randomized controlled trial. J Adv Nurs. 2018;74(10):2416-26.

24. Zhang P, Xing FM, Li CZ, Wang FL, Zhang XL. Effects of a nurse-led transitional care programme on readmission, self-efficacy to implement health-promoting behaviours, functional status and life quality among Chinese patients with coronary artery disease: A randomised controlled trial. J Clin Nurs. 2018;27(5-6):969-79.

25. Wu Y, Li S, Patel A, et al. Effect of a Quality of Care Improvement Initiative in Patients With Acute Coronary Syndrome in Resource-Constrained Hospitals in China: A Randomized Clinical Trial. JAMA Cardiol. 2019;4(5):418-27.

26. Ma L, Deng L, Yu H. The effects of a comprehensive rehabilitation and intensive education program on anxiety, depression, quality of life, and major adverse cardiac and cerebrovascular events in unprotected left main coronary artery disease patients who underwent coronary artery bypass grafting. Ir J Med Sci. 2020;189(2):477-88.

27. Santo K, Singleton A, Rogers K, et al. Medication reminder applications to improve adherence in coronary heart disease: a randomised clinical trial. Heart. 2019;105(4):323-9.

28. Wu Q, Zhang D, Zhao Q, et al. Effects of transitional health management on adherence and prognosis in elderly patients with acute myocardial infarction in percutaneous coronary intervention: A cluster randomized controlled trial. PLoS One. 2019;14(5):e0217535.

29. Chaves GSS, Lima de Melo Ghisi G, Britto RR, Grace SL. Maintenance of Gains, Morbidity, and Mortality at 1 Year Following Cardiac Rehabilitation in a Middle-Income Country: A Wait-List Control Crossover Trial. J Am Heart Assoc. 2019;8(4):e011228.

30. Zhang P, Hu YD, Xing FM, et al. Effects of a nurse-led transitional care program on clinical outcomes, health-related knowledge, physical and mental health status among Chinese patients with coronary artery disease: A randomized controlled trial. Int J Nurs Stud. 2017;74:34-43.

31. Lin CY, Yaseri M, Pakpour AH, et al. Can a Multifaceted Intervention Including Motivational Interviewing Improve Medication Adherence, Quality of Life, and Mortality Rates in Older Patients Undergoing Coronary Artery Bypass Surgery? A Multicenter, Randomized Controlled Trial with 18-Month Follow-Up. Drugs Aging. 2017;34(2):143-56.

32. Wang W, Jiang Y, He HG, Koh KW. A randomised controlled trial on the effectiveness of a home-based self-management programme for community-dwelling patients with myocardial infarction. Eur J Cardiovasc Nurs. 2016;15(6):398-408.

33. Alsaleh E, Windle R, Blake H. Behavioural intervention to increase physical activity in adults with coronary heart disease in Jordan. BMC Public Health. 2016;16:643.

34. Skobel E, Knackstedt C, Martinez-Romero A, et al. Internet-based training of coronary artery patients: the Heart Cycle Trial. Heart Vessels. 2017;32(4):408-18.

35. Melamed RJ, Tillmann A, Kufleitner H-E, Thürmer U, Dürsch M. Evaluating the efficacy of an education and treatment program for patients with coronary heart disease. Deutsches Arzteblatt international. 2014;111(47):802-8.

36. Vaillant A, Dentan G, Laurent Y, et al. The Log book for the secondary prevention of coronary artery disease: A pilot study. Presse Med. 2015;44(9):e301-9.

37. Seidl H, Hunger M, Leidl R, et al. Cost-effectiveness of nurse-based case management versus usual care for elderly patients with myocardial infarction: results from the KORINNA study. Eur J Health Econ. 2015;16(6):671-81.

38. O'Neil A, Taylor B, Hare DL, et al. Long-term efficacy of a tele-health intervention for acute coronary syndrome patients with depression: 12-month results of the MoodCare randomized controlled trial. Eur J Prev Cardiol. 2015;22(9):1111-20.

39. Holmes-Rovner M, Stommel M, Corser WD, et al. Does outpatient telephone coaching add to hospital quality improvement following hospitalization for acute coronary syndrome? J Gen Intern Med. 2008;23(9):1464-70.

40. Wang W, Chair SY, Thompson DR, Twinn SF. Effects of home-based rehabilitation on health-related quality of life and psychological status in Chinese patients recovering from acute myocardial infarction. Heart Lung. 2012;41(1):15-25.

41. Hawkes AL, Patrao TA, Atherton J, et al. Effect of a telephone-delivered coronary heart disease secondary prevention program (proactive heart) on quality of life and health behaviours: primary outcomes of a randomised controlled trial. Int J Behav Med. 2013;20(3):413-24.

42. Young W, Rewa G, Goodman SG, et al. Evaluation of a community-based inner-city disease management program for postmyocardial infarction patients: a randomized controlled trial. Cmaj. 2003;169(9):905-10.

43. Chair SY, Chan SW, Thompson DR, et al. Long-term effect of motivational interviewing on clinical and psychological outcomes and health-related quality of life in cardiac rehabilitation patients with poor motivation in Hong Kong: a randomized controlled trial. Clin Rehabil. 2013;27(12):1107-17.

44. Carroll DL, Rankin SH, Cooper BA. The effects of a collaborative peer advisor/advanced practice nurse intervention: cardiac rehabilitation participation and rehospitalization in older adults after a cardiac event. J Cardiovasc Nurs. 2007;22(4):313-9.

45. Hanssen TA, Nordrehaug JE, Eide GE, Hanestad BR. Does a telephone follow-up intervention for patients discharged with acute myocardial infarction have long-term effects on health-related quality of life? A randomised controlled trial. J Clin Nurs. 2009;18(9):1334-45.

46. Tingström PR, Kamwendo K, Bergdahl B. Effects of a problem-based learning rehabilitation programme on quality of life in patients with coronary artery disease. Eur J Cardiovasc Nurs. 2005;4(4):324-30.

47. Zhao Y, Wong FK. Effects of a postdischarge transitional care programme for patients with coronary heart disease in China: a randomised controlled trial. J Clin Nurs. 2009;18(17):2444-55.

48. Du L, Dong P, Jia J, et al. Impacts of intensive follow-up on the long-term prognosis of percutaneous coronary intervention in acute coronary syndrome patients - a single center prospective randomized controlled study in a Chinese population. Eur J Prev Cardiol. 2016;23(10):1077-85.

49. Yu CM, Lau CP, Chau J, et al. A short course of cardiac rehabilitation program is highly cost effective in improving long-term quality of life in patients with recent myocardial infarction or percutaneous coronary intervention. Arch Phys Med Rehabil. 2004;85(12):1915-22.

50. Murphy BM, Worcester MU, Higgins RO, et al. Reduction in 2-year recurrent risk score and improved behavioral outcomes after participation in the "Beating Heart Problems" self-management program: results of a randomized controlled trial. J Cardiopulm Rehabil Prev. 2013;33(4):220-8.

51. Katalinic A, Waldmann A, Schwaab B, et al. The TeleGuard trial of additional telemedicine care in CAD patients. 1 Utilization of the system. J Telemed Telecare. 2008;14(1):17-21.

52. Briffa TG, Eckermann SD, Griffiths AD, et al. Cost-effectiveness of rehabilitation after an acute coronary event: a randomised controlled trial. Med J Aust. 2005;183(9):450-5.

53. Sinclair AJ, Conroy SP, Davies M, Bayer AJ. Post-discharge home-based support for older cardiac patients: a randomised controlled trial. Age Ageing. 2005;34(4):338-43.

54. Vale MJ, Jelinek MV, Best JD, et al. Coaching patients On Achieving Cardiovascular Health (COACH): a multicenter randomized trial in patients with coronary heart disease. Arch Intern Med. 2003;163(22):2775-83.

55. Arthur HM, Daniels C, McKelvie R, Hirsh J, Rush B. Effect of a preoperative intervention on preoperative and postoperative outcomes in low-risk patients awaiting elective coronary artery bypass graft surgery. A randomized, controlled trial. Ann Intern Med. 2000;133(4):253-62.

56. Goodman H, Parsons A, Davison J, et al. A randomised controlled trial to evaluate a nurse-led programme of support and lifestyle management for patients awaiting cardiac surgery 'Fit for surgery: Fit for life' study. Eur J Cardiovasc Nurs. 2008;7(3):189-95.

57. Jolly K, Bradley F, Sharp S, et al. Randomised controlled trial of follow up care in general practice of patients with myocardial infarction and angina: final results of the Southampton heart integrated care project (SHIP). The SHIP Collaborative Group. Bmj. 1999;318(7185):706-11.

58. Brügemann J, Poels BJ, Oosterwijk MH, et al. A randomised controlled trial of cardiac rehabilitation after revascularisation. Int J Cardiol. 2007;119(1):59-64.

59. Capone RJ, Stablein D, Visco J, et al. The effects of a transtelephonic surveillance and prehospital emergency intervention system on the 1-year course following acute myocardial infarction. Am Heart J. 1988;116(6 Pt 1):1606-15.

60. Rollman BL, Belnap BH, LeMenager MS, et al. Telephone-delivered collaborative care for treating post-CABG depression: a randomized controlled trial. Jama. 2009;302(19):2095-103.

61. Kripalani S, Schmotzer B, Jacobson TA. Improving Medication Adherence through Graphically Enhanced Interventions in Coronary Heart Disease (IMAGE-CHD): a randomized controlled trial. J Gen Intern Med. 2012;27(12):1609-17.

62. Tranmer JE, Parry MJ. Enhancing postoperative recovery of cardiac surgery patients: a randomized clinical trial of an advanced practice nursing intervention. West J Nurs Res. 2004;26(5):515-32.

63. Heller RF, Knapp JC, Valenti LA, Dobson AJ. Secondary prevention after acute myocardial infarction. Am J Cardiol. 1993;72(11):759-62.

64. Bolman C, Brug J, Bär F, Martinali J, van den Borne B. Long-term efficacy of a checklist to improve patient education in cardiology. Patient Educ Couns. 2005;56(2):240-8.

65. Furze G, Dumville JC, Miles JN, et al. "Prehabilitation" prior to CABG surgery improves physical functioning and depression. Int J Cardiol. 2009;132(1):51-8.

66. Dracup K, McKinley S, Riegel B, et al. A randomized clinical trial to reduce patient prehospital delay to treatment in acute coronary syndrome. Circ Cardiovasc Qual Outcomes. 2009;2(6):524-32.

67. Keeping-Burke L, Purden M, Frasure-Smith N, et al. Bridging the transition from hospital to home: effects of the VITAL telehealth program on recovery for CABG surgery patients and their caregivers. Res Nurs Health. 2013;36(6):540-53.

68. Varnfield M, Karunanithi M, Lee CK, et al. Smartphone-based home care model improved use of cardiac rehabilitation in postmyocardial infarction patients: results from a randomised controlled trial. Heart. 2014;100(22):1770-9.

69. Leemrijse CJ, Peters RJ, von Birgelen C, et al. The telephone lifestyle intervention 'Hartcoach' has modest impact on coronary risk factors: A randomised multicentre trial. Eur J Prev Cardiol. 2016;23(15):1658-68.

70. Ho PM, Lambert-Kerzner A, Carey EP, et al. Multifaceted intervention to improve medication adherence and secondary prevention measures after acute coronary syndrome hospital discharge: a randomized clinical trial. JAMA internal medicine. 2014;174(2):186-93.

71. Johnston N, Bodegard J, Jerström S, et al. Effects of interactive patient smartphone support app on drug adherence and lifestyle changes in myocardial infarction patients: A randomized study. American Heart Journal. 2016;178:85-94.

72. Sunamura M, Ter Hoeve N, Van Den Berg-Emons RJG, et al. Randomised controlled trial of two advanced and extended cardiac rehabilitation programmes. Heart. 2018;104(5):430-7.

73. Marcos-Forniol E, Meco JF, Corbella E, Formiga F, Pinto X. Secondary prevention programme of ischaemic heart disease in the elderly: A randomised clinical trial. European journal of preventive cardiology. 2018;25(3):278-86.

74. Wolf A, Fors A, Ulin K, et al. An eHealth Diary and Symptom-Tracking Tool Combined With Person-Centered Care for Improving Self-Efficacy After a Diagnosis of Acute Coronary Syndrome: A Substudy of a Randomized Controlled Trial. Journal of medical Internet research. 2016;18(2):e40.

75. Fors A, Swedberg K, Ulin K, Wolf A, Ekman I. Effects of person-centred care after an event of acute coronary syndrome: Two-year follow-up of a randomised controlled trial. Int J Cardiol. 2017;249:42-7.
